# Electrochemically promoted site-selective selenylation at the C-4 position of pyrazolones

**DOI:** 10.1039/d6ra01336h

**Published:** 2026-07-27

**Authors:** Linyu Zheng, Wei Zhong, Zixun Gao, Bintao Liu, Siao Lu, Yulin Feng, Fangling Lu

**Affiliations:** a The National Pharmaceutical Engineering Center for Solid Preparation in Chinese Herbal Medicine, Jiangxi University of Chinese Medicine 56 Yangming Road Jiangxi Nanchang 330006 P. R. China fengyulin2003@126.com 20211036@jxutcm.edu.cn

## Abstract

The late-stage functionalization of drug molecules represents a significant advancement in medicinal chemistry. We herein describe an electrochemical strategy for site-selective selenylation at the C-4 position of pyrazolones under transition-metal- and oxidant-free conditions. This method exhibits broad substrate scope, accommodating a variety of pyrazolones and selenium sulfonates, and delivers the corresponding products in up to 92% yield. Using selenium sulfonates as selenylating reagents, this approach provides a practical route for introducing selenium into bioactive pyrazolone scaffolds, demonstrating considerable potential in drug diversification.

## Abstract 2

The late-stage functionalization of drug molecules represents a significant advancement in medicinal chemistry.

## Graphical abstract

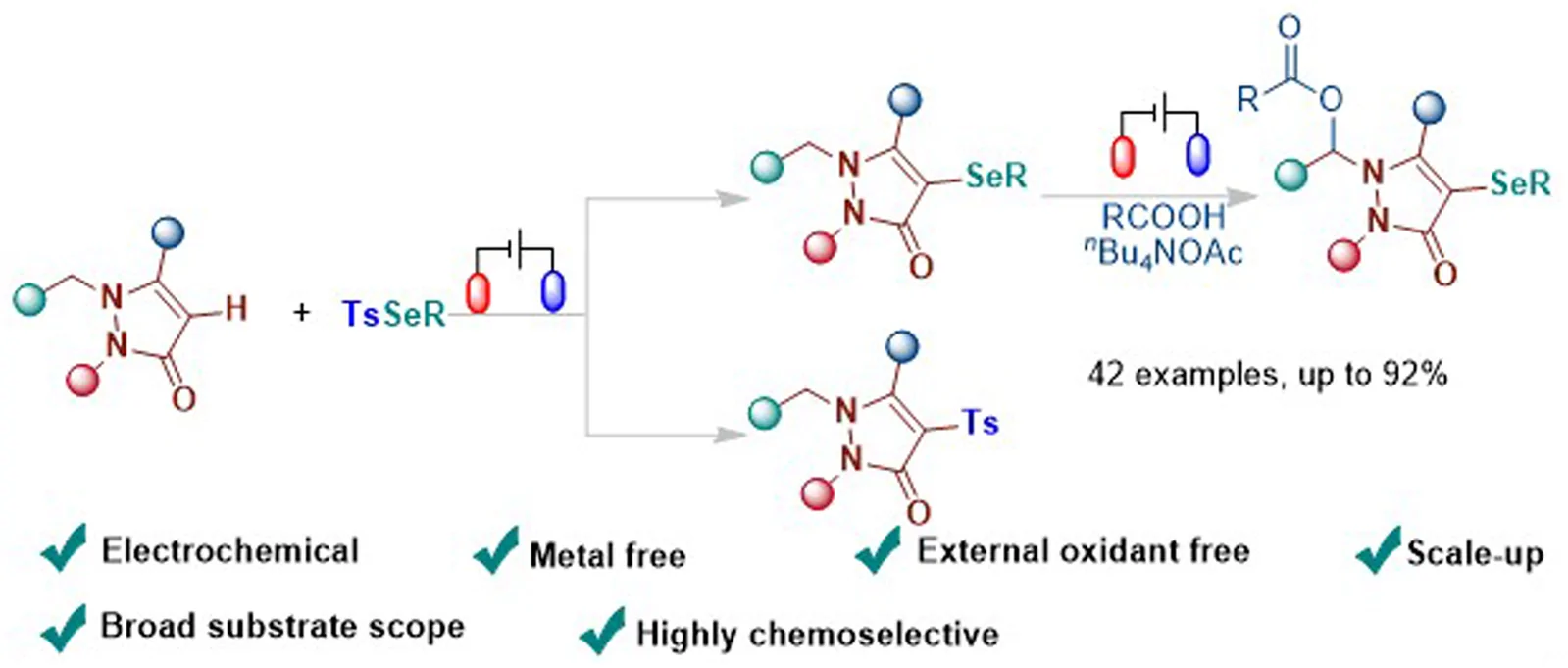

Pyrazolones represent an important class of five-membered aza-heterocyclic compounds with diverse pharmacological activities, including antioxidant, antitumor, analgesic, anti-inflammatory, antiviral and antibacterial effects.^1^ A representative example is antipyrine and its clinically used derivatives such as aminopyrine, metamizole sodium, and famprofazone, all of which are valuable NSAIDs widely employed for managing fever and inflammation (Scheme 1a).^2^ As privileged scaffolds in medicinal chemistry, pyrazolones have attracted considerable research interest toward their functionalization. Numerous methods have been developed for direct C–H functionalization at the C-4 position, enabling the construction of C–C,^3^ C–S/Se,^4^ C–P,^5^ C–O,^6^ and C–X^7^ bonds. However, these methods often face challenges including limited substrate scope, harsh reaction conditions, and unwanted by-products. Moreover, most existing protocols rely on expensive transition metals or stoichiometric oxidants, resulting in high costs and additional byproduct formation. Notably, direct sulfonylation at the C-4 position of pyrazolones has not been achieved, representing a significant gap in current methodology.

**Scheme 1:**
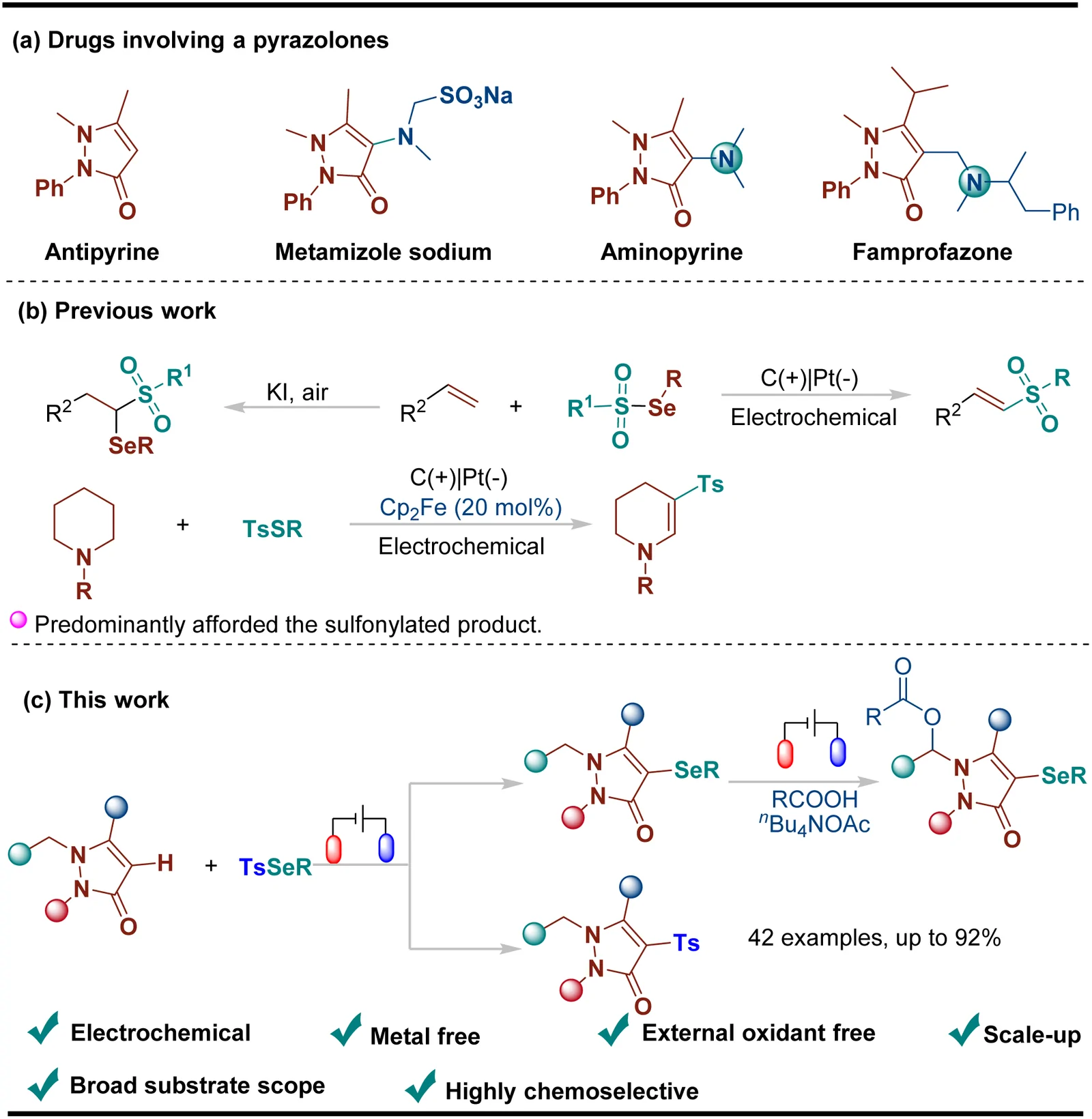
(a) Drugs with pyrazolone structures, (b) previous work, (c) electrochemically promoted site-selective selenylation at the C-4 position of pyrazolones.

Organic electrosynthesis utilizes electrons as traceless reagents to replace stoichiometric redox agents.^8^ This approach has proven effective for facilitating C(sp^2^)–H bond functionalization.^9^ Significant progress has recently been made in the electrochemical construction of C–S and C–Se bonds using selenium sulfonates (R^1^SO_2_SeR^2^) as selenylating and sulfonylating reagents.^10^ For example, in 2024, Cao and co-workers developed a controllable method for synthesizing vinyl sulfones and β-keto selenosulfones *via* electrochemical oxidative sulfonylation of alkenes.^10*a*^ That same year, Ling and co-workers^10*b*^ realized an electrochemically enabled desaturated β-C(sp^3^)–H sulfonylation of cyclic amines. Our group has previously reported electrochemical C–Se bond formation in pyrazolones using diselenides.^11^ In the present study, we extend this approach by employing PhSeTs as an alternative selenium source, which offers advantages such as improved stability and controlled electrophilic reactivity compared to diselenides, while also providing high product yields.^12^ To date, the use of R^1^SO_2_SeR^2^ reagents for the electrochemical C–H functionalization of pyrazolone derivatives has not been extensively explored.

Building on our sustained interest in sustainable electrochemistry, we describe an electrochemical method for site-selective selenylation at the C-4 position of pyrazolones. The key features of this method include: (i) electricity serves as an oxidant, avoiding the use of stoichiometric traditional oxidants; (ii) the metal- and base-free conditions offer operational simplicity; (iii) mild conditions, a broad substrate scope (42 examples), and scalability (up to 5.0 mmol) underscore its practical utility; and (iv) the reaction provides opportunities for further derivatization.

The initial investigation was carried out using antipyrine (1a) and PhSeTs (2a) as model substrates. A set of optimal conditions was identified, which involved an undivided cell equipped with a graphite rod anode and a platinum plate cathode, performing electrolysis at a constant current of 12 mA (5.3 mA cm^−2^) in acetonitrile containing ^*n*^Bu_4_NBF_4_ as the electrolyte. After 5 hours, the target product 3a was isolated in 87% yield (Table 1, entry 1). The reaction efficiency was sensitive to the applied current density. When the constant current was lowered from 12 mA to 8 mA (3.6 mA cm^−2^), the yield dropped to 70% (Table 1, entry 3). Conversely, increasing the current to 20 mA (8.9 mA cm^−2^), also adversely affected the reaction, reducing the yield to 64% (Table 1, entry 2). Several alternative solvents, including DMF, DMSO, and CH_3_OH, were evaluated; however, all proved less effective, leading to inferior yields (entries 4–6). The choice of electrode material was also crucial. Substituting the platinum cathode with stainless steel or Ni plates, or using Pt as the anode, resulted in lower yields (Table 1, entries 7–9). Subsequently, the influence of the electrolyte was investigated. Substitution of ^*n*^Bu_4_NBF_4_ with ^*n*^Bu_4_NOAc, ^*n*^Bu_4_NPF_6_, ^*n*^Bu_4_NI, or ^*n*^Bu_4_NBr consistently gave inferior yields (entries 10–13). The reaction was found to proceed under an air atmosphere, affording the desired product in 65% yield (Table 1, entry 14). Finally, a control experiment conducted without electrical current afforded only 23% of the product, confirming the essential role of the electrochemical setup in achieving efficient conversion (Table 1, entry 15).

**Table 1:** Optimization of the reaction conditions^a^

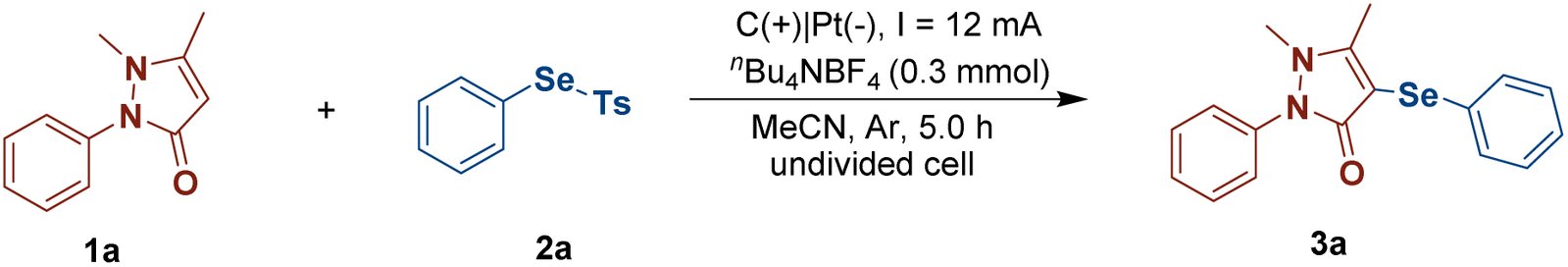		
Entry	Variation from the standard conditions	Yield^b^ (%)
1	None	87
2	20 mA (8.9 mA cm^−2^), 3.0 h	64
3	8 mA (3.6 mA cm^−2^), 7.5 h	70
4	DMF instead of MeCN	46
5	DMSO instead of MeCN	24
6	MeOH instead of MeCN	35
7	C (+)|SS (–) instead of C (+)|Pt (–)	40
8	C (+)|Ni (–) instead of C (+)|Pt (–)	51
9	Pt (+)|Pt (–) instead of C (+)|Pt (–)	67
10	^*n*^Bu_4_NOAc instead of ^*n*^Bu_4_NBF_4_	53
11	^*n*^Bu_4_NPF_6_ instead of ^*n*^Bu_4_NBF_4_	75
12	^*n*^Bu_4_NI instead of ^*n*^Bu_4_NBF_4_	68
13	^*n*^Bu_4_NBr instead of ^*n*^Bu_4_NBF_4_	55
14	Under air	65
15	No electric current	23

a Reaction conditions: 1a (0.45 mmol), 2a (0.3 mmol), ^*n*^Bu_4_NBF_4_ (0.3 mmol), MeCN (7 mL), graphite rod as the anode (*ϕ* 6 mm, about 15 mm immersion depth in solution) and platinum plate (15 mm × 15 mm × 0.3 mm) as the cathode, undivided cell, 12 mA (5.3 mA cm^−2^), Ar, 5.0 h.

b isolated yield. n.d. = not detected.

Under the optimized electrochemical conditions, we proceeded to investigate the substrate generality of this pyrazolone functionalization reaction (Scheme 2). The system displayed wide functional group compatibility, effectively accommodating pyrazolone scaffolds decorated with diverse aryl and alkyl substituents at both carbon and nitrogen sites. Pyrazolones featuring electron-donating arene substituents at R^1^, including *p*-Me (3b), *p*-Et (3c), *m*-Me (3d), 3,4-dimethyl (3e), and 2,4-dimethyl (3f), were converted to the desired products in 77–92% yields. Halogen-functionalized variants, such as *p*-F (3g), *o*-Cl (3h), *m*-Cl (3i), *p*-Br (3j), *m*-Br (3k), *p*-I (3l), 2,4-dichloro (3m), 3,5-dichloro (3n), 2,5-difluoro (3o), and pentafluoro (3p) also reacted smoothly, offering potential sites for further derivatization. Strong electron-withdrawing groups, exemplified by *p*-CF_3_ (3q), *m*-CF_3_ (3r), and *p*-CN (3s), were likewise well accommodated, leading to high yields of the corresponding products. Notably, substrates bearing a naphthyl (3t) or alkyl group (3u, 3v) at the R^1^ position were also viable, affording the desired products in moderate yields. Additionally, the method was extended to the selenation of pyrazolones bearing various alkyl and benzyl groups at the R^2^ and R^3^ positions (3u–3zd), producing the expected compounds in moderate to good yields. Moreover, when the R^2^ position was substituted with drug (3z) or pesticide residues (3za), the corresponding selenylated products were obtained in satisfactory yields, underscoring the utility of this method for the late-stage functionalization of complex bioactive scaffolds. To further demonstrate the versatility of this methodology, we explored a set of selenium-containing sulfonate substrates. A range of Se-aryl and Se-alkyl selenium sulfonates participated effectively in the transformation, affording products 3ze–3zj in 35–85% yields.

**Scheme 2:**
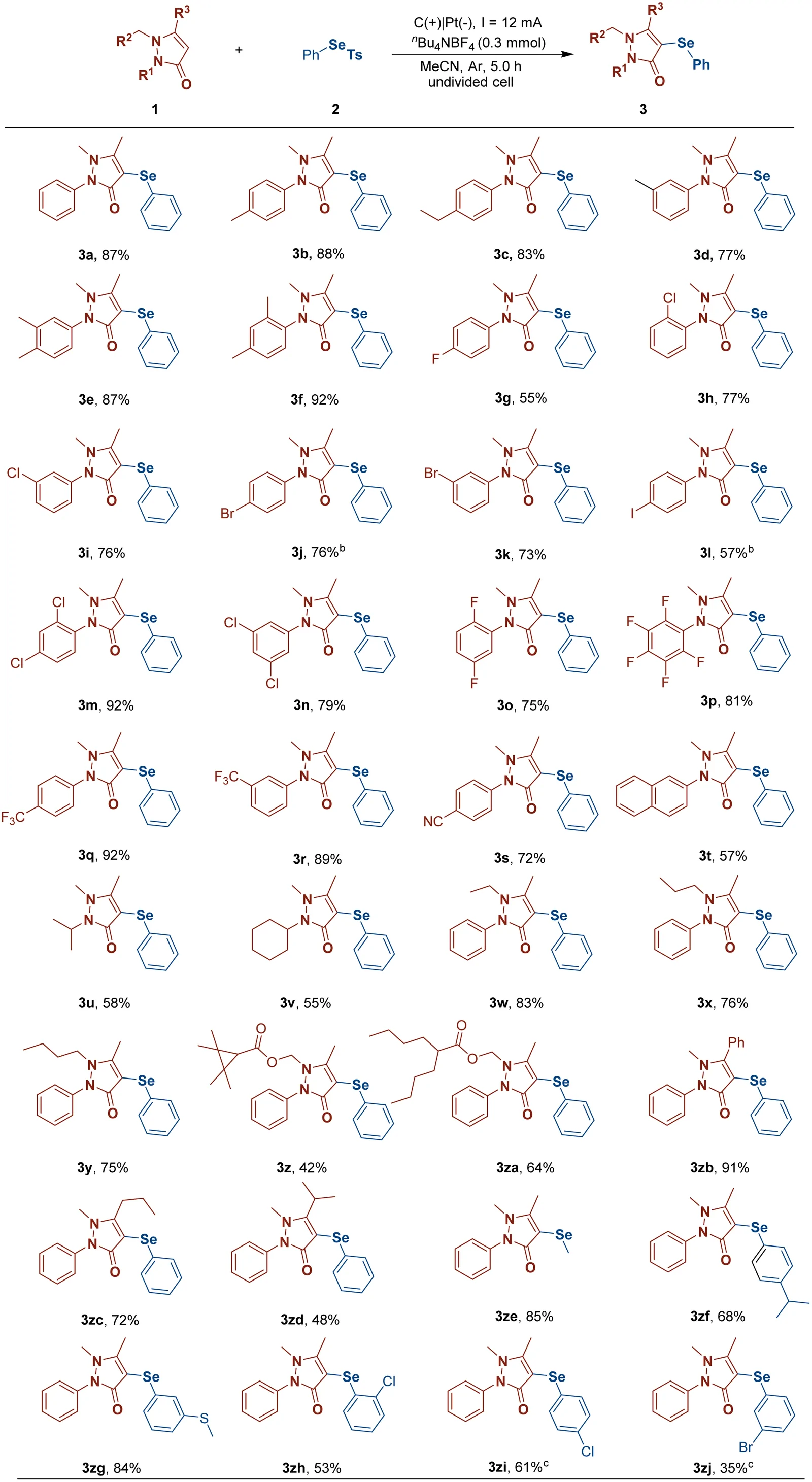
Substrate scope of pyrazolones and selenium sulfonates: ^*a*^reaction conditions: 1 (0.45 mmol), 2 (0.3 mmol), ^*n*^Bu_4_NBF_4_ (0.3 mmol) in CH_3_CN (7 mL), graphite rod as the anode (*ϕ* 6 mm, about 15 mm immersion depth in solution) and platinum plate (15 mm × 15 mm × 0.3 mm) as the cathode, undivided cell, r.t., Ar, 5.3 mA cm^−2^, 5.0 h. ^*b*^ 5.3 mA cm^−2^, 4 h. ^*c*^ MeSO_2_SeR as selenium source.

We tested the scalability of this electrochemical, site-selective selenylation reaction by scaling it up to 5.0 mmol. The reaction between 1a and 2a provided product 3a in 83% yield (Scheme 3a), demonstrating virtually no loss in efficiency compared to the 0.5 mmol-scale experiment. We then evaluated PhSeTs as sulfonylating agents under modified conditions, which provided the sulfonylated product 4a in 16% yield (Scheme 3b). Although the efficiency remains low, this outcome represents a significant advance in the construction of sulfonylated antipyrine derivatives.

**Scheme 3:**
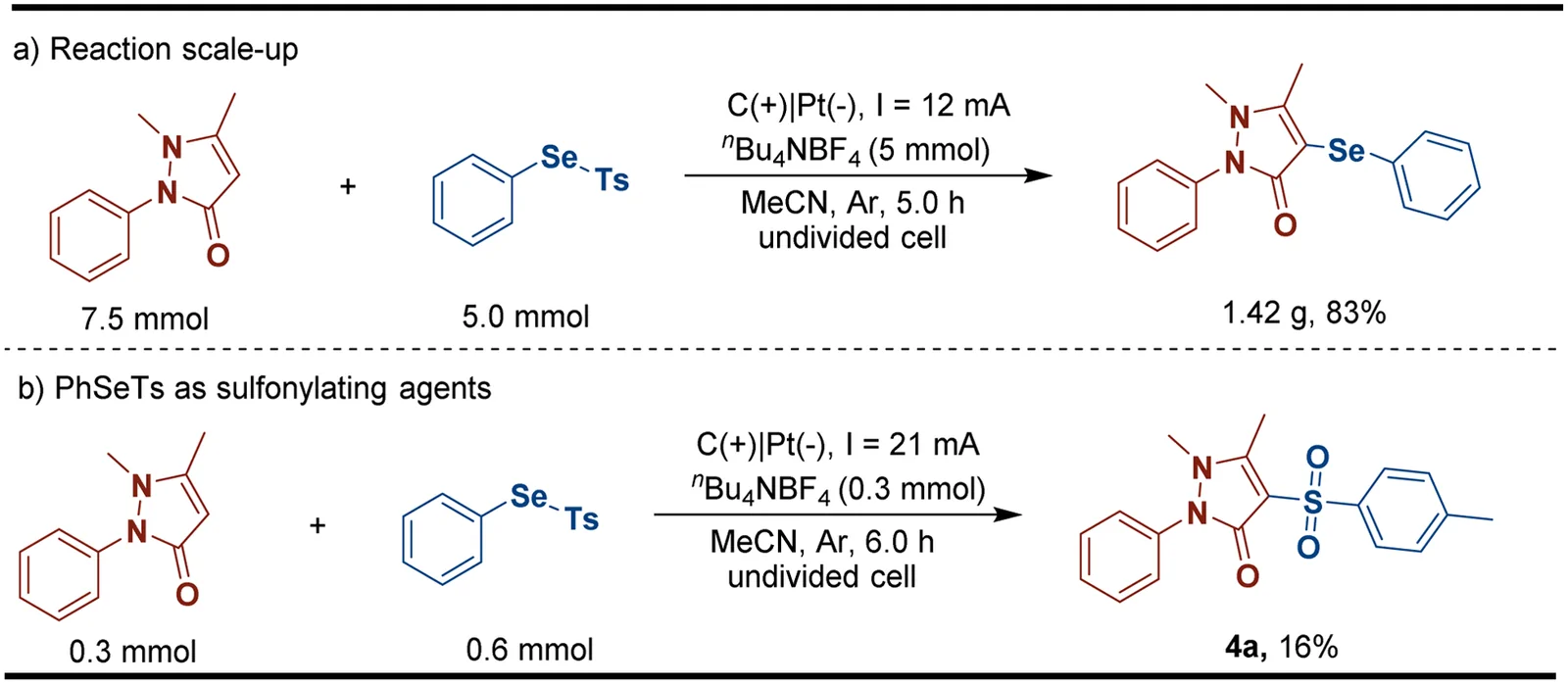
Gram-scale synthesis and derivatizations.

Finally, to further demonstrate the applicability of this reaction, we explored its derivatization potential (Scheme 4).^13^ It was found that compounds of type 3a could undergo Csp^3^-H esterification with a variety of carboxylic acids, such as para-Ph (5a), 1-naphthoic acid (5b), deuterated benzoic acid (5c)and *p*-Cl (5d). In all cases, the corresponding esterified products were obtained in moderate to excellent yields. Furthermore, tetrabutylammonium acetate (5e) was also compatible under the reaction conditions, affording the desired ester in 45% yield.

**Scheme 4:**
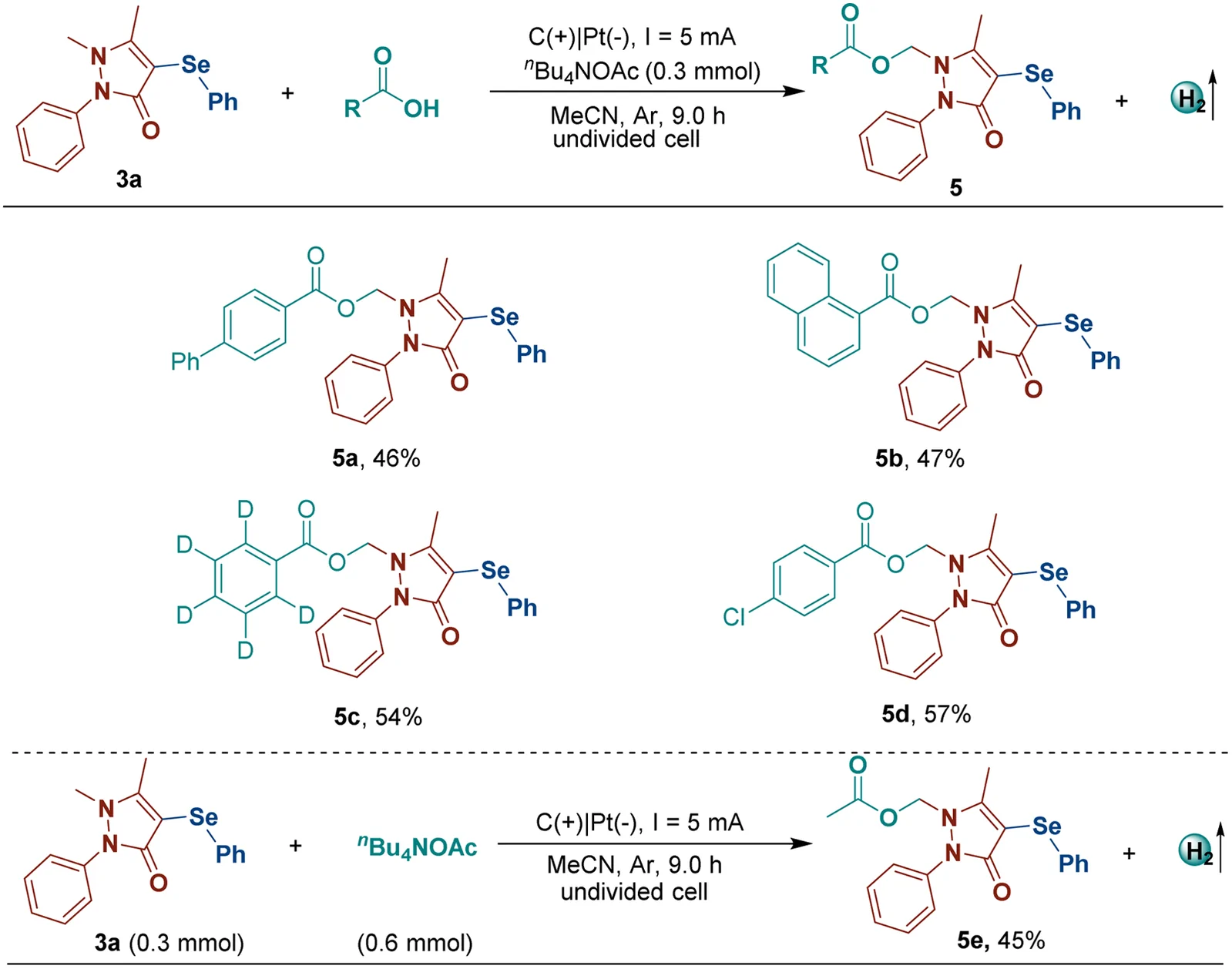
^*a*^Reaction conditions: 3a (0.6 mmol), carboxylic acids (0.3 mmol), ^*n*^Bu_4_NOAc (0.3 mmol) in CH_3_CN (9 mL), graphite rod as the anode (*ϕ* 6 mm, about 15 mm immersion depth in solution) and platinum plate (15 mm × 15 mm × 0.3 mm) as the cathode, undivided cell, r.t., Ar, 2.2 mA cm^−2^, 9.0 h.

To gain deeper insights into the reaction mechanism, controlled experiments and cyclic voltammetry analyses were conducted (Scheme 5). Upon addition of 2.0 equiv. of 1,1-diphenylethylene to the reaction mixture, the formation of the DPE-Ts adduct 7a was confirmed by LC-MS analysis. However, no selenium radical intermediates, such as 8a, were detected, which is in contrast to our previously reported electrochemical selenylation of pyrazolones^11]^. In contrast, the use of PhSeCl under non-electrochemical conditions afforded the desired product in 96% yield, supporting the involvement of PhSe^+^ as the key intermediate. Furthermore, when using Se-(*p*-tolyl) 4-methylbenzenesulfonoselenoate as the substrate, LC-MS enabled the detection of product 9a, which arises from sulfonyl radical reduction (see SI for details).

**Scheme 5:**
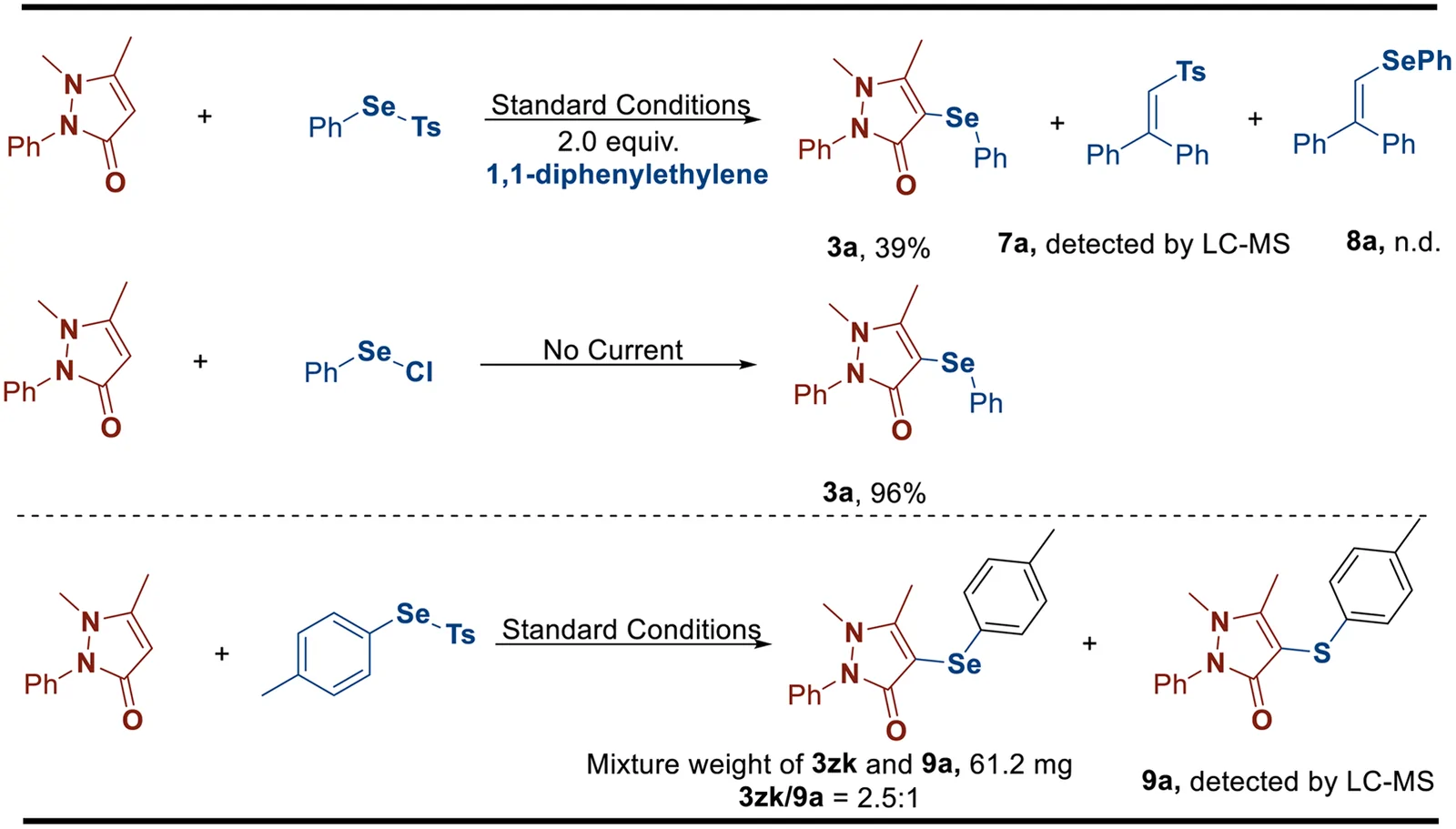
Control experiments.

Cyclic voltammetry measurements were performed with the half-wave potential of the ferrocene/ferrocenium (Fc/Fc^+^) couple measured as +0.096 V *versus* the non-aqueous Ag/Ag^+^ reference electrode (Fig. 1A). Under oxidative conditions (Fig. 1B), 1a displays an oxidation peak at +0.87 V *vs.* Fc/Fc^+^, whereas 2a shows an oxidation peak at +1.67 V *vs.* Fc/Fc^+^, suggesting that 1a is more readily oxidized than 2a. Under reductive conditions (Fig. 1C), 2a exhibits reduction peaks at −1.06 V and −1.53 V *vs.* Fc/Fc^+^, while 1a shows no well-defined reduction peak within the scanned potential range down to −3.0 V. This marked difference indicates that 2a is preferentially reduced at the cathode over 1a. Therefore, we conclude that 2a preferentially undergoes reduction rather than oxidation under our reaction conditions.

**Fig. 1:**
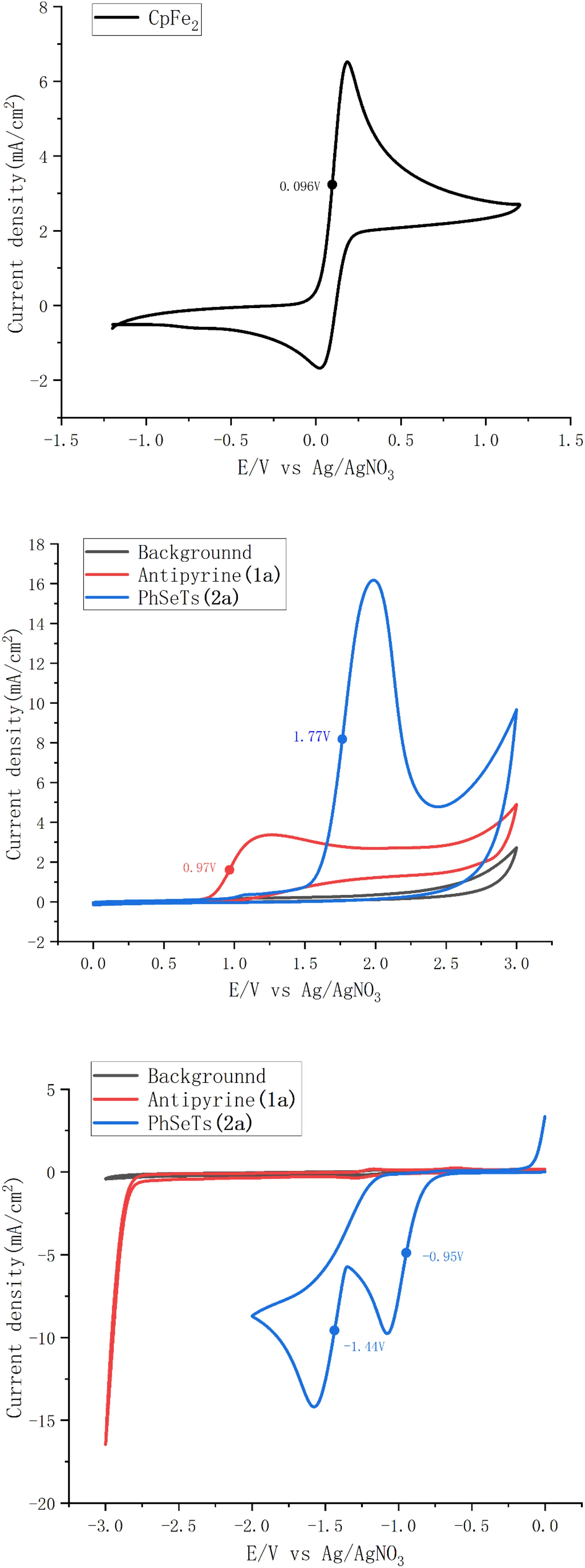
Cyclic voltammetry conditions: 0.1 M ^*n*^Bu_4_NBF_4_ in MeCN under Ar; glassy carbon working electrode (*ϕ* 3 mm), Pt plate counter electrode (15 mm × 15 mm × 0.3 mm, 99.99% purity), Non-aqueous reference electrode (0.1 M ^*n*^Bu_4_NBF_4_ + 0.01 M AgNO_3_ in MeCN); scan rate 0.1 V s; potential scanned from 0 V to 3.0 V (oxidation direction) and from 0 V to −3.0 V (reduction direction).

Based on the above results and previous reports, we propose a plausible mechanism as illustrated in Scheme 6. Initially, 2a undergoes reduction at the cathode to form a radical anion intermediate D, which subsequently dissociates into a sulfonyl radical (F) and a phenylselenium anion (E). The phenylselenium anion (E) is successively oxidized to the phenylselenium cation, which then combines with 1a to form a reversible seleniranium intermediate G, and upon deprotonation, yields the final product 3a. Meanwhile, the sulfonyl radical (F) is reduced to generate a sulfur radical (H). Concurrently, antipyrine is oxidized at the anode to generate a radical cation intermediate A, which isomerizes to the corresponding carbon radical cation intermediate B. A radical/radical cross-coupling reaction between the sulfonyl/sulfur radical and carbon radical cation B affords intermediate C and I respectively. Finally, the desired products 5a and 9a are obtained *via* deprotonation and isomerization.

**Scheme 6:**
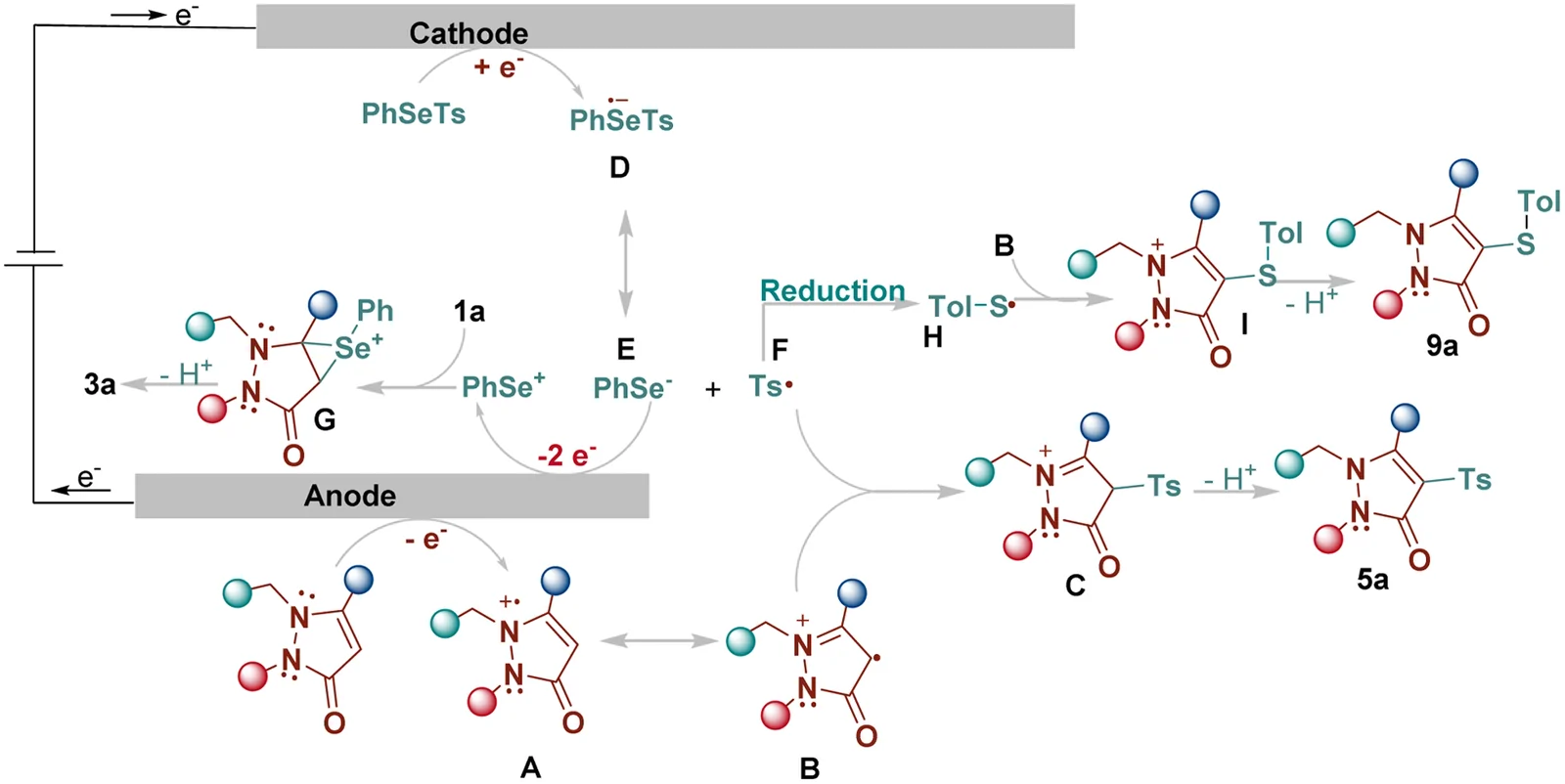
Proposed reaction mechanism.

In summary, we have developed a novel electrochemical C–H functionalization strategy for the direct, site-selective selenylation of pyrazolones. Utilizing selenium sulfonates (R^1^SO_2_SeR^2^) as reagents, this method operates under mild conditions without the need for transition metals, oxidants, or bases, and exhibits excellent functional group tolerance. Furthermore, the successful gram-scale synthesis and further derivatization reactions demonstrate the potential of this approach for practical application. Building on this foundation, electrochemical reactions employing selenium sulfonates are under further investigation to explore their broader utility in organic synthesis.

## Experimental section

The reaction was conducted in an oven-dried 15 mL undivided three-necked flask. First, pyrazolones (0.3 mmol), RSeTs (0.45 mmol), ^*n*^Bu_4_NBF_4_ (0.3 mmol), and MeCN (7 mL) were introduced into the flask containing a stir bar. The flask was then configured with a platinum cathode (1.5 cm × 1.5 cm × 0.3 mm) and a graphite rod anode (*Φ* 6 mm, about 15 mm immersion depth in solution), followed by argon purging. The reaction mixture was stirred under electrolysis at a constant current of 12 mA (5.3 mA cm^−2^) for 5.0 h. After full conversion (verified by TLC and LC-MS), the crude mixture was purified by flash chromatography on silica gel (eluent: petroleum ether/ethyl acetate) to afford the pure product.

## Conflicts of interest

The authors declare that they have no conflict of interest.

## Supplementary Material

RA-016-D6RA01336H-s001

## Data Availability

The data supporting this article have been included as part of the supplementary information (SI). Supplementary information is available. See DOI: https://doi.org/10.1039/d6ra01336h.
