# Supplementary material for: Electrochemically promoted site-selective selenylation at the C-4 position of pyrazolones
Source: RSC Adv. 2026 Jul 27. Online ahead of print. doi: 10.1039/d6ra01336h (PMC13404192; doi:10.1039/d6ra01336h)

Supplementary Information (SI) for RSC Advances.
This journal is © The Royal Society of Chemistry 2026

Supporting Information

Electrochemically Promoted Site-Selective Selenylation at the C-4 Position of Pyrazolones

Linyu Zheng[a], Wei Zhong[a], Zixun Gao[a], Bintao Liu[a], Siao Lu[a], Yulin Feng[*a], Fangling Lu[*a]

[a]The National Pharmaceutical Engineering Center for Solid Preparation in Chinese Herbal Medicine, Jiangxi University of Chinese Medicine, 56 Yangming Road, Jiangxi, Nanchang 330006, P. R. China.

Table of Contents

General information……………………………………………………….. S3

Experimental procedure………………………………………………………S4

Mechanism research………………………………………………………..S5

Detail descriptions for products………………………………………S7

References…………………………………………….……….……….. S16

Copies of ^{1}H NMR, ^{13}C NMR and ^{19}F NMR spectra……………..... S17

General information

Unless otherwise noted, materials were obtained from commercial suppliers and used without further purification. The instrument for electrolysis was dual display potentiostat (DJS-292B) (made in China). The anodic electrode was graphite rod (ϕ 6 mm, 98% purity, purchased from Wuhan Gaosiruilian Technology Co., Ltd (http://www.gaossunion.com/productinfo/490054.html)) and cathodic electrode was platinum plate (15 mm×15 mm×0.3 mm, pt 1515-3, 99.99% purity, purchased from Wuhan Gaosiruilian Technology Co., Ltd). Non-aqueous reference electrode (CHI112, purchased from Shanghai Chenhua Instruments Co., Ltd.), glassy carbon electrode (ϕ 3 mm, CHI104, purchased from Shanghai Chenhua Instruments Co., Ltd (https://www.chinstr.com)), platinum wire (CHI115, ϕ 0.5 mm, 30 mm length, purchased from Shanghai Chenhua Instruments Co., Ltd.) and electrochemical workstation (CHI760F, purchased from Shanghai Chenhua Instruments Co., Ltd.). Thin layer chromatography (TLC) employed glass 0.25 mm silica gel plates. Flash chromatography columns were packed with 300-400 mesh silica gel in petroleum (boiling point was between 60-90 ^{o}C). Gradient flash chromatography was conducted eluting with a continuous gradient from petroleum to the indicated solvent, and they were listed as volume/volume ratios. NMR spectra were recorded on a Bruker spectrometer at 400 MHz (^{1}H NMR), 101 MHz (^{13}C NMR), 600 MHz (^{1}H NMR), 151 MHz (^{13}C NMR), 376 MHz (^{19}F NMR). Chemical shifts were reported relative to tetramethylsilane, dimethyl sulfoxide (2.50 ppm for ^{1}H, 39.6 ppm for ^{13}C), respectively. And all ^{1}H, ^{13}C and ^{19}F NMR data spectra were reported in delta (δ) units, parts per million (ppm) downfield from the internal standard. Coupling constants were reported in Hertz (Hz). LC-MS spectra were recorded on a AB SCIEX TripleTOF 5600$^{+.}$

Experimental procedure

General procedure for the preparation of 3a:

In an oven-dried undivided three-necked bottle (15 mL) equipped with a stir bar, pyrazolones **1** (0.45 mmol), RSeTs **2** (0.3 mmol), $^{n}Bu_4NBF_4$ (0.3 mmol, 98.7 mg), MeCN (7 mL) was added. The bottle was equipped with graphite rod (ϕ 6 mm, about 15 mm immersion depth in solution) as the anode and platinum plate (15 mm×15 mm×0.3 mm) as the cathode. The reaction mixture was stirred and electrolyzed at a constant current of 12 mA under Ar atmosphere at room temperature for 5 h. After completion of the reaction, as indicated by TLC and LC-MS, the crude mixture product was obtained by flash column chromatography on silica gel (petroleum ether: ethyl acetate = 3: 1).

Procedure for gram scale synthesis of 3a:

In an oven-dried undivided beaker (100 mL) equipped with a stir bar, antipyrine **1a** (7.5 mmol, 1.42 g), PhSeTs **2a** (5.0 mmol, 1.56 g), $^{n}Bu_4NBF_4$ (5.0 mmol, 1.65 g), MeCN (130 mL) were added. The bottle was equipped with graphite rod (ϕ 6 mm, about 15 mm immersion depth in solution) as the anode and platinum plate (15 mm×15 mm×0.3 mm) as the cathode. The reaction mixture was stirred and electrolyzed at a constant current of 12 mA under Ar atmosphere at room temperature for 125 h, after completion of the reaction, as indicated by TLC and LC-MS. The pure product **3a** (yield 83%, white solid, 1.43 g) was obtained by flash column chromatography on silica gel (petroleum ether: ethyl acetate = 3:1).

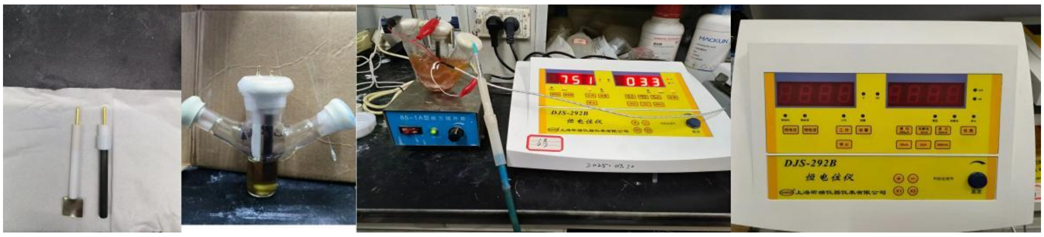

A **B** **C** **D**

Figure **S1**. The experimental setup for electrolysis. (**A:** The electrodes used in the reaction. **B, C** and **D:** The electrochemical reaction apparatus used.)

Anode: graphite rod (Wuhan Gaosiruilian Technology Co., Ltd., ϕ 6 mm, 98% purity, about 15 mm immersion depth in solution, 2.83 cm^2, The length of the carbon rod is about 6 cm.)

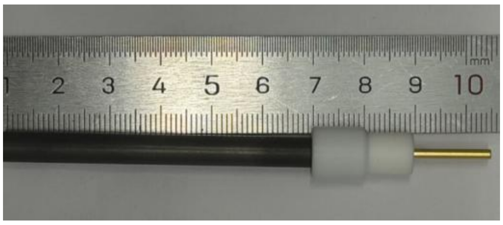

Figure **S2**. Dimensions of the anode

Prior to each reaction, the carbon rod electrode was first gently polished with fine sandpaper to remove surface contaminants. The electrode was then rinsed with deionized water, followed by sequential sonication in deionized water for 5 minutes and in ethanol for 5 minutes, each step repeated three times with fresh solvent, to thoroughly remove any adsorbed impurities. Finally, the electrode was placed in a Schlenk line and dried under vacuum for about 5-10 minutes before use.

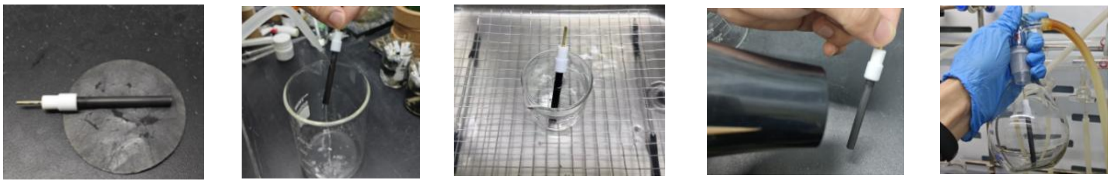

Figure **S3.** Cleaning procedure for the anode

Cathode: (platinum plate (15 mm×15 mm×0.3 mm, pt 1515-3, 99.99% purity, purchased from Wuhan Gaosiruilian Technology Co., Ltd.))

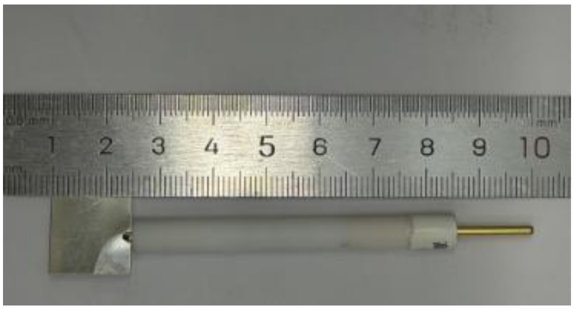

Figure **S4.** Dimensions of the cathode

Prior to each reaction, the electrode surface was first polished with 0.05 μm alumina slurry on a polishing cloth (microcloth) for approximately 2-3 minutes to obtain a mirror-like finish. The electrode was then rinsed with deionized water to remove residual alumina particles, followed by sequential sonication in deionized water for 5 minutes and in ethanol for 5 minutes to remove any adsorbed organic contaminants. Finally, the electrode was placed in a Schlenk line and dried under vacuum for about 5-10 minutes to thoroughly remove any residual solvent from the electrode surface before use.

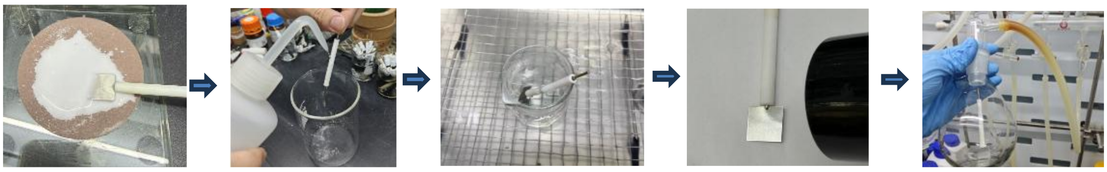

Figure **S5.** Cleaning procedure for the cathode

Procedure for synthesis of 5:

In an oven-dried undivided three-necked bottle (15 mL) equipped with a stir bar, **3a** (0.6 mmol), acids **2** (0.3 mmol), $^{n}Bu_4NOAc$ (0.3 mmol, 98.7 mg), MeCN (9 mL) was added. The bottle was equipped with graphite rod (ϕ 6 mm, about 15 mm immersion depth in solution) as the anode and platinum plate (15 mm×15 mm×0.3 mm) as the cathode. The reaction mixture was stirred and electrolyzed at a constant current of 5 mA under Ar atmosphere at room temperature for 9 h. After completion of the reaction, as indicated by TLC and LC-MS, the crude mixture product was obtained by flash column chromatography on silica gel (petroleum ether: ethyl acetate = 3: 1).

General procedure for the preparation of 1:[1]

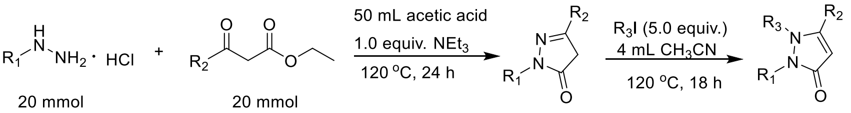

Step1: A mixture of *β*-ketoester (20 mmol) and substituted phenylhydrazine (20 mmol; for the HCl salt, 20 mmol of triethylamine was added) in acetic acid (50 mL) was placed in a 100 mL round-bottom flask equipped with a magnetic stir bar. The reaction mixture was heated under reflux for 24 hours. After completion, the mixture was cooled to room temperature, and the solvent was removed under reduced pressure. The resulting residue was suspended in ethyl acetate and filtered to collect the pure product. Subsequent drying afforded the target substituted pyrazolone.

Step2: A solution of substituted pyrazolone (1.0 equiv.) in acetonitrile (4.0 mL) was treated with methyl iodide or alkyl iodide (5.0 equiv.). The reaction was heated in a sealed tube at the specified temperature for 18 h. Upon completion, as monitored by TLC, the mixture was quenched with saturated aqueous $NaHSO_3$ solution (20 mL) and extracted with dichloromethane (DCM). The combined organic extracts were concentrated under reduced pressure, and the crude residue was purified by silica gel column chromatography (eluent: petroleum ether/ethyl acetate, 1:2, v/v) to afford the desired product **1.**

General procedure for the preparation of 2:[2]

Step1: In a 25 mL schlenk flask was charged with substituted aryl boronic acid (5 mmol, 1.0 equiv.), selenium (15 mmol, 3.0 equiv.) and $AgNO_3$ (10 mol%), then 20 mL of dimethyl sulfoxide was added. Stir the reaction mixture at 120 °C for 12 hours. After completion of the reaction, as indicated by TLC. The contents cooled, dilute the reaction mixture with H_2O (20 mL), the resulting mixture was extracted with EtOAc (3x20 mL). Wash the combined organic phase with water and brine (30 mL). Dry over anhydrous Na_2SO_4. After remove the solvent under reduced pressure, the residue was purified by silica-gel column chromatography to give the products **2** (petroleum ether: ethyl acetate = 1000:1).

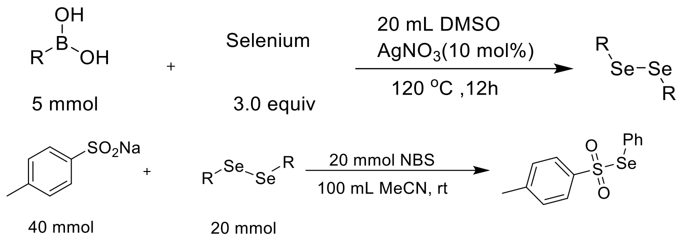

Step2: To a stirred solution of sodium *p*-toluenesulfonate (7.12 g, 40 mmol, 4.0 eq.) and diselenides (3.12 g, 10 mmol, 1.0 eq.) in MeCN (100 mL) was added NBS (3.54 g, 20 mmol, 2.0 eq.) at room temperature. The reaction was detected by TLC until the disulfide was consumed, the reaction quenched with water, and the mixture extracted with ethyl acetate (3x50 mL). The organic layer was dried over anhydrous Na_2SO_4 and filtered. The filtrate was concentrated and the resulting residue was purified by flash chromatography on silica gel (petroleum ether: ethyl acetate = 10:1) to give **2**.

Mechanism research

To gain deeper insights into the reaction mechanism, controlled experiments and cyclic voltammetry analyses were conducted (Scheme 5). Upon addition of 2.0 equiv. of 1,1-diphenylethylene to the reaction mixture, the formation of the DPE-Ts adduct **7a** was confirmed by LC–MS analysis. However, no selenium radical intermediates, such as **8a**, were detected, which is in contrast to our previously reported electrochemical selenylation of pyrazolones[11]. In contrast, the use of PhSeCl under non-electrochemical conditions afforded the desired product in 96% yield, supporting the involvement of $PhSe^+$ as the key intermediate. Furthermore, when using Se-(*p*-tolyl) 4-methylbenzenesulfonoselenoate as the substrate, LC-MS enabled the detection of product **9a**, which arises from sulfonyl radical reduction. These results suggest that sulfonylation likely proceeds via a radical/radical cross-coupling pathway (See

supporting information for details).

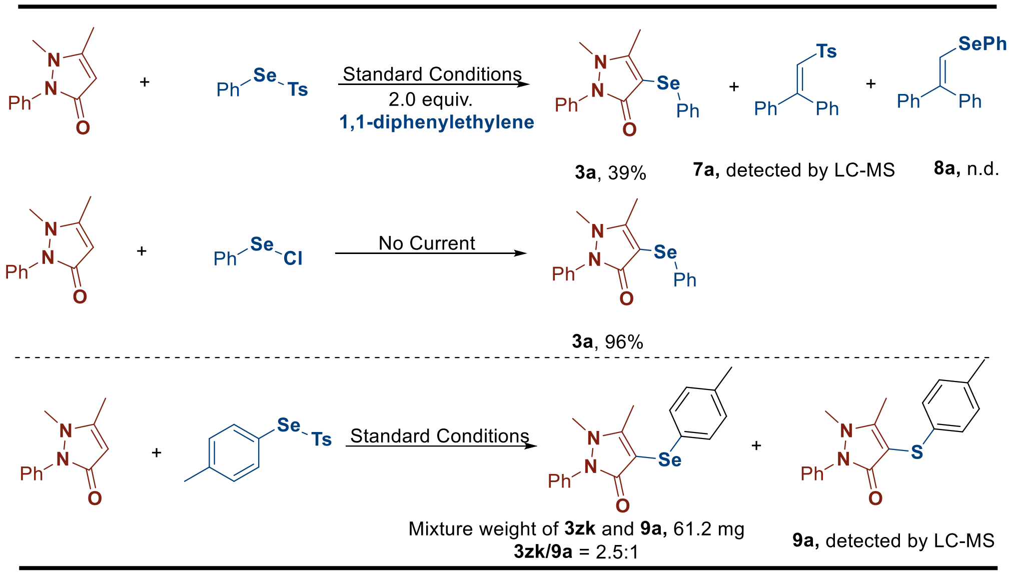

Scheme 1: Controlled experiments

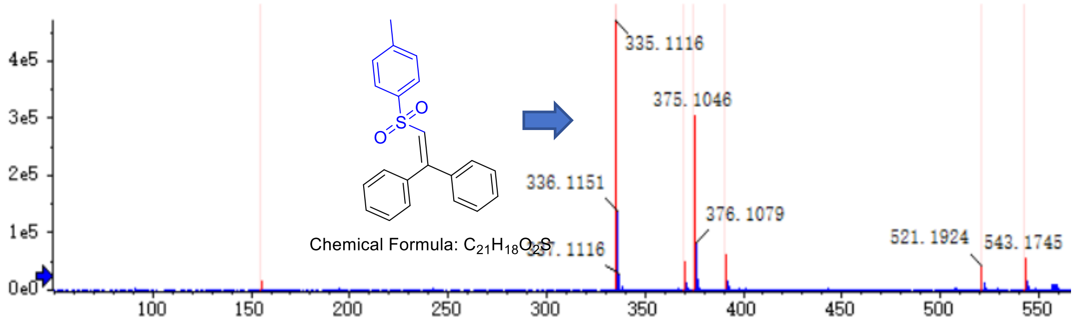

Figure **S6.** Free radical capture experiment (**7a**)

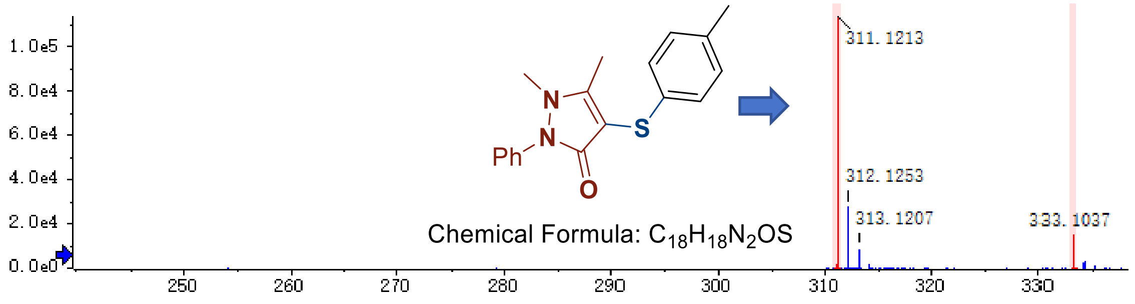

Figure **S7.** Free radical capture experiment (**9a**)

General procedure for cyclic voltammetry (CV):

To gain mechanistic insight, a series of control experiments and cyclic voltammetry (CV) measurements were performed (Figs. S8-S12). All CV experiments were carried out on a CHI760F electrochemical workstation (Shanghai Chenhua Instruments Co., Ltd.) in a three-electrode cell under an argon atmosphere at room temperature. A glassy carbon disk (3 mm diameter, CHI104, Shanghai Chenhua) served as the working electrode, a platinum plate (15 mm

×15 mm×0.3 mm, 99.99% purity, Wuhan Gaosiruilian) as the counter electrode, and a non-aqueous Ag/Ag^{+} reference electrode (0.01 M $AgNO_3$ and 0.1 M $^{n}Bu_4NBF_4$ in MeCN) was used for all measurements;

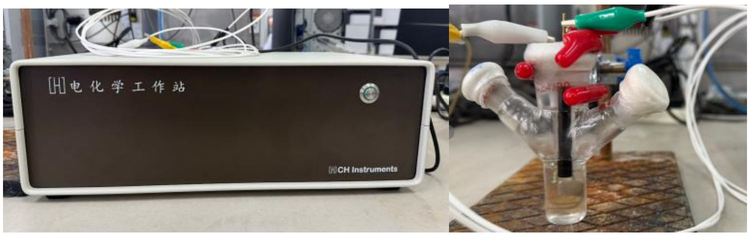

Figure **S8.** electrochemical workstation

1.**Working electrode** (glassy carbon, ϕ 3 mm, CHI104, purchased from Shanghai Chenhua Instruments Co., Ltd. https://www.chinstr.com/)

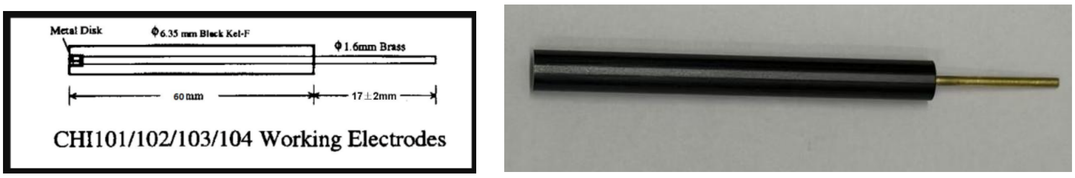

Figure **S9.** Dimensions of the working electrode

Prior to each measurement, the electrode surface was first polished with 0.05 μm alumina slurry on a polishing cloth (microcloth) for approximately 2–3 minutes to obtain a mirror-like finish. The electrode was then rinsed with deionized water to remove residual alumina particles, followed by sequential sonication in deionized water for 5 minutes and in ethanol for 5 minutes to remove any adsorbed organic contaminants. Finally, the electrode was placed in a Schlenk line and dried under vacuum for about 5-10 minutes to thoroughly remove any residual solvent from the electrode surface before use.

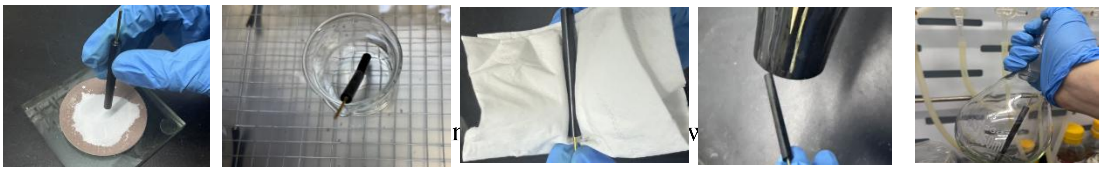

Figure **S10.** Cleaning procedure for the working electrode

2. **Counter electrode** (platinum plate (15 mm × 15 mm × 0.3 mm, pt 1515-3, 99.99% purity, purchased from Wuhan Gaosiruilian Technology Co., Ltd.)), See Figure S4-5;

3.**Non-aqueous reference electrode:** (CHI 112, purchased from Shanghai Chenhua Instruments Co., Ltd.)

To prepare the non-aqueous Ag/Ag^{+} reference electrode filling solution, weigh 13.0 mg of $AgNO_3$ and 230.5 mg of $^{n}Bu_4NBF_4$ with an analytical balance, transfer them to a clean, dry beaker,

and dissolve them in a small portion (about 3-4 mL) of anhydrous acetonitrile with stirring. Once fully dissolved, add additional anhydrous acetonitrile to bring the total volume to exactly 7 mL. For each measurement, the filling solution was freshly prepared prior to use to ensure reproducibility and to avoid any potential contamination or degradation upon storage.

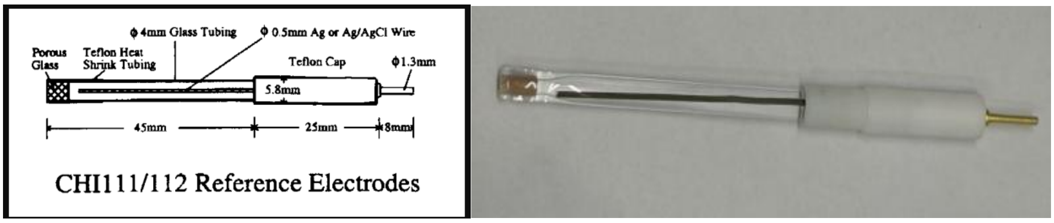

Figure **S11.** Dimensions of the non-aqueous reference electrode

Cyclic voltammetry measurements were performed with the half-wave potential of the ferrocene/ferrocenium (Fc/Fc^{+}) couple measured as +0.096 V versus the non-aqueous Ag/Ag^{+} reference electrode (Fig. S12-A). Under oxidative conditions (Fig. S12-B), **1a** displays an oxidation peak at +0.87 V vs Fc/Fc^{+}, whereas **2a** shows an oxidation peak at +1.67 V vs Fc/Fc^{+}, suggesting that **1a** is more readily oxidized than **2a**. Under reductive conditions (Fig. S12-C, **2a** exhibits reduction peaks at −1.06 V and −1.53 V vs Fc/Fc^{+}, while **1a** shows no well-defined reduction peak within the scanned potential range down to −3.0 V vs Fc/Fc^{+}. This marked difference indicates that **2a** is preferentially reduced at the cathode over **1a**. Therefore, we conclude that **2a** preferentially undergoes reduction rather than oxidation under our reaction conditions

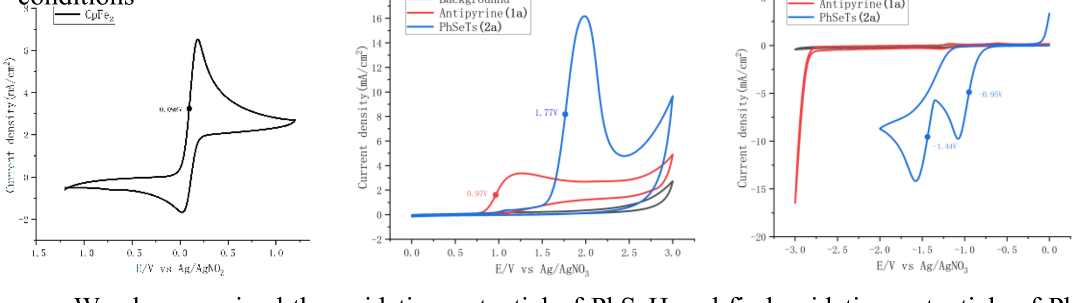

We also examined the oxidation potential of PhSeH and find oxidation potentials of PhSeH were measured at 0.45, 0.89, and 1.62 V (vs. Fc/Fc^{+}), which are significantly lower than those of PhSeTs and antipyrine. This indicates that $PhSe^{-}$ is readily oxidized under our conditions, supporting the proposed mechanism involving $PhSe^{-}$ oxidation.

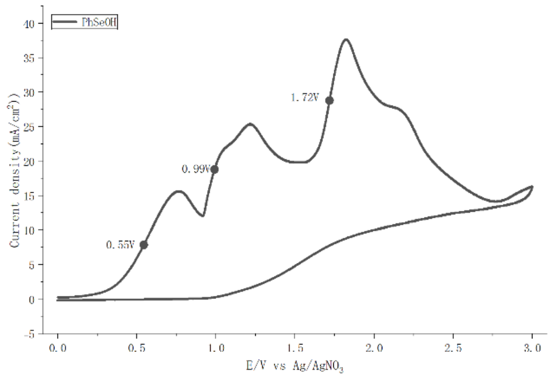

Figure **S13.** Cyclic voltammetry of PhSeH

Detail descriptions for products

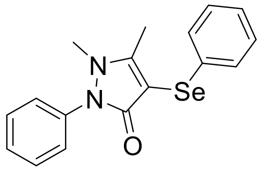

1,5-dimethyl-2-phenyl-4-(phenylselanyl)-1,2-dihydro-3H-pyrazol-3-one (3a).[1] (White solid was obtained in 87% isolated yield, 86.8 mg). ^{1}H NMR (400 MHz, DMSO-d_6) δ 7.52 (t, 2H, J = 8.0 Hz), 7.37 (t, 3H, J = 8.0 Hz), 7.29 (d, 2H, J = 8.0 Hz), 7.25 (t, 2H, J = 8.0 Hz), 7.18 (t, 1H, J = 8.0 Hz), 3.26 (s, 3H), 2.40 (s, 3H) ; ^{13}C NMR (101 MHz, DMSO-d_6) δ 165.52, 160.75, 135.58, 133.00, 129.74, 129.64, 128.89, 127.49, 126.46, 125.29, 91.47, 35.98, 13.28.

HRMS (ESI) calcd for $C_{17}H_{16}N_2OSe$: 345.0501 (M+H$^+$), found: 345.0505.

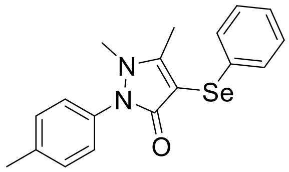

1,5-dimethyl-4-(phenylselanyl)-2-(p-tolyl)-1,2-dihydro-3H-pyrazol-3-one (3b). (White solid was obtained in 88% isolated yield,94.7 mg). ^{1}H NMR (400 MHz, DMSO-d_6) δ 7.33 (d, 2H, J = 8.0 Hz), 7.26 (d, 2H, J = 8.0 Hz), 7.24 (d, 3H, J = 8.0 Hz)，7.18 (t, 1H, J = 8.0 Hz), 3.25 (s, 3H), 2.37 (d, 6H, J = 8.0 Hz); ^{13}C NMR (101 MHz, DMSO-d_6) δ 165.49, 159.89, 137.25, 133.14, 133.00, 130.12, 129.72, 128.83, 126.41, 125.64, 91.11, 35.70, 21.10, 13.20.

HRMS (ESI) calcd for $C_{18}H_{18}N_2OSe$: 359.0657 (M+H$^+$), found: 359.0656.

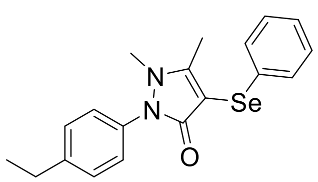

2-(4-ethylphenyl)-1,5-dimethyl-4-(phenylselanyl)-1,2-dihydro-3H-pyrazol-3-one (3c). (White solid was obtained in 83% isolated yield, 93.4 mg). ^{1}H NMR (400 MHz, DMSO-d_6) δ 7.36 (d, 2H, J = 8.0 Hz), 7.29 (t, 4H, J = 8.0 Hz),7.25 (d, 2H, J = 8.0 Hz), 7.18 (t, 1H, J = 8.0 Hz), 3.25 (s, 3H), 2.66 (q, 2H, J = 8.0 Hz), 2.39 (s, 3H), 1.22 (t, 3H, J = 8.0 Hz); ^{13}C NMR (101 MHz, DMSO-d_6) δ 165.50, 159.97, 143.41, 133.19, 133.14, 129.72, 128.96, 128.83, 126.41, 125.63, 91.16, 35.75, 28.23, 15.99, 13.21.

HRMS (ESI) calcd for $C_{19}H_{20}N_2OSe$: 373.0814 (M+Na$^+$), found:373.0814.

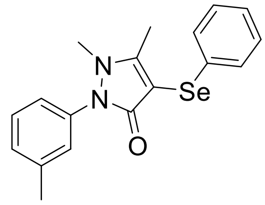

1,5-dimethyl-4-(phenylselanyl)-2-(m-tolyl)-1,2-dihydro-3H-pyrazol-3-one (3d). (White solid was obtained in 77% isolated yield, 83.3 mg). ^{1}H NMR (400 MHz, DMSO-d_6) δ 7.40 (t, 1H, J = 8.0 Hz), 7.29 (d, 2H, J = 8.0 Hz), 7.26 (t, 2H, J = 8.0 Hz), 7.20-7.08 (m, 4H), 3.26 (s, 3H), 2.38 (d, 6H, J = 8.0 Hz); ^{13}C NMR (101 MHz, DMSO-d_6) δ 165.53, 160.53, 139.20, 135.51, 133.05, 129.73, 129.43, 128.90, 128.22, 126.44, 125.84, 122.46, 91.47, 35.93, 21.37, 13.25.

HRMS (ESI) calcd for $C_{18}H_{18}N_2OSe$: 359.0657 (M+Na$^+$), found: 359.0658.

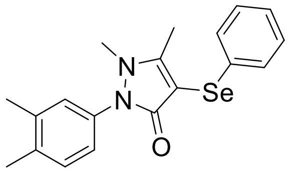

2-(3,4-dimethylphenyl)-1,5-dimethyl-4-(phenylselanyl)-1,2-dihydro-3H-pyrazol-3-one (3e). (White solid was obtained in 87% isolated yield, 97.2 mg). ^{1}H NMR (400 MHz, DMSO-d_6) δ 7.30 (d, 2H, J = 8.0 Hz), 7.27-7.23 (m, 3H), 7.19-7.14 (m, 2H), 7.06 (d, 1H, J = 8.0 Hz), 3.24 (s, 3H), 2.38 (s, 3H), 2.27 (d, J = 4.0 Hz, 6H); ^{13}C NMR (101 MHz, DMSO-d_6) δ 165.50, 159.62, 137.76, 136.13, 133.19, 130.49, 129.70, 128.83, 126.81, 126.39, 123.23, 91.07, 35.65, 19.84, 19.46, 13.17.

HRMS (ESI) calcd for $C_{19}H_{20}N_2OSe$: 373.0814 (M+H$^+$), found:373.0817.

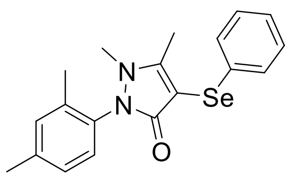

2-(2,4-dimethylphenyl)-1,5-dimethyl-4-(phenylselanyl)-1,2-dihydro-3H-pyrazol-3-one (3f). (White solid was obtained in 92% isolated yield, 103.0 mg). ^{1}H NMR (400 MHz, DMSO-d_6) δ 7.30 (d, 2H,J = 8.0 Hz), 7.26 ((d, 1H,J = 8.0 Hz), 7.23 (d, 1H, J = 8.0 Hz), 7.18 (d, 2H, J = 8.0 Hz), 7.15 (d, 2H, J = 8.0 Hz), 3.17 (s, 3H), 2.36 (d, J = 4.0 Hz, 6H), 2.21 (s, 3H); ^{13}C NMR (101 MHz, DMSO-d_6) δ 164.97, 156.82, 139.44, 137.56, 133.67, 132.01, 131.70, 129.66, 129.26, 128.55, 127.87, 126.26, 89.35, 34.44, 21.17, 17.62, 12.93.

HRMS (ESI) calcd for $C_{19}H_{20}N_2OSe$: 373.0814 (M+H$^+$), found:373.0805.

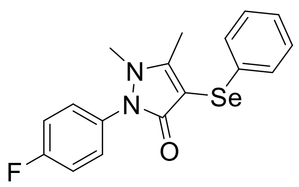

2-(4-fluorophenyl)-1,5-dimethyl-4-(phenylselanyl)-1,2-dihydro-3H-pyrazol-3-one (3g). (White solid was obtained in 55% isolated yield, 59.5 mg). ^{1}H NMR (400 MHz, DMSO-d_6) δ 7.43-7.39 (m, 3H), 7.36 (d, 2H, J = 8.0 Hz), 7.29 (t, 2H, J = 8.0 Hz), 7.25 (d, 1H, J = 8.0 Hz) , 7.18 (t, 1H, J = 8.0 Hz), 3.26 (s, 3H), 2.39 (s, 3H); ^{13}C NMR (101 MHz, DMSO-d_6) δ 165.65, 161.24 (d, J = 245.5 Hz), 160.49, 133.00, 131.87 (d, J = 3.0 Hz), 129.74, 128.86, 127.84 (d, J = 9.0 Hz), 126.45, 116.55 (d, J = 23.2 Hz) , 91.09, 35.79, 13.24. ^{19}F NMR (376 MHz, DMSO-d_6) δ -114.57.

HRMS (ESI) calcd for $C_{17}H_{15}FN_2OSe$: 363.0406 (M+H$^+$), found: 363.0395.

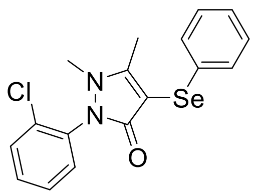

2-(2-chlorophenyl)-1,5-dimethyl-4-(phenylselanyl)-1,2-dihydro-3H-pyrazol-3-one (3h). (White solid was obtained in 77% isolated yield, 87.1 mg). ^{1}H NMR (400 MHz, DMSO-d_6) δ 7.70 (d, 1H, J = 8.0 Hz), 7.60-7.55 (m, 3H), 7.31 (d, 2H, J = 8.0 Hz), 7.26 (t, 1H, J = 8.0 Hz), 7.18 (t, 2H, J = 8.0 Hz), 3.25 (s, 3H), 2.37 (s, 3H); ^{13}C NMR (101 MHz, DMSO-d_6) δ 165.26, 157.73, 133.69, 133.49, 132.91, 132.55, 131.95, 130.93, 129.67, 128.83, 128.54, 126.29, 89.10, 34.57, 12.96.

HRMS (ESI) calcd for $C_{17}H_{15}ClN_2OSe$: 379.0111 (M+H$^+$), found:379.0105.

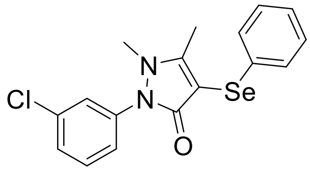

2-(3-chlorophenyl)-1,5-dimethyl-4-(phenylselanyl)-1,2-dihydro-3H-pyrazol-3-one (3i). (White solid was obtained in 76% isolated yield, 86.2 mg). ^{1}H NMR (400 MHz, DMSO-d_6) δ 7.55 (t, 1H, J = 8.0 Hz), 7.48-7.42 (m, 2H), 7.35 -7.24 (m, 6H), 3.29 (s, 3H), 2.41 (s, 3H); ^{13}C NMR (101 MHz, DMSO-d_6) δ 165.63, 162.31, 136.99, 133.87, 132.73, 131.26, 129.76, 129.00, 127.09, 126.55, 124.40, 123.15, 91.84, 36.36, 13.39.

HRMS (ESI) calcd for $C_{17}H_{15}ClN_2OSe$: 379.0111 (M+H$^+$), found:379.0095.

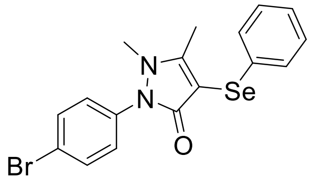

2-(4-bromophenyl)-1,5-dimethyl-4-(phenylselanyl)-1,2-dihydro-3H-pyrazol-3-one (3j).(White solid was obtained in 76% isolated yield, 95.9 mg). ^{1}H NMR (400 MHz, DMSO-d_6) δ 7.72 (d,2H, J = 8.0 Hz), 7.33 (d, 2H, J = 8.0 Hz), 7.29 (t, 2H, J = 8.0 Hz), 7.25 (d, 2H, J = 8.0 Hz), 7.18 (t, 1H, J = 8.0 Hz),3.27 (s, 3H), 2.40 (s, 3H); ^{13}C NMR (101 MHz, DMSO-d_6) δ 165.50, 161.81, 134.94, 132.82, 132.54, 129.75, 128.94, 126.82, 126.52, 119.93, 91.77, 36.21, 13.37.

HRMS (ESI) calcd for $C_{17}H_{15}BrN_2OSe$: 422.9606 (M+Na$^+$), found: 422.9636.

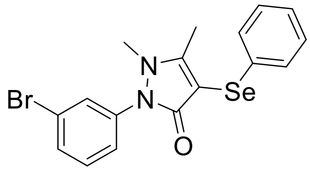

2-(3-bromophenyl)-1,5-dimethyl-4-(phenylselanyl)-1,2-dihydro-3H-pyrazol-3-one (3k). (White solid was obtained in 73% isolated yield, 92.4 mg). ^{1}H NMR (400 MHz, DMSO-d_6) δ 7.60 (s, 1H), 7.56 (d, 1H, J = 8.0 Hz), 7.48 (t, 1H, J = 8.0 Hz), 7.37 (d, 1H, J = 8.0 Hz),7.31 (d, 2H, J = 8.0 Hz), 7.25 (t, 2H, J = 8.0 Hz), 7.18 (t, 1H, J = 8.0 Hz) , 3.28 (s, 3H), 2.40 (s, 3H); ^{13}C NMR (101 MHz, DMSO-d_6) δ 165.63, 162.28, 137.09, 132.74, 131.52, 129.98, 129.76, 129.02, 127.20, 126.55, 123.55, 122.15, 91.84, 36.35, 13.39.

HRMS (ESI) calcd for $C_{17}H_{15}BrN_2OSe$: 422.9606 (M+H$^+$), found: 422.9598.

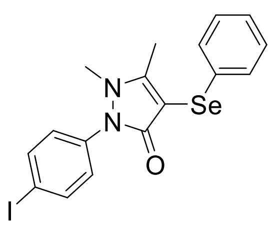

2-(4-iodophenyl)-1,5-dimethyl-4-(phenylselanyl)-1,2-dihydro-3H-pyrazol-3-one (3l). (White solid was obtained in 57% isolated yield, 80.9 mg). ^{1}H NMR (400 MHz, DMSO-d_6) δ 8. 29 (d, 2H, J = 8.0 Hz), 7.72-7.65 (m, 4H), 7.60 (d, 3H, J = 8.0 Hz), 3.69 (s, 3H), 2.82 (s, 3H); ^{13}C NMR (101 MHz, DMSO-d_6) δ 165.43, 161.82, 138.38, 135.40, 132.82, 129.75, 128.93, 126.88, 126.51, 92.62, 91.81, 36.23, 13.37.

HRMS (ESI) calcd for $C_{17}H_{15}IN_2OSe$: 470.9467(M+H$^+$), found:470.9468.

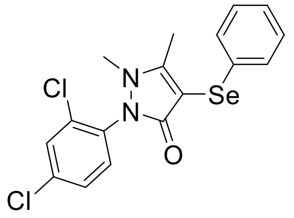

2-(2,4-chlorophenyl)-1,5-dimethyl-4-(phenylselanyl)-1,2-dihydro-3H-pyrazol-3-one (3m). (White solid was obtained in 92% isolated yield, 115.1 mg). ^{1}H NMR (400 MHz, DMSO-d_6) δ 7.90 (s, 1H), 7.64-7.57 (m, 2H), 7.31-7.24 (m, 4H), 7.18 (t, J = 4.0 Hz, 1H),3.23 (s, 3H), 2.37 (s, 3H). ^{13}C NMR (101 MHz, DMSO-d_6) δ 165.30, 158.42, 135.65, 134.82, 133.59, 133.35, 132.11, 130.57, 129.68, 129.03, 128.59, 126.34, 89.20, 34.71, 13.01.

HRMS (ESI) calcd for $C_{17}H_{14}Cl_2N_2OSe$: 412.9721 (M+H$^+$), found:412.9713.

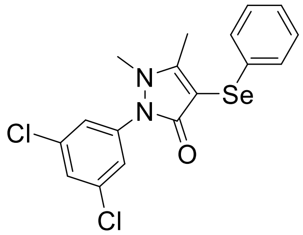

2-(3,5-chlorophenyl)-1,5-dimethyl-4-(phenylselanyl)-1,2-dihydro-3H-pyrazol-3-one (3n). (White solid was obtained in 79% isolated yield, 97.4 mg). ^{1}H NMR (400 MHz, DMSO-d_6) δ 7.59(s, 1H), 7.46 (s, 2H), 7.31-7.17 (m, 5H), 3.30 (s, 3H), 2.40 (s, 3H). ^{13}C NMR (101 MHz, DMSO-d_6) δ 165.73, 163.59, 137.90, 134.87, 132.48, 130.14, 129.79, 129.10, 126.63, 122.70, 92.20, 36.67, 13.47.

HRMS (ESI) calcd for $C_{17}H_{14}Cl_2N_2OSe$: 412.9721 (M+H$^+$), found:412.9720.

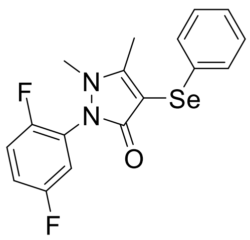

3-**(2,5-difluorophenyl)-1,5-dimethyl-4-(phenylselanyl)-1,2-dihydro-3H-pyrazol-3-one (3o).** (White solid was obtained in 75% isolated yield, 86.0 mg). ^{1}H NMR (400 MHz, DMSO-d_6) δ 7.58-7.41 (m, 4H), 7.30-7.24 (m, 3H), 7.20-7.17 (m, 1H) , 3.29 (s, 3H), 2.38 (s, 3H); ^{13}C NMR (101 MHz, DMSO-d_6) δ 165.66, 159.99, 133.13, 129.98 (d, J = 23.2 Hz) , 129.73, 128.69, 126.53 (d, J = 36.7 Hz) , 126.43, 124.06 (dd, $J1$ = 10.1 Hz , $J2$ = 15.2 Hz) , 118.66-118.02 (m, 1C), 117.64, 117.39, 89.48, 35.07, 13.14.

^{19}F NMR (376 MHz, DMSO-d_6) δ -116.79 (d, J = 15.0 Hz) , -125.18 (d, J = 15.0 Hz).

HRMS (ESI) calcd for $C_{17}H_{14}F_2N_2OSe$:381.0312 (M+H$^+$), found: 381.0316.

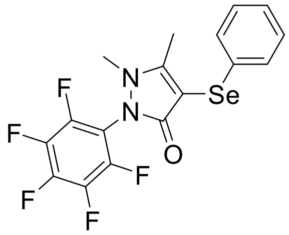

1,5-dimethyl-2-(perfluorophenyl)-4-(phenylselanyl)-1,2-dihydro-3H-pyrazol-3-one (3p). (White solid was obtained in 81% isolated yield, 105.6 mg). ^{1}H NMR (400 MHz, DMSO-d_6) δ 7.28-7.18 (m, 5H) , 3.36 (s, 3H), 2.40 (s, 3H); ^{13}C NMR (101 MHz, DMSO-d_6) δ 165.84, 161.50, 146.46(m, 1C), 143.83(m, 1C), 139.35(m, 1C), 136.85(m, 1C), 132.94, 129.77, 128.62, 126.53, 89.18, 34.90, 13.25.

^{19}F NMR (376 MHz, DMSO-d_6) δ -144.48 (d, J = 18.8 Hz), 150.94(t, J = 22.6 Hz), 161.42(t, J = 22.6 Hz).

HRMS (ESI) calcd for $C_{17}H_{11}F_5N_2OSe$:435.0030 (M+H$^+$), found: 435.0032.

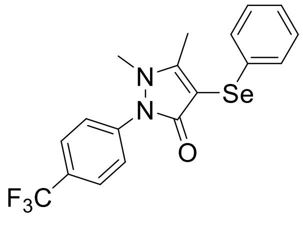

1,5-dimethyl-4-(phenylselanyl)-2-(4-(trifluoromethyl)phenyl)-1,2-dihydro-3H-pyrazol-3-one (3q). (White solid was obtained in 92% isolated yield, 114.4 mg). ^{1}H NMR (400 MHz, DMSO-d_6)

δ 7.89 (d, 2H, J = 8.0 Hz), 7.61 (d, 2H, J = 8.0 Hz), 7.31 (d, 2H, J = 8.0 Hz),7.25 (t, 2H, J = 8.0 Hz), 7.20-7.17 (m, 1H), 3.31 (s, 3H), 2.43 (s, 3H); ^{13}C NMR (101 MHz, DMSO-d_6) δ 165.56, 163.47, 139.12, 132.57, 129.78, 129.05, 126.88 (q, J = 32.3 Hz), 126.75 (q, J = 4.0 Hz), 126.60, 124.57(q, J = 272.7 Hz) , 124.29 , 92.51, 36.72, 13.50.

^{19}F NMR (376 MHz, DMSO-d_6) δ -60.76.

HRMS (ESI) calcd for $C_{18}H_{15}F_3N_2OSe$: 413.0374 (M+H$^+$), found:413.0363.

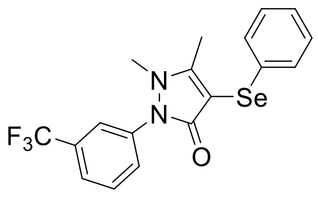

1,5-dimethyl-4-(phenylselanyl)-2-(3-(trifluoromethyl)phenyl)-1,2-dihydro-3H-pyrazol-3-one (3r). (White solid was obtained in 89% isolated yield, 110.2 mg). ^{1}H NMR (400 MHz, DMSO-d_6) δ 7.79-7.71 (m, 3H), 7.67 (d, 1H, J = 8.0 Hz), 7.31 (d, 2H, J = 8.0 Hz),7.26 (t, 2H, J = 8.0 Hz), 7.20-7.17 (m, 1H), 3.30 (s, 3H), 2.43 (s, 3H); ^{13}C NMR (101 MHz, DMSO-d_6) δ 165.67, 162.77, 136.38, 132.64, 130.97, 130.32 (q, J = 32.3 Hz), 129.77, 129.06 , 128.16, 126.58, 124.27 (q, J = 272.7 Hz), 123.63 (q, J = 4.0 Hz), 120.89 (q, J = 4.0 Hz), 92.01, 36.47, 13.43.

^{19}F NMR (376 MHz, DMSO-d_6) δ -61.13.

HRMS (ESI) calcd for $C_{18}H_{15}F_3N_2OSe$: 413.0374 (M+H$^+$), found:413.0369.

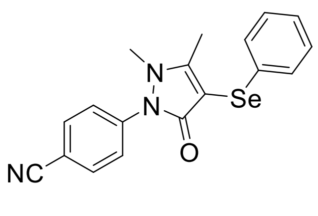

4-(2,3-dimethyl-5-oxo-4-(phenylselanyl)-2,5-dihydro-1H-pyrazol-1-yl)benzonitrile (3s). (White solid was obtained in 72% isolated yield, 80.7 mg). ^{1}H NMR (400 MHz, DMSO-d_6) δ 7.98 (d, 2H, J = 8.0 Hz), 7.58 (d, 2H, J = 8.0 Hz), 7.31 (d, 2H, J = 8.0 Hz), 7.25 (t, 2H, J = 8.0 Hz), 7.18 (t, 1H, J = 8.0 Hz) , 3.30 (s, 3H), 2.42 (s, 3H); ^{13}C NMR (101 MHz, DMSO-d_6) δ 165.45, 164.12, 139.55, 133.80, 132.43, 129.80, 129.11, 126.65, 124.00, 119.01, 108.81, 92.80, 36.94, 13.58.

HRMS (ESI) calcd for $C_{18}H_{15}N_3OSe$: 370.0453 (M+H$^+$), found:370.0452.

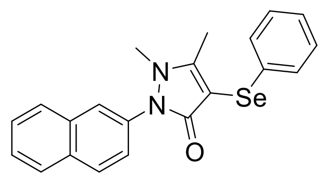

1,5-dimethyl-2-(naphthalen-2-yl)-4-(phenylselanyl)-1,2-dihydro-3H-pyrazol-3-one (3t). (White solid was obtained in 57% isolated yield, 67.1 mg). ^{1}H NMR (400 MHz, DMSO-d_6) δ 8.07-7.98 (m, 3H), 8.00 (s, 1H), 7.61-7.52 (m, 3H), 7.33-7.09 (m, 5H), 3.33 (s, 3H), 2.44 (s, 3H); ^{13}C NMR (101 MHz, DMSO-d_6) δ 165.75, 161.00, 133.40, 133.15, 133.01, 132.01, 129.75, 129.39, 128.92, 128.36, 128.14, 127.32, 126.91, 126.48, 123.76, 123.36, 91.59, 36.17, 13.36.

HRMS (ESI) calcd for $C_{21}H_{18}N_2OSe$: 395.0657 (M+H$^+$), found: 395.0646.

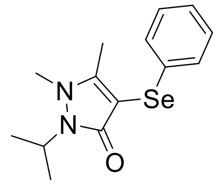

2-isopropyl-1,5-dimethyl-4-(phenylselanyl)-1,2-dihydro-3H-pyrazol-3-one (3u). (White solid was obtained in 58% isolated yield, 53.7 mg).^{1}H NMR (600 MHz, DMSO-d_6) δ 7.24-7.19 (m, 4H), 7.16-7.13 (m, 1H), 4.46-4.42 (m, 1H), 3.41 (s, 3H), 2.26 (s, 3H), 1.36 (d, 6H, J = 6.0 Hz); ^{13}C NMR (151 MHz, DMSO-d_6) δ 167.09, 158.24, 133.62, 129.61, 128.45, 126.16, 48.29, 35.63, 20.52, 13.03.

HRMS (ESI) calcd for $C_{14}H_{18}N_2OSe$: 311.0658 (M+H$^+$), found:311.0661.

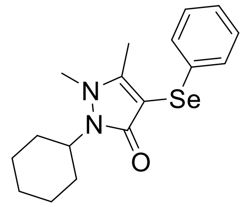

2-cyclohexyl-1,5-dimethyl-4-(phenylselanyl)-1,2-dihydro-3H-pyrazol-3-one (3v). (White solid was obtained in 55% isolated yield, 57.2 mg).^{1}H NMR (600 MHz, DMSO-d_6) δ 7.23-7.18 (m, 4H), 7.16-7.13 (m, 1H), 4.00 (t, 1H, J = 12.0 Hz), 3.41 (s, 3H), 2.25 (s, 3H), 2.03 (q, 2H, J = 12.0 Hz) , 1.78 (d, 2H, J = 12.0 Hz), 1.68 (d, 2H, J = 12.0 Hz), 1.61 (d, 1H, J = 12.0 Hz), 1.32 (q, 2H, J = 12.0 Hz), 1.16 (t, 1H, J = 12.0 Hz); ^{13}C NMR (151 MHz, DMSO-d_6) δ 166.91, 158.14, 133.63, 129.60, 128.46, 126.14, 56.42, 35.63, 30.35, 26.14, 25.38, 13.09.

HRMS (ESI) calcd for $C_{17}H_{22}N_2OSe$: 351.0971 (M+H$^+$), found:351.0964.

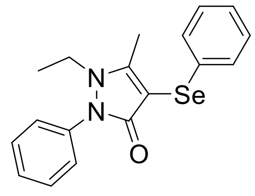

1-ethyl-5-methyl-2-phenyl-4-(phenylselanyl)-1,2-dihydro-3H-pyrazol-3-one (3w). (White solid was obtained in 83% isolated yield, 90.0 mg). ^{1}H NMR (600 MHz, DMSO-d_6) δ 7.53-7.50 (m, 2H), 7.39-7.35 (m, 3H), 7.29-7.23 (m, 4H), 7.18-7.15 (m, 1H), 3.73 (q, 2H, J = 6.0 Hz), 2.40 (s, 3H), 0.87 (t, 3H, J = 6.0 Hz); ^{13}C NMR (151 MHz, DMSO-d_6) δ 165.97, 160.69, 135.53, 132.93, 129.77, 129.68, 128.80, 127.46, 126.47, 125.18, 94.07, 43.15, 13.15, 11.61.

HRMS (ESI) calcd for $C_{18}H_{18}N_2OSe$: 359.0657 (M+H$^+$), found: 359.0650.

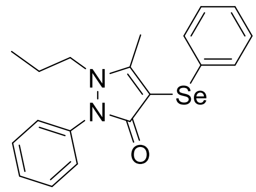

5-methyl-2-phenyl-4-(phenylselanyl)-1-propyl-1,2-dihydro-3H-pyrazol-3-one (3x). (White solid was obtained in 77% isolated yield, 85.2 mg). ^{1}H NMR (400 MHz, DMSO-d_6) δ 7.53 (t, 2H, J = 8.0 Hz), 7.38 (t, 3H, J = 8.0 Hz), 7.28-7.23 (m, 4H), 7.19-7.16 (m, 1H), 3.71 (t, 2H, J = 8.0 Hz), 2.43 (s, 3H), 1.41-1.31 (m, 2H), 0.70 (t, 3H, J = 8.0 Hz); ^{13}C NMR (101 MHz, DMSO-d_6) δ 165.98, 160.21, 135.56, 133.10, 129.75, 129.69, 128.70, 127.66, 126.43, 125.50, 92.01, 49.05, 20.79, 13.33, 11.11.

HRMS (ESI) calcd for $C_{19}H_{20}N_2OSe$: 373.0814 (M+H$^+$), found: 373.0813.

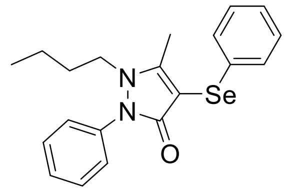

1-butyl-5-methyl-2-phenyl-4-(phenylselanyl)-1,2-dihydro-3H-pyrazol-3-one (3y). (Colorless oil was obtained in 75% isolated yield, 86.7 mg). ^{1}H NMR (400 MHz, DMSO-d_6) δ 7.52 (t, 2H, J = 8.0 Hz), 7.37 (t, 3H, J = 8.0 Hz), 7.28-7.23 (m, 4H), 7.19-7.16 (m, 1H), 3.72 (t, 2H, J = 8.0 Hz), 2.41 (s, 3H), 1.34-1.27 (m, 2H), 1.13-1.04 (m, 2H), 0.73 (t, 3H, J = 8.0 Hz); ^{13}C NMR (101 MHz, DMSO-d_6) δ 165.92, 160.25, 135.54, 133.07, 129.73, 129.68, 128.73, 127.61, 126.44, 125.44, 92.45, 47.44, 29.22, 19.52, 13.92, 13.30.

HRMS (ESI) calcd for $C_{20}H_{22}N_2OSe$: 387.0970 (M+H$^+$), found: 387.0955.

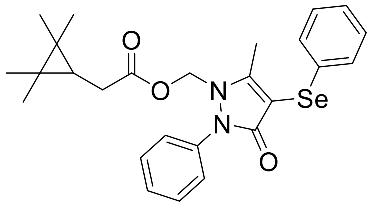

(5-methyl-3-oxo-2-phenyl-4-(phenylselanyl)-2,3-dihydro-1H-pyrazol-1-yl)methyl 2,2,3,3-tetramethylcyclopropane-1-carboxylate (3z). (White solid was obtained in 42% isolated yield, 61.0 mg).^{1}H NMR (600 MHz, DMSO-d_6) δ 7.52-7.49 (m, 2H), 7.37-7.35 (m, 3H), 7.32 (d, 2H, J = 12.0 Hz), 7.28-7.25 (m, 2H), 7.21 (t, 1H, J = 6.0 Hz), 5.67 (s, 2H), 2.48 (s, 3H), 1.18 (s, 1H), 1.10 (d, 12H, J = 24.0 Hz); ^{13}C NMR (151 MHz, DMSO-d_6) δ 170.26, 165.59, 161.73, 135.50, 131.93, 129.83, 129.58, 129.19, 127.47, 126.80, 124.72, 98.31, 67.99, 34.80, 30.88, 23.44, 16.83, 13.21.

HRMS (ESI) calcd for $C_{25}H_{28}N_2O_3Se$: 485.1338 (M+H$^+$), found:485.1337.

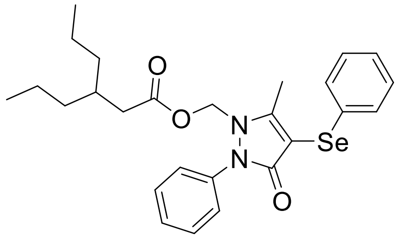

(5-methyl-3-oxo-2-phenyl-4-(phenylselanyl)-2,3-dihydro-1H-pyrazol-1-yl)methyl 2-propylpentanoate (3za). (White solid was obtained in 64% isolated yield, 93.3 mg).^{1}H NMR (600 MHz, DMSO-d_6) δ 7.53-7.50 (m, 2H), 7.37-7.34 (m, 3H), 7.33 (d, 2H, J = 6.0 Hz), 7.27-7.24 (m, 2H), 7.21 (t, 1H, J = 6.0 Hz), 5.73 (s, 2H), 2.50 (s, 3H), 2.30-2.26 (m, 1H), 1.41-1.30 (m, 4H), 1.17-1.11 (m, 4H), 0.79 (t, 6H, J = 6.0 Hz); ^{13}C NMR (151 MHz, DMSO-d_6) δ 174.57, 165.47, 161.91, 135.39, 131.84, 129.75, 129.61, 129.29, 127.38, 126.82, 124.40, 98.69, 68.40, 44.82, 34.35, 20.39, 14.17, 13.18.

HRMS (ESI) calcd for $C_{25}H_{30}N_2O_3Se$: 487.1495 (M+H$^+$), found:487.1487.

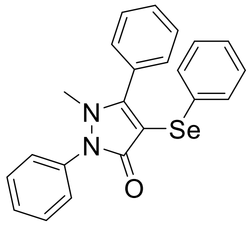

1-methyl-2,5-diphenyl-4-(phenylselanyl)-1,2-dihydro-3H-pyrazol-3-one (3zb). (White solid was obtained in 91% isolated yield, 110.5 mg). ^{1}H NMR (400 MHz, DMSO-d_6) δ 7.64 (s, 2H),

7.57 (d, 7H, J = 8.0 Hz), 7.41-7.38 (m, 1H), 7.31 (d, 2H, J = 8.0 Hz),7.25 (t, 2H, J = 8.0 Hz), 7.19-7.16 (m, 1H), 3.07 (s, 3H). ^{13}C NMR (101 MHz, DMSO-d_6) δ 164.92, 163.00, 135.37, 132.90, 131.25, 130.19, 129.79, 129.67, 129.19, 128.91, 128.88, 127.40, 126.51, 124.77, 93.88, 38.86.

HRMS (ESI) calcd for $C_{22}H_{18}N_2OSe$: 407.0657 (M+H$^+$), found: 407.0647.

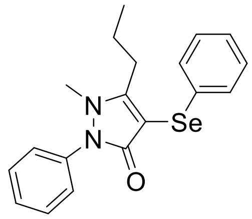

1-methyl-2-phenyl-4-(phenylselanyl)-5-propyl-1,2-dihydro-3H-pyrazol-3-one (3zc). (White solid was obtained in 72% isolated yield, 80.7 mg). ^{1}H NMR (400 MHz, DMSO-d_6) δ 7.53 (t, 2H, J = 8.0 Hz), 7.38 (t, 3H, J = 8.0 Hz), 7.31-7.23 (m, 4H), 7.18 (t, 1H, J = 8.0 Hz), 3.28 (s, 1H), 2.78 (t, 2H, J = 8.0 Hz), 1.59 (q, 2H, J = 8.0 Hz), 0.92 (t, 3H, J = 8.0 Hz); ^{13}C NMR (101 MHz, DMSO-d_6) δ 165.55, 163.67, 135.50, 133.16, 129.69, 129.66, 128.87, 127.56, 126.44, 125.41, 91.55, 35.98, 28.52, 21.87, 14.04.

HRMS (ESI) calcd for $C_{19}H_{20}N_2OSe$: 373.0814 (M+H$^+$), found: 373.0805.

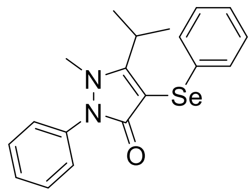

5-isopropyl-1-methyl-2-phenyl-4-(phenylselanyl)-1,2-dihydro-3H-pyrazol-3-one (3zd). (White solid was obtained in 48% isolated yield, 53.8 mg). ^{1}H NMR (400 MHz, $CDCl_3$) δ 7.47-7.44 (m, 3H), 7.41-7.39 (m, 3H), 7.31-7.26 (m, 1H), 7.21-7.12 (m, 3H), 3.43-3.36 (m, 1H) 3.23 (s, 3H), 1.39 (d, 6H, J = 8.0 Hz). ^{13}C NMR (101 MHz, $CDCl_3$) δ 167.81, 166.00, 134.91, 132.49, 130.10, 129.22, 129.06, 126.95, 126.29, 124.55, 94.90, 36.60, 28.15, 20.41.

HRMS (ESI) calcd for $C_{19}H_{20}N_2OSe$: 373.0814 (M+H$^+$), found: 373.0810.

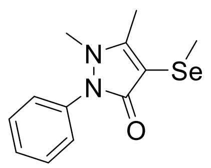

1,5-dimethyl-4-(methylselanyl)-2-phenyl-1,2-dihydro-3H-pyrazol-3-one (3ze). (White solid was obtained in 85% isolated yield, 72.4 mg). ^{1}H NMR (600 MHz, DMSO-d_6) δ 7.49 (t, 2H, J =

6.0 Hz), 7.35-7.30 (m, 3H), 3.13 (s, 3H), 2.36 (s, 3H), 2.06 (s, 3H) ; ^{13}C NMR (151 MHz, DMSO-*d*$_6$) δ 165.33, 159.49, 135.80, 129.50, 126.95, 124.56, 93.97, 36.24, 13.25, 7.44.

HRMS (ESI) calcd for $C_{12}H_{14}N_2OSe$: 283.0344 (M+H$^+$), found:283.0355.

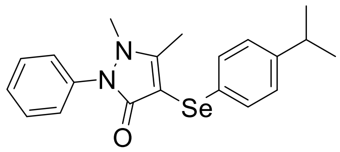

4-((4-isopropylphenyl)selanyl)-1,5-dimethyl-2-phenyl-1,2-dihydro-3H-pyrazol-3-one(3zf).(White solid was obtained in 68% isolated yield, 78.6 mg). ^{1}H NMR (600 MHz, DMSO-*d*$_6$) *δ* 7.51 (t, 2H, *J* = 6.0 Hz), 7.37-7.34 (m, 3H), 7.27 (d, 2H, *J* = 6.0 Hz), 7.13 (d, 2H, *J* = 6.0 Hz), 3.24 (s, 3H), 2.83-2.77 (m, 1H), 2.40 (s, 3H), 1.16 (d, 6H, *J* = 6.0 Hz) ; ^{13}C NMR (151 MHz, DMSO-*d*$_6$) δ 165.57, 160.75, 147.04, 135.74, 129.58, 127.73, 127.37, 126.78, 126.22, 125.17, 92.37, 36.03, 33.43, 24.22, 13.28.

HRMS (ESI) calcd for $C_{20}H_{22}N_2OSe$: 387.0970 (M+H$^+$), found:387.0984.

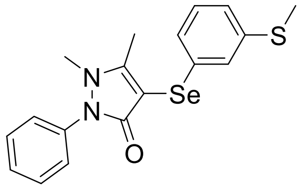

1,5-dimethyl-4-((3-(methylthio)phenyl)selanyl)-2-phenyl-1,2-dihydro-3H-pyrazol-3-one (3zg). (White solid was obtained in 84% isolated yield, 98.8 mg). ^{1}H NMR (600 MHz, DMSO-*d*$_6$) *δ* 7.46 (t, 2H, *J* = 12.0 Hz), 7.38 (d, 2H, *J* = 12.0 Hz), 7.33-7.29 (m, 2H), 7.17 (t, 1H, *J* = 12.0 Hz), 7.11-7.08 (m, 1H), 7.04 (d, 1H, *J* = 12.0 Hz), 3.21 (s, 3H), 2.42 (s, 3H), 2.39 (s, 3H) ; ^{13}C NMR (151 MHz, DMSO-*d*$_6$) δ 165.83, 159.90, 139.44, 135.10, 133.08, 129.29, 127.44, 127.16, 126.68, 124.70, 124.61, 95.42, 35.89, 15.74, 13.25.

HRMS (ESI) calcd for $C_{18}H_{18}N_2OSSe$: 391.0378 (M+H$^+$), found:391.0393.

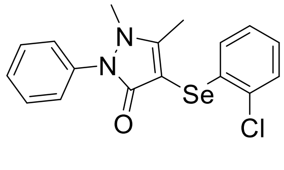

4-((2-chlorophenyl)selanyl)-1,5-dimethyl-2-phenyl-1,2-dihydro-3H-pyrazol-3-one (3zh). (White solid was obtained in 53% isolated yield, 59.6 mg).^{1}H NMR (600 MHz, DMSO-d6) 7.53 (d, 2H, J = 12.0 Hz)，7.43-7.38(m, 4H), 7.23-7.18(m, 2H), 7.01(d, 1H, J = 12.0 Hz), 3.31(s, 3H), 2.37(s, 3H). 13C NMR (151 MHz, DMSO-d6) δ 165.32, 160.82, 135.50, 133.07, 131.58, 129.84,

129.68, 128.76, 128.38, 127.74, 127.64, 125.68, 89.21, 35.87, 13.17.

HRMS (ESI) calcd for $C_{17}H_{16}ClN_2OSe$: 379.0111 (M+H+), found:379.0112.

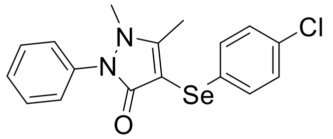

5-((4-chlorophenyl)selanyl)-1,5-dimethyl-2-phenyl-1,2-dihydro-3H-pyrazol-3-one (3zi). (White solid was obtained in 61% isolated yield, 69 mg). ^{1}H NMR (600 MHz, DMSO-d_6) δ 7.54-7.51 (m, 2H), 7.39-7.36 (m, 3H), 7.32 (s, 4H), 3.27 (s, 3H), 2.39 (s, 3H) ; ^{13}C NMR (151 MHz, DMSO-d_6) δ 165.36, 160.52, 135.50, 131.94, 131.30, 130.71, 129.65, 129.58, 127.57, 125.39, 91.14, 35.92, 13.24.

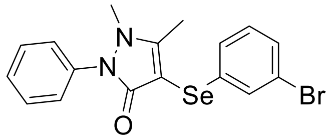

4-((3-bromophenyl)selanyl)-1,5-dimethyl-2-phenyl-1,2-dihydro-3H-pyrazol-3-one (3zj). (White solid was obtained in 35% isolated yield, 44.2 mg). ^{1}H NMR (400 MHz, DMSO-d_6) δ 7.53 (t, 2H, J = 8.0 Hz), 7.46 (s, 1H), 7.37 (t, 4H, J = 8.0 Hz), 7.29 (d, 1H, J = 8.0 Hz), 7.21 (d, 1H, J = 8.0 Hz), 3.28 (s, 3H), 2.40 (s, 3H) ; ^{13}C NMR (101 MHz, DMSO-d_6) δ 165.39, 160.47, 135.85, 135.46, 131.64, 130.78, 129.70, 129.27, 127.75, 127.69, 125.50, 122.92, 90.52, 35.89, 13.26.

HRMS (ESI) calcd for $C_{17}H_{15}BrN_2OSe$: 422.9606 (M+H$^+$), found:422.9617.

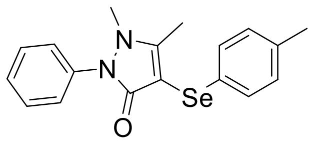

1,5-dimethyl-2-phenyl-4-(p-tolylselanyl)-1,2-dihydro-3H-pyrazol-3-one (3zk). (White solid was obtained in 38% isolated yield, 40.5 mg). ^{1}H NMR (600 MHz, DMSO-d_6) δ 7.53-7.50 (m, 3H), 7.38-7.34 (m, 2H), 7.21 (d, 2H, J = 6.0 Hz), 7.07 (d, 2H, J = 6.0 Hz), 3.24 (s, 3H), 2.39 (s, 3H), 2.23 (s, 3H) ; ^{13}C NMR (151 MHz, DMSO-d_6) δ165.53, 160.71, 135.96, 130.37, 129.76, 129.62, 129.45, 127.38, 126.63, 126.20, 125.14, 36.01, 20.99, 13.29.

HRMS (ESI) calcd for $C_{18}H_{18}N_2OSe$: 359.0658 (M+H$^+$), found:359.0663.

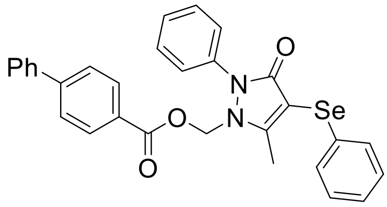

(5-methyl-3-oxo-2-phenyl-4-(phenylselanyl)-2,3-dihydro-1H-pyrrol-1-yl)methyl[1,1'-biphenyl]-4-carboxylate (5a). (White solid was obtained in 46% isolated yield, 75 mg). ^{1}H NMR (400 MHz, DMSO-d_6) δ 7.95 (d, 2H, J = 8.0 Hz), 7.85 (d, 2H, J = 8.0 Hz), 7.76 (d, 2H, J = 8.0 Hz), 7.54 (q, 4H, J = 8.0 Hz), 7.44 (q, 4H, J = 8.0 Hz), 7.27-7.24 (m, 2H), 7.17-7.12 (m, 3H), 5.98 (s, 2H), 2.61 (s, 3H) ; ^{13}C NMR (101 MHz, DMSO) δ 165.52, 164.65, 162.27, 145.98, 139.18, 135.47, 131.94, 130.50, 129.79, 129.75, 129.63, 129.39, 129.06, 128.96, 127.67, 127.54, 126.73, 124.89, 124.42, 99.13, 69.49, 13.35.

HRMS (ESI) calcd for $C_{31}H_{25}NO_3Se$:541.1025 (M+H$^+$), found:541.1022.

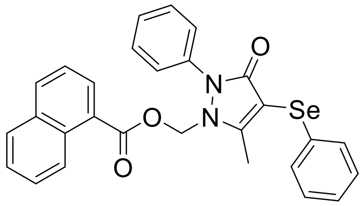

(2-(cyclohexa-2,4-dien-1-yl)-5-methyl-3-oxo-4-(phenylselanyl)-2,3-dihydro-1H-pyrazol-1-yl) methyl 2-naphthoate (5b). (White solid was obtained in 47% isolated yield, 73 mg). ^{1}H NMR (400 MHz, DMSO-d_6) δ 8.62 (d, 1H, J = 8.0 Hz),8.26 (d, 1H, J = 8.0 Hz), 8.08-8.03 (m, 2H), 7.63 (d, 2H, J = 8.0 Hz), 7.56 (d, 1H, J = 8.0 Hz), 7.48 (d, 2H, J = 8.0 Hz),7.42 (t, 1H, J = 8.0 Hz), 7.37-7.27 (m, 2H), 7.22 (d, 2H, J = 8.0 Hz), 7.11 (t, 1H, J = 8.0 Hz), 7.00 (t, 2H, J = 8.0 Hz), 6.07 (s, 2H), 2.63 (s, 3H) ; ^{13}C NMR (101 MHz, DMSO) δ 165.74, 165.54, 162.45, 135.44, 134.71, 133.90, 131.85, 131.30, 130.77, 129.80, 129.68, 129.37, 128.91, 128.70, 127.65, 127.09, 126.64, 125.66, 125.41, 125.25, 124.75, 99.23, 69.57, 13.39.

HRMS (ESI) calcd for $C_{28}H_{24}N_2O_3Se$:515.0869 (M+H$^+$), found:515.0867.

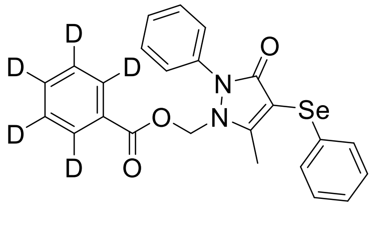

(5-methyl-3-oxo-2-phenyl-4-(phenylselanyl)-2,3-dihydro-1H-pyrazol-1-yl)methylbenzoate-d5 (5c). (White solid was obtained in 54% isolated yield, 76 mg). ^{1}H NMR (400 MHz, DMSO-d_6) δ 7.54 (t, 2H, J = 8.0 Hz), 7.43-7.40 (m, 3H), 7.23 (d, 2H, J = 8.0 Hz), 7.17-7.11 (m, 3H), 5.96 (s, 2H), 2.60 (s, 3H) ; ^{13}C NMR (101 MHz, DMSO) δ 165.51, 164.78, 162.42, 135.44, 131.94, 129.77, 129.74, 129.39, 129.01, 128.84, 128.60, 127.64, 126.70, 124.82, 124.41, 99.15, 69.44, 13.33.

HRMS (ESI) calcd for $C_{24}H_{15}D_5N_2O_3Se$:470.1026 (M+H$^+$), found:470.1020.

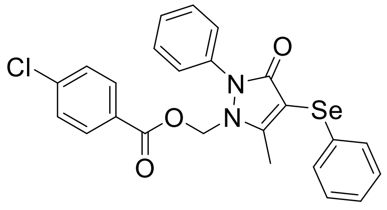

(5-methyl-3-oxo-2-phenyl-4-(phenylselanyl)-2,3-dihydro-1H-pyrazol-1-yl)methyl4-chlorobenzoate (5d). (White solid was obtained in 57% isolated yield, 86 mg). ^{1}H NMR (400 MHz, DMSO-d_6) δ 7.86 (d, 2H, J = 8.0 Hz), 7.63 (d, 2H, J = 8.0 Hz), 7.53 (t, 2H, J = 8.0 Hz), 7.40 (d, 4H, J = 8.0 Hz), 7.23 (d, 2H, J = 8.0 Hz), 7.16 (d, 2H, J = 8.0 Hz), 5.95 (s, 2H), 2.56 (s, 3H) ; ^{13}C NMR (101 MHz, DMSO) δ 165.51, 164.03, 162.20, 139.55, 135.44, 131.66, 129.78, 129.74, 129.68, 129.39, 128.96, 127.62, 126.76, 124.92, 124.41, 99.02, 69.64, 13.32.

HRMS (ESI) calcd for $C_{24}H_{19}ClN_2O_3Se$:499.0323 (M+H$^+$), found:499.0220.

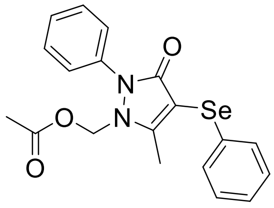

(5-methyl-3-oxo-2-phenyl-4-(phenylselanyl)-2,3-dihydro-1H-pyrazol-1-yl)methyl acetate (5e). (White solid was obtained in 45% isolated yield, 54 mg). ^{1}H NMR (600 MHz, DMSO-d_6) δ 7.51 (t, 2H, J = 6.0 Hz), 7.39-7.37 (m, 3H), 7.30-7.26 (m, 4H), 7.20 (t, 1H, J = 6.0 Hz), 5.66 (s, 2H), 2.48 (s, 3H), 1.98 (s, 3H) ; ^{13}C NMR (151 MHz, DMSO) δ 169.79, 165.57, 161.91, 135.40, 131.97, 129.88, 129.67, 129.08, 127.62, 126.78, 124.90, 98.55, 68.80, 20.67, 13.18.

HRMS (ESI) calcd for $C_{19}H_{18}N_2O_3Se$:403.0556 (M+H$^+$), found:403.0542.

References

(1) Thupyai, A.; Pimpasri, C.; Yotphan, S., DABCO-catalyzed silver-promoted direct thiolation of pyrazolones with diaryl disulfides. *Org. Biomol. Chem.*, **2018**, *16*, 424-432.

(2) Ma, Y., Lin, C., Huang, X., Liu, M., Zhou,Y., Wu H., An $(NH_4)_2S_2O_8$-promoted cross-coupling of thiols/diselenides and sulfoxides for the synthesis of unsymmetrical disulfides/selenosulfides, *Chem. Commun.*, **2022**, *58*, 6550-6553.

Copies of ^{1}H NMR, ^{13}C NMR and ^{19}F NMR spectra

^{1}H NMR (400 MHz, DMSO-d_6) of compound **3a**

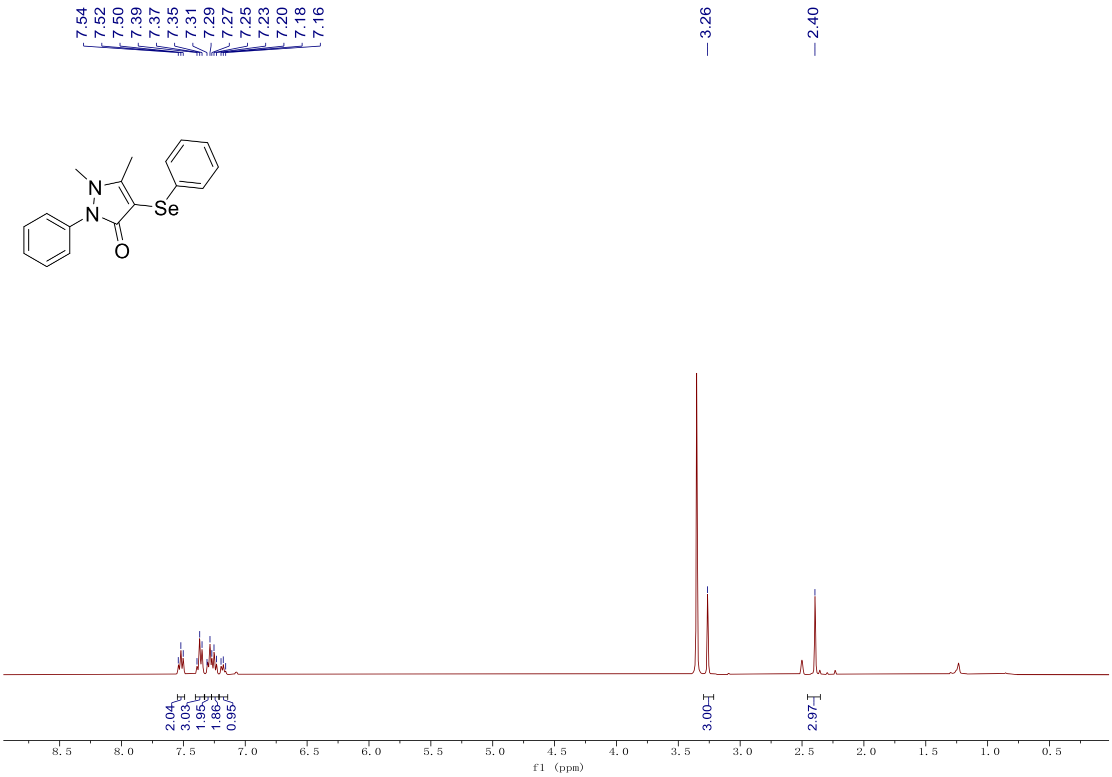

^{13}C NMR (101 MHz, DMSO-d_6) of compound **3a**

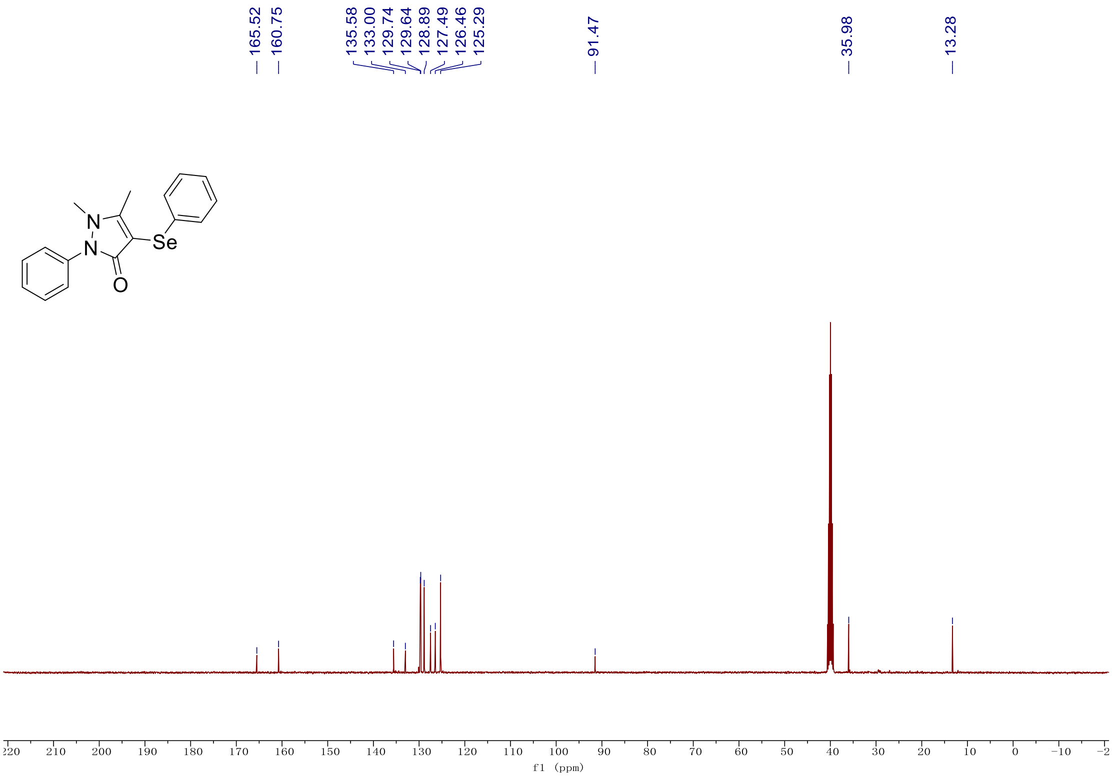

^{1}H NMR (400 MHz, DMSO-d_6) of compound **3b**

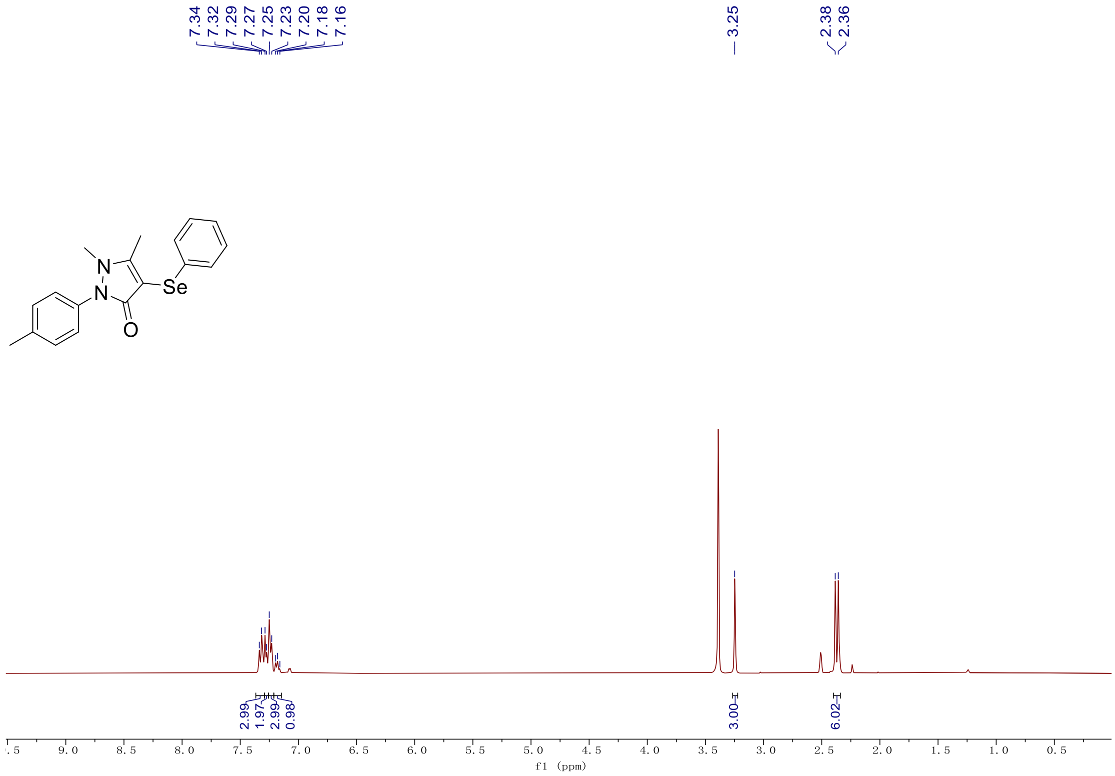

^{13}C NMR (101 MHz, DMSO-d_6) of compound **3b**

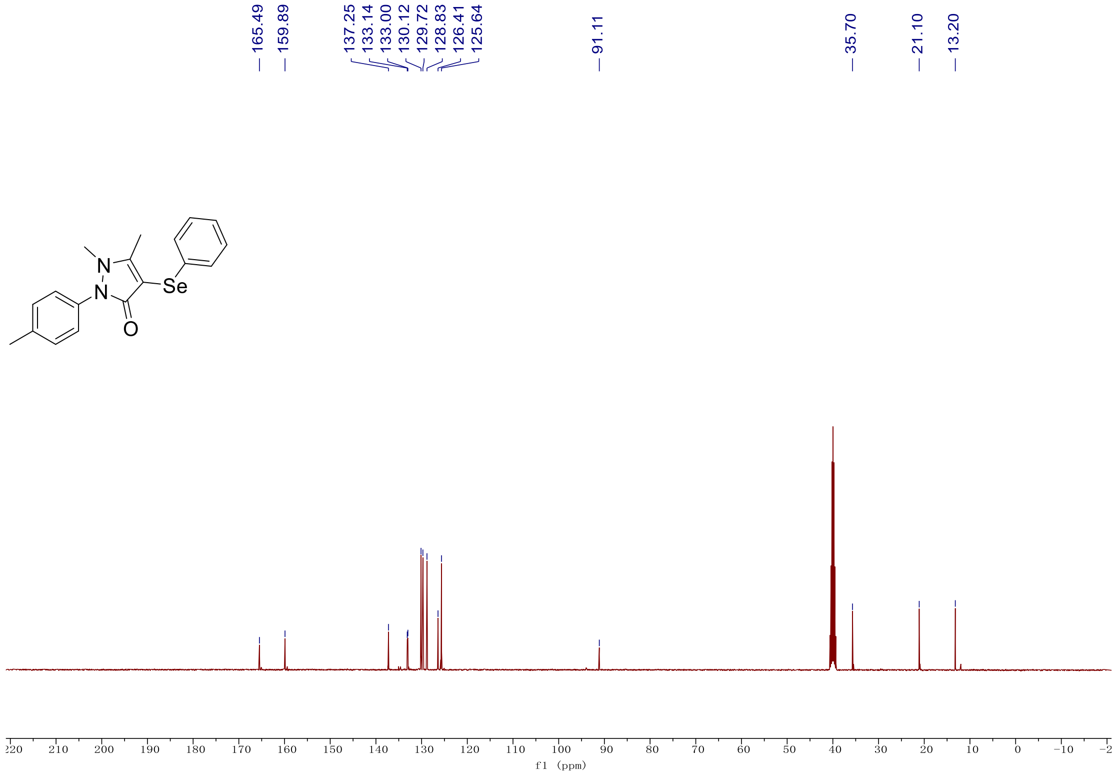

^{1}H NMR (400 MHz, DMSO-d_6) of compound **3c**

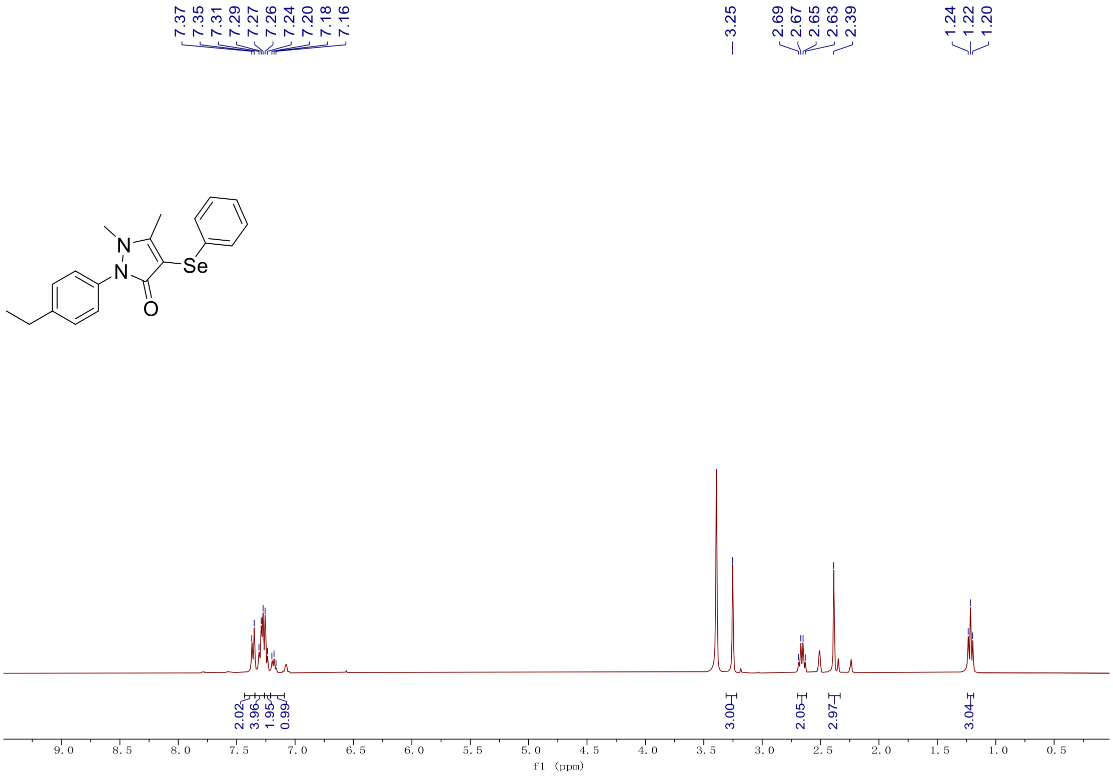

^{13}C NMR (101 MHz, DMSO-d_6) of compound **3c**

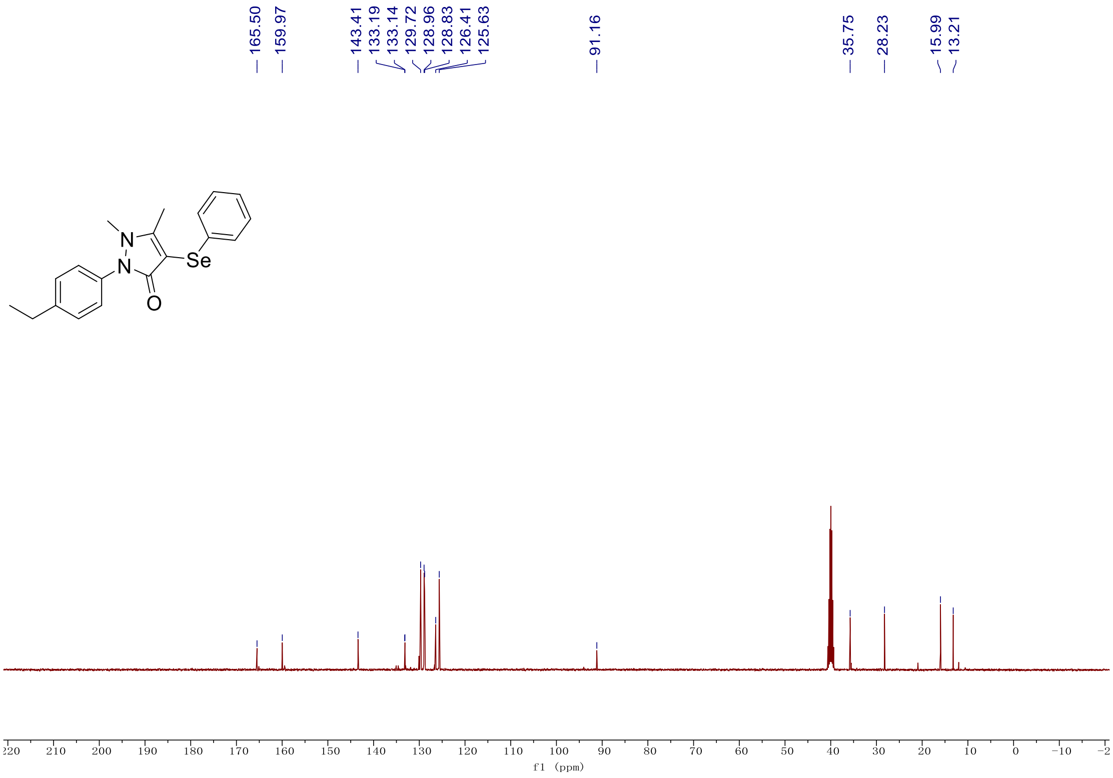

^{1}H NMR (400 MHz, DMSO-d_6) of compound **3d**

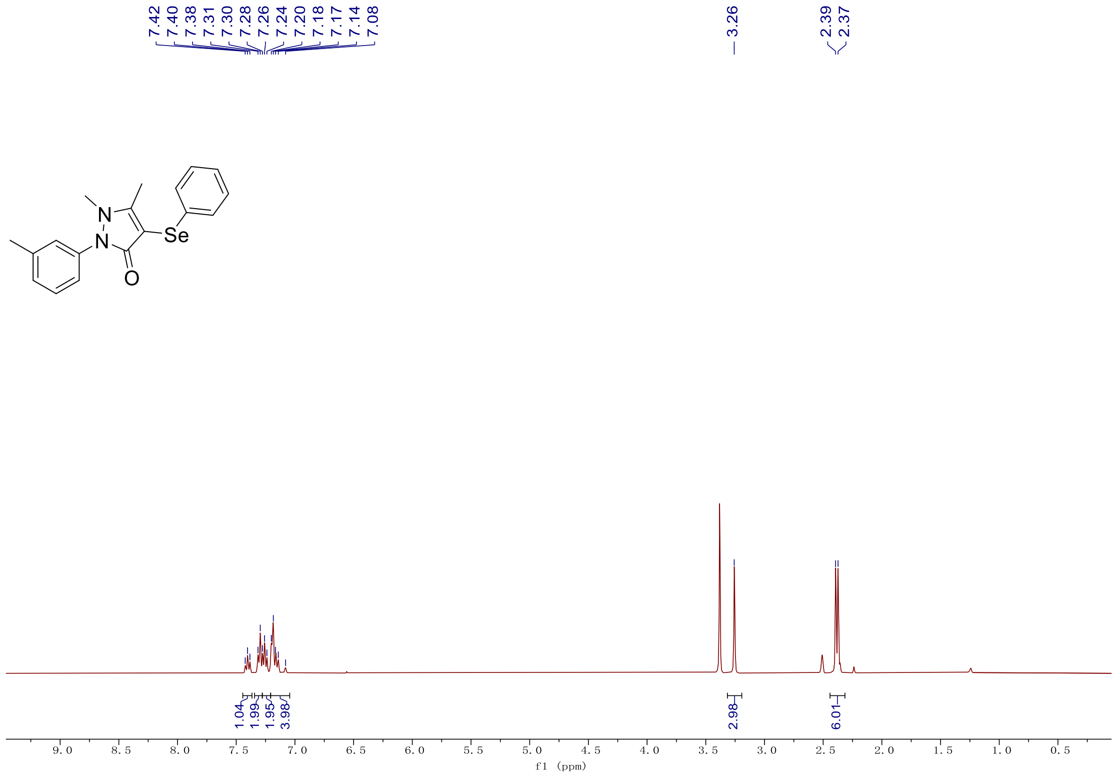

^{13}C NMR (101 MHz, DMSO-d_6) of compound **3d**

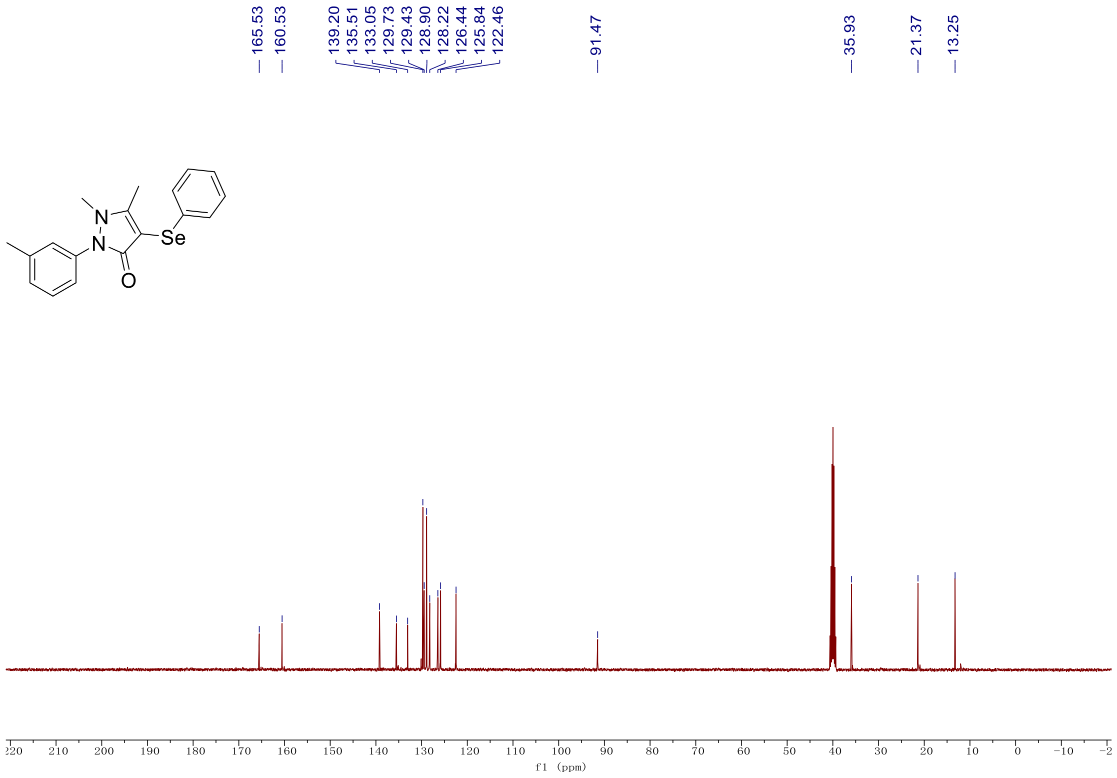

^{1}H NMR (400 MHz, DMSO-d_6) of compound **3e**

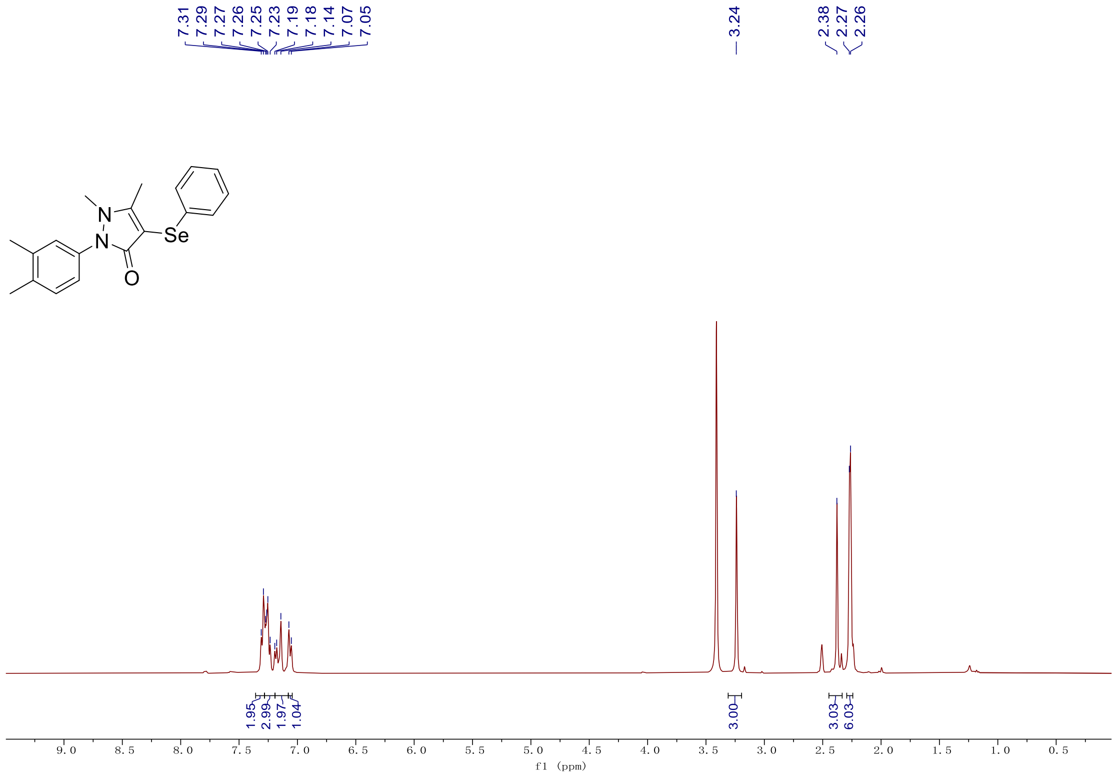

^{13}C NMR (101 MHz, DMSO-d_6) of compound **3e**

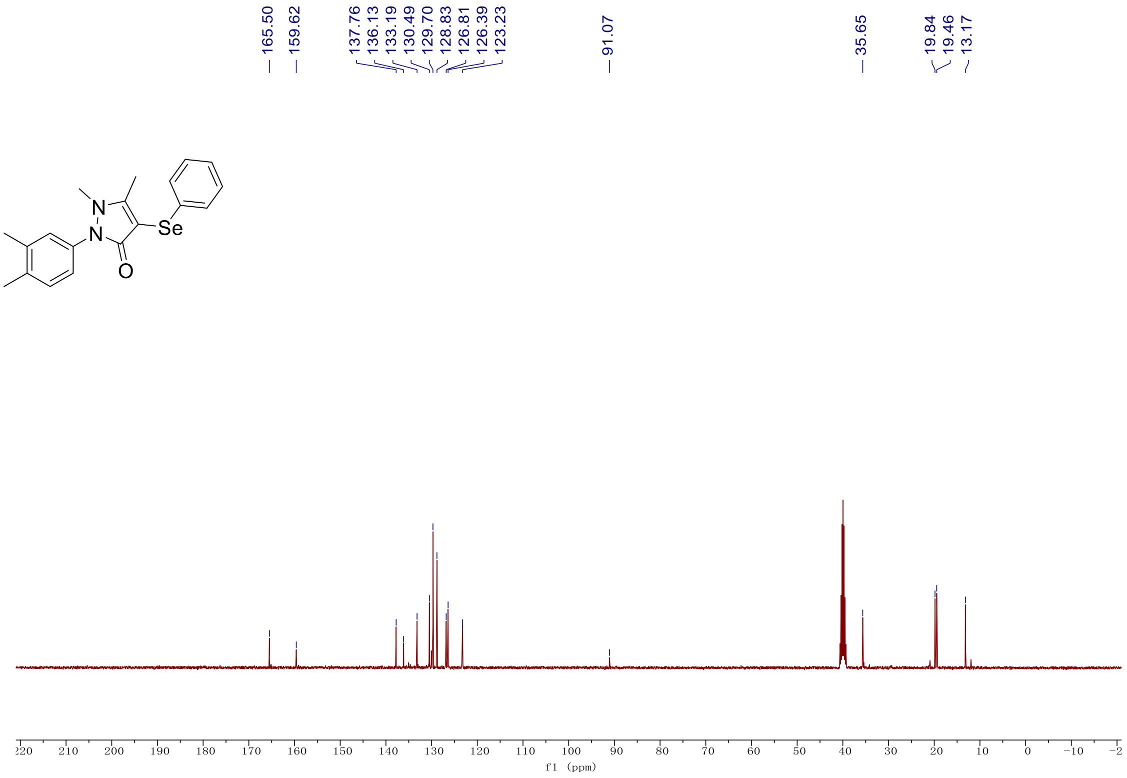

^{1}H NMR (400 MHz, DMSO-d_6) of compound **3f**

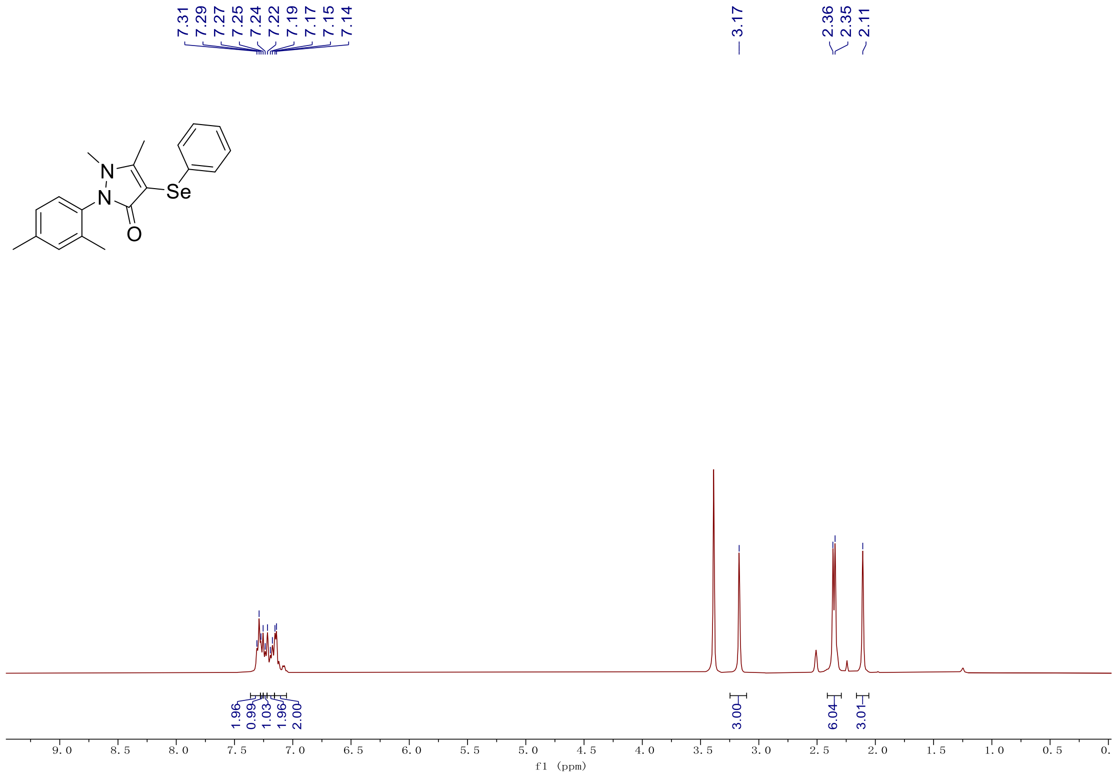

^{13}C NMR (101 MHz, DMSO-d_6) of compound **3f**

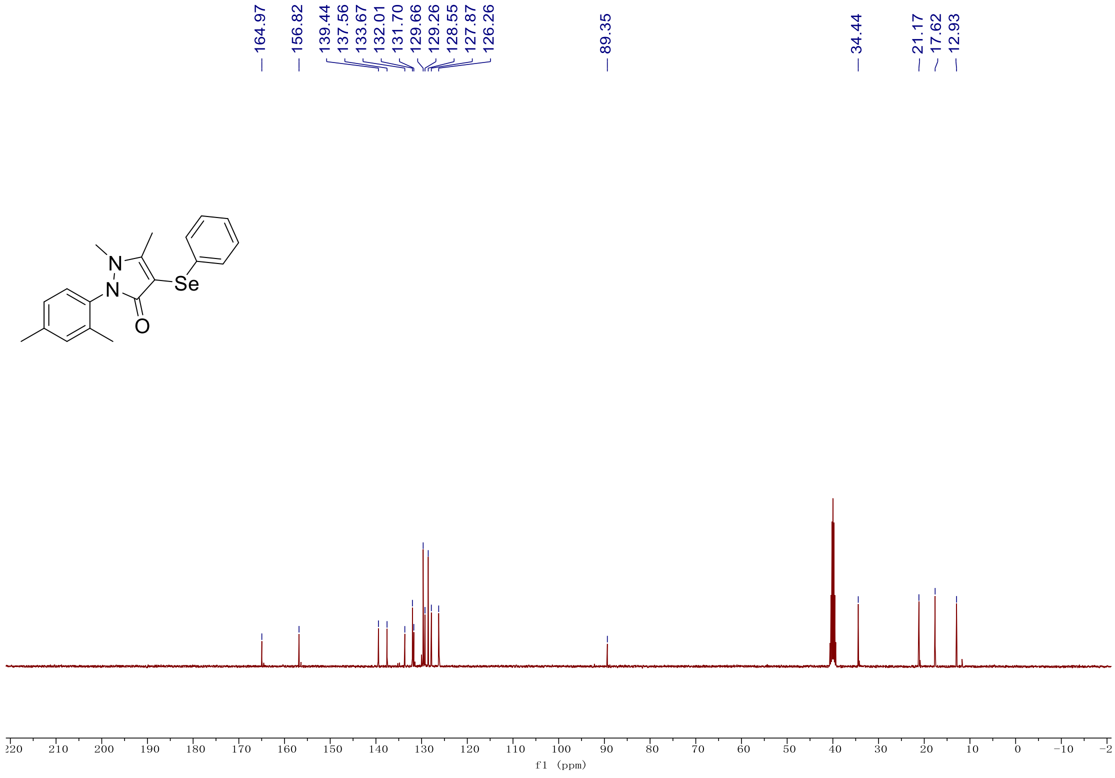

^{1}H NMR (400 MHz, DMSO-d_6) of compound **3g**

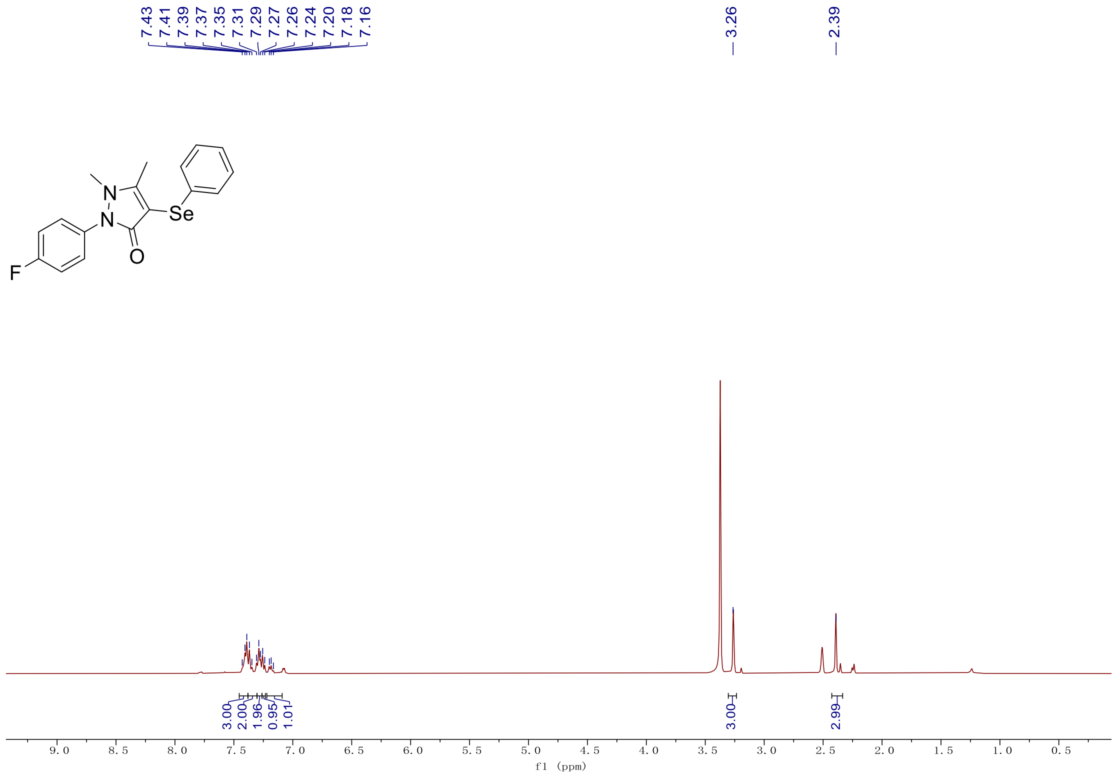

^{13}C NMR (400 MHz, DMSO-d_6) of compound **3g**

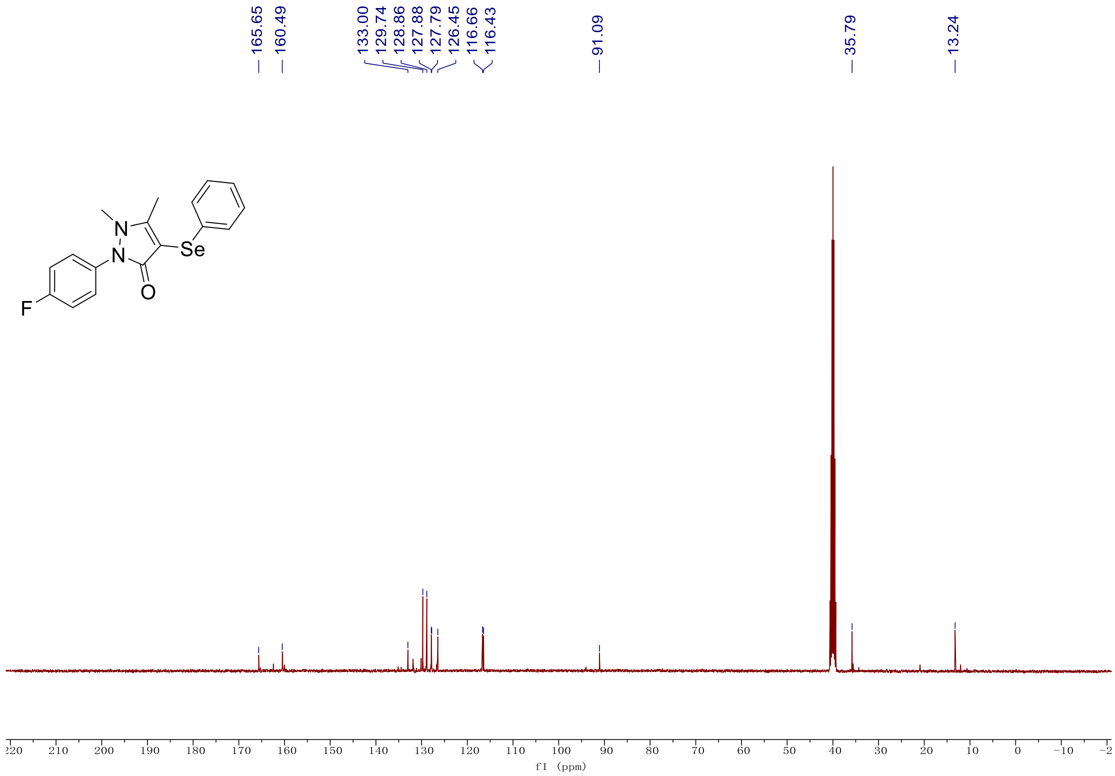

^{19}F NMR (376 MHz, DMSO-d_6) of compound **3g**

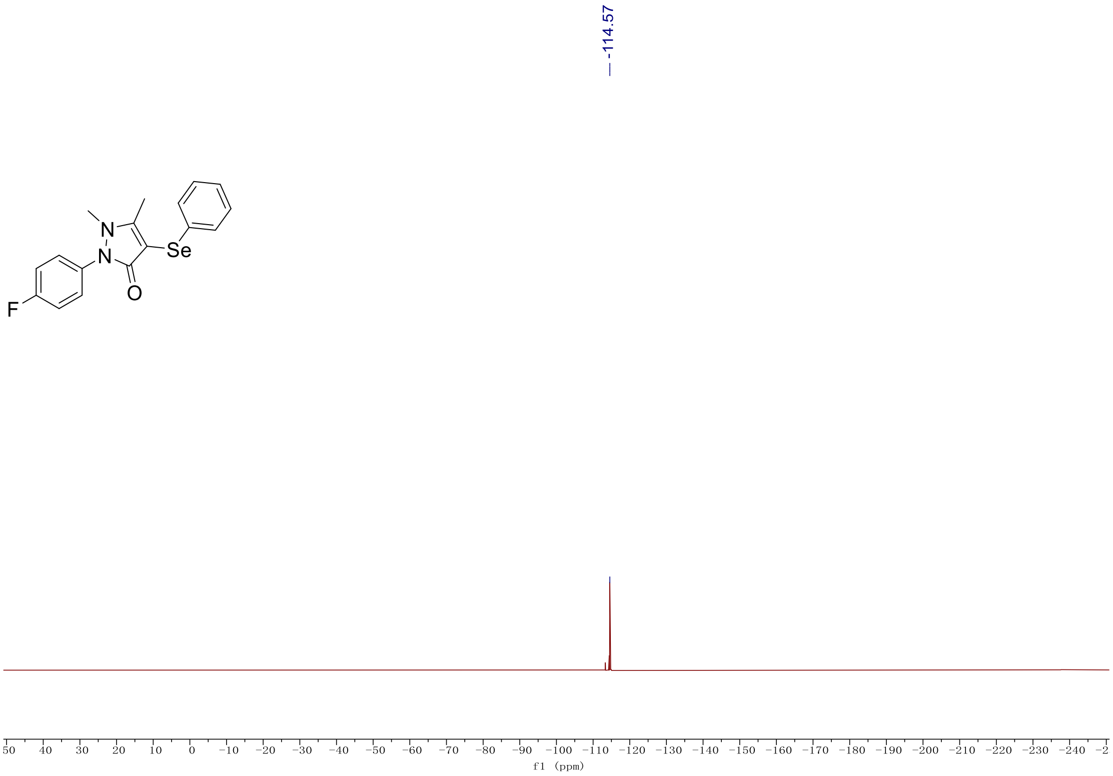

^{1}H NMR (400 MHz, DMSO-d_6) of compound **3h**

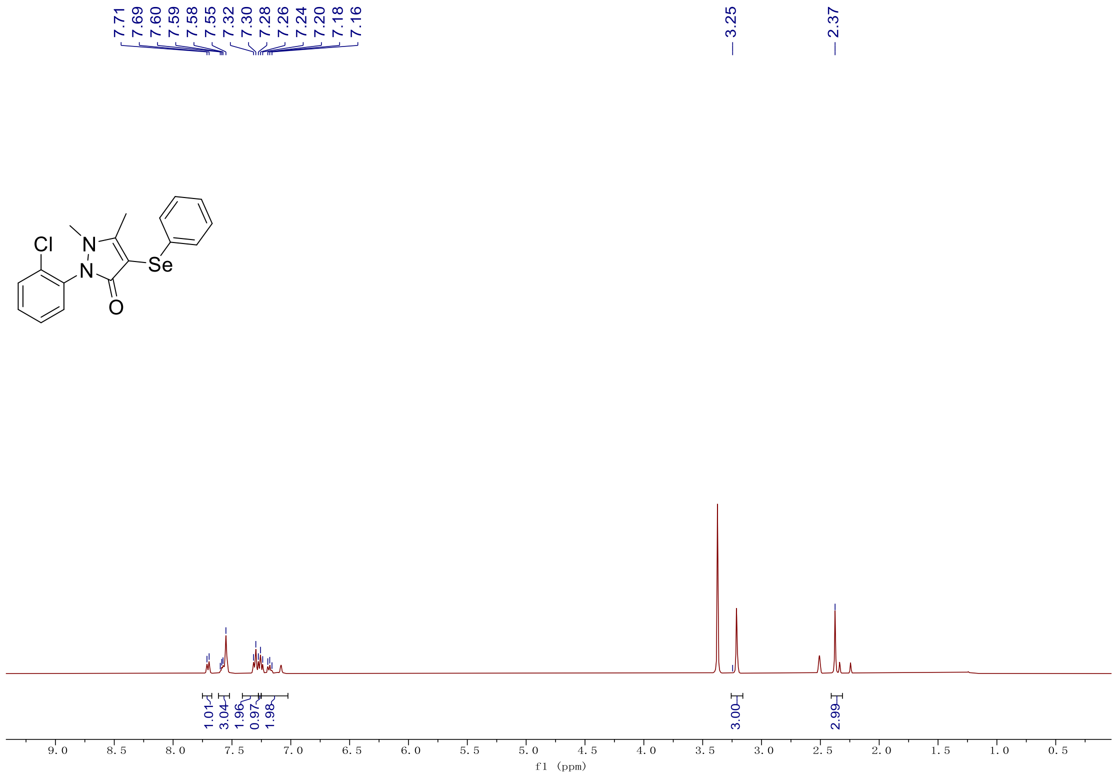

^{13}C NMR (101 MHz, DMSO-d_6) of compound **3h**

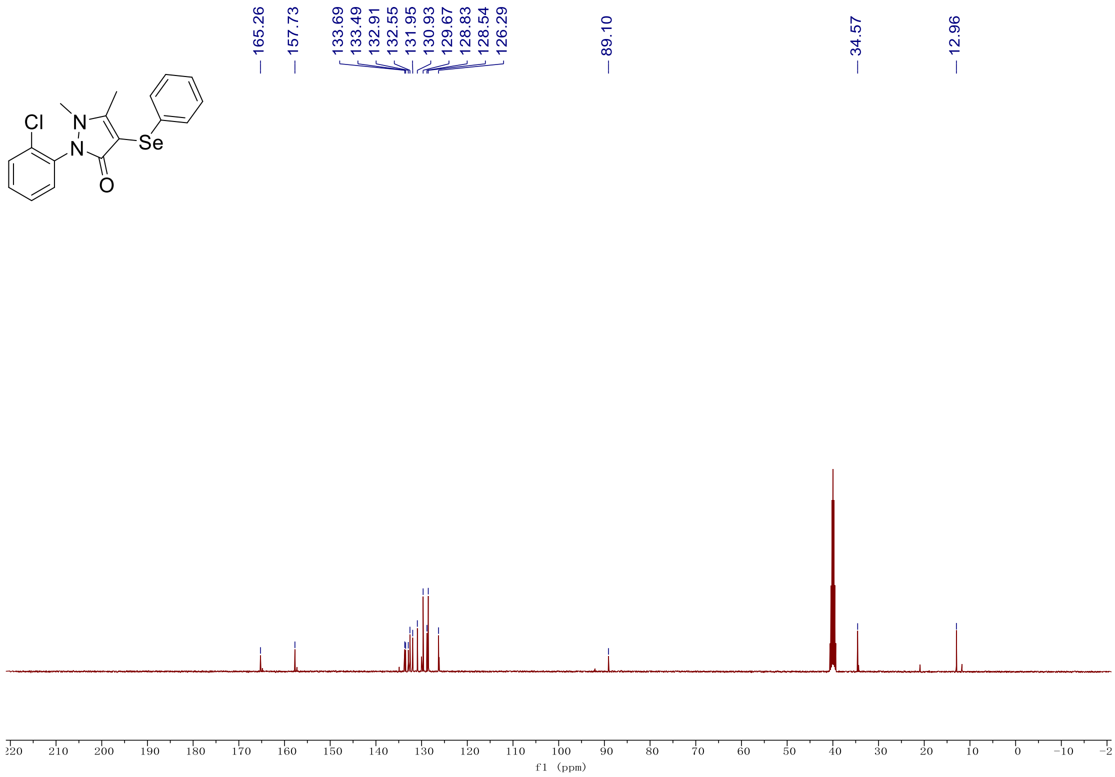

^{1}H NMR (400 MHz, DMSO-d_6) of compound **3i**

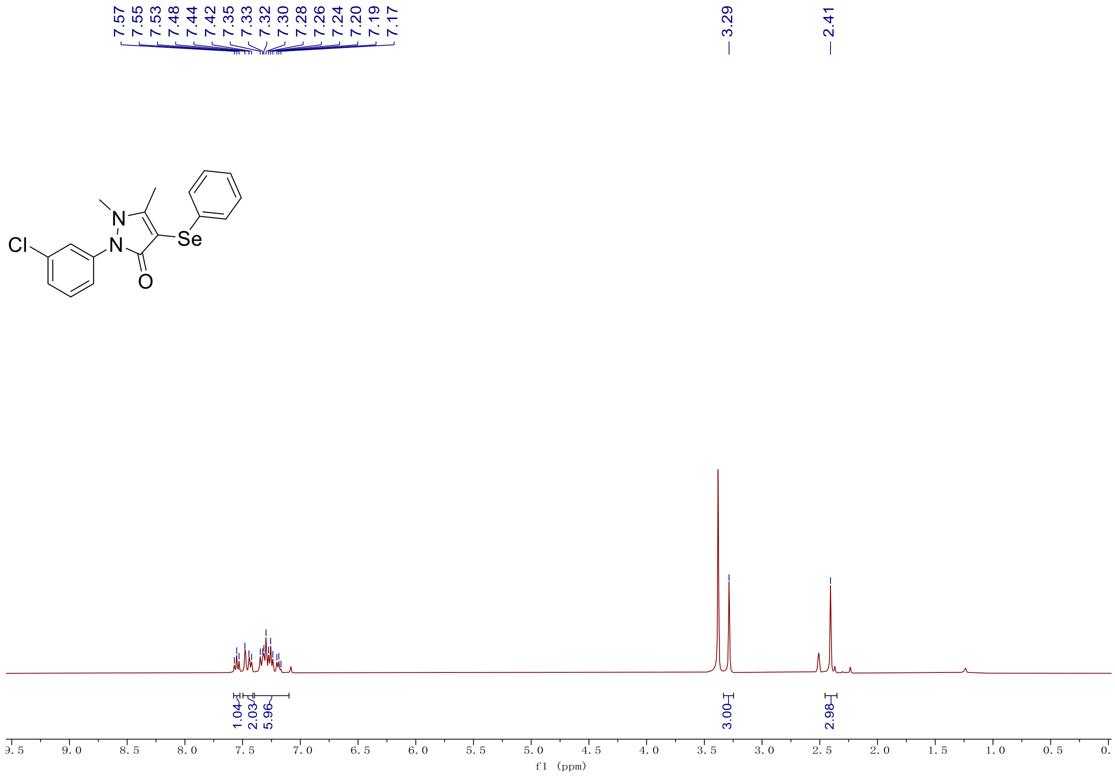

^{13}C NMR (101 MHz, DMSO-d_6) of compound **3i**

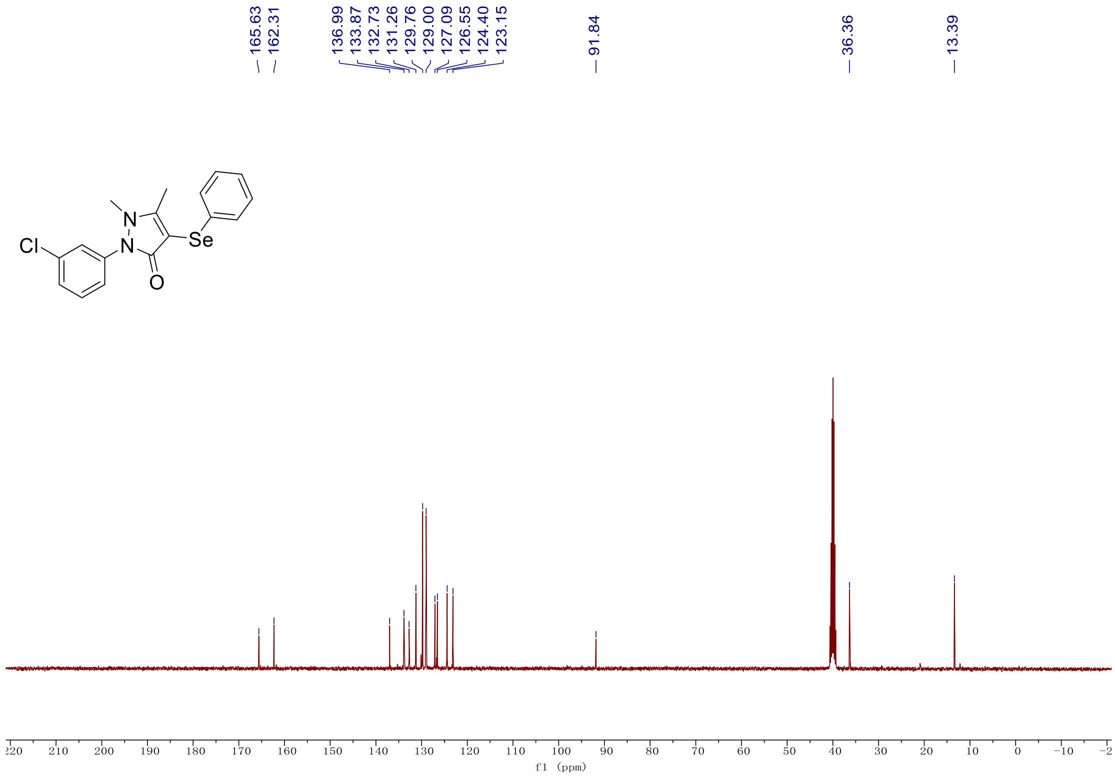

^{1}H NMR (400 MHz, DMSO-d_6) of compound **3j**

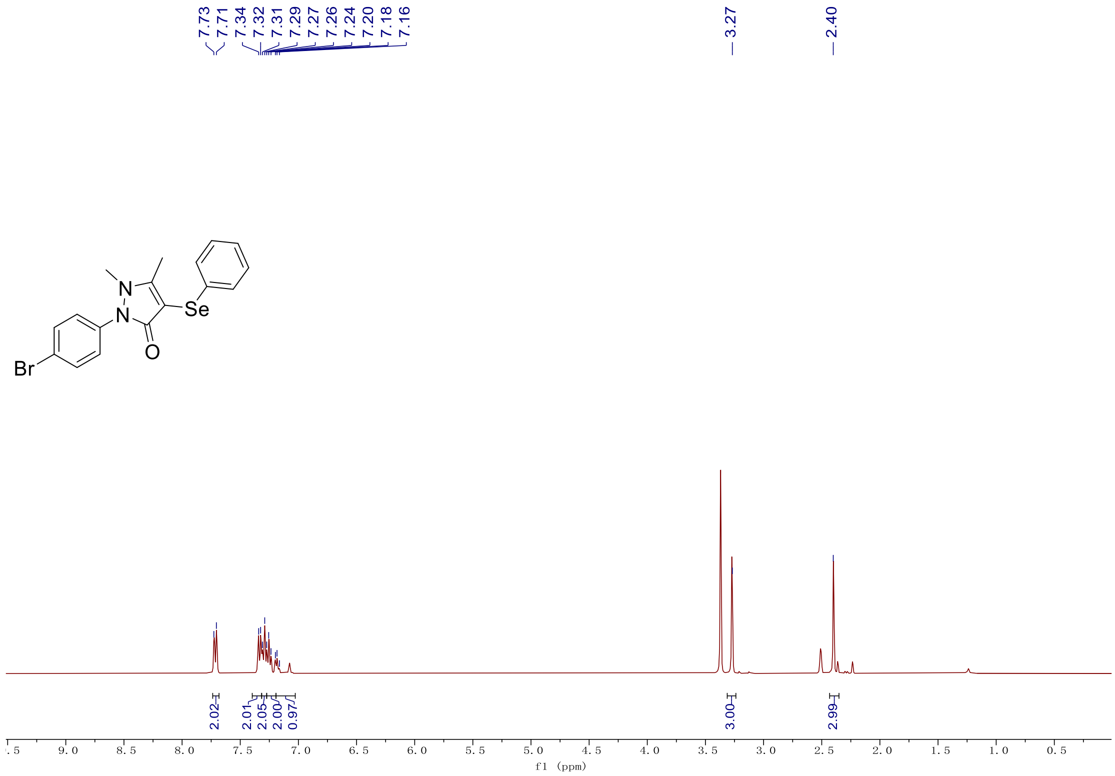

^{13}C NMR (101 MHz, DMSO-d_6) of compound **3j**

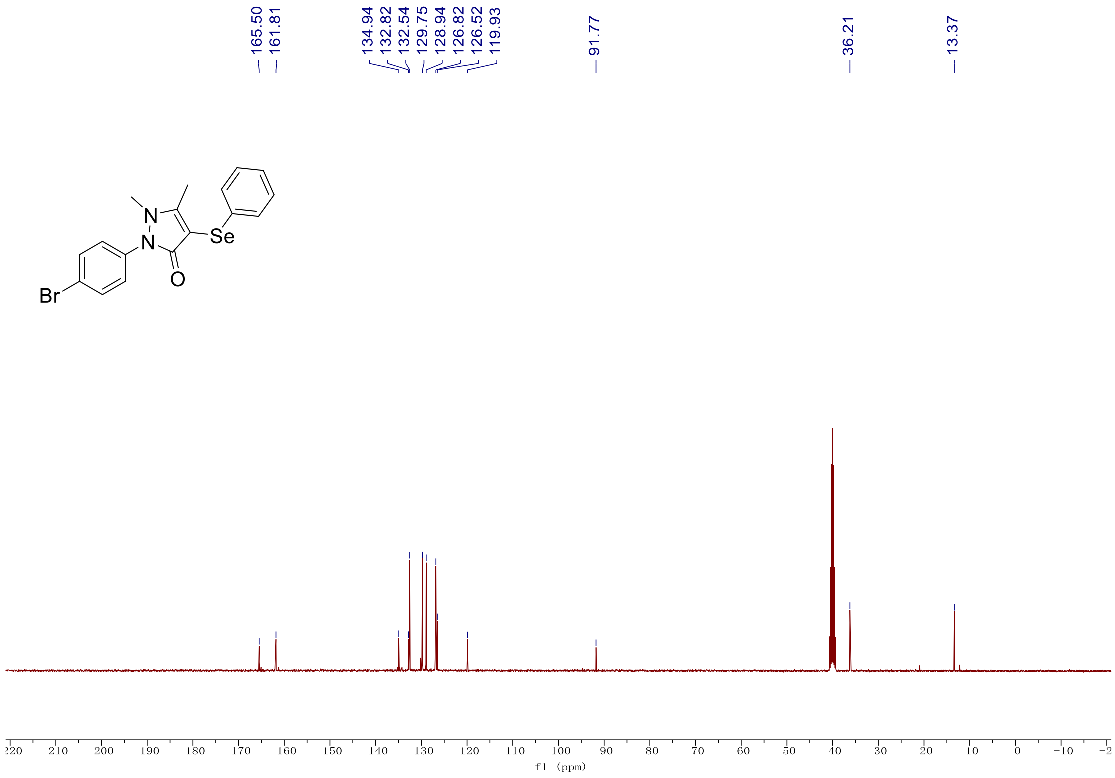

^{1}H NMR (400 MHz, DMSO-d_6) of compound **3k**

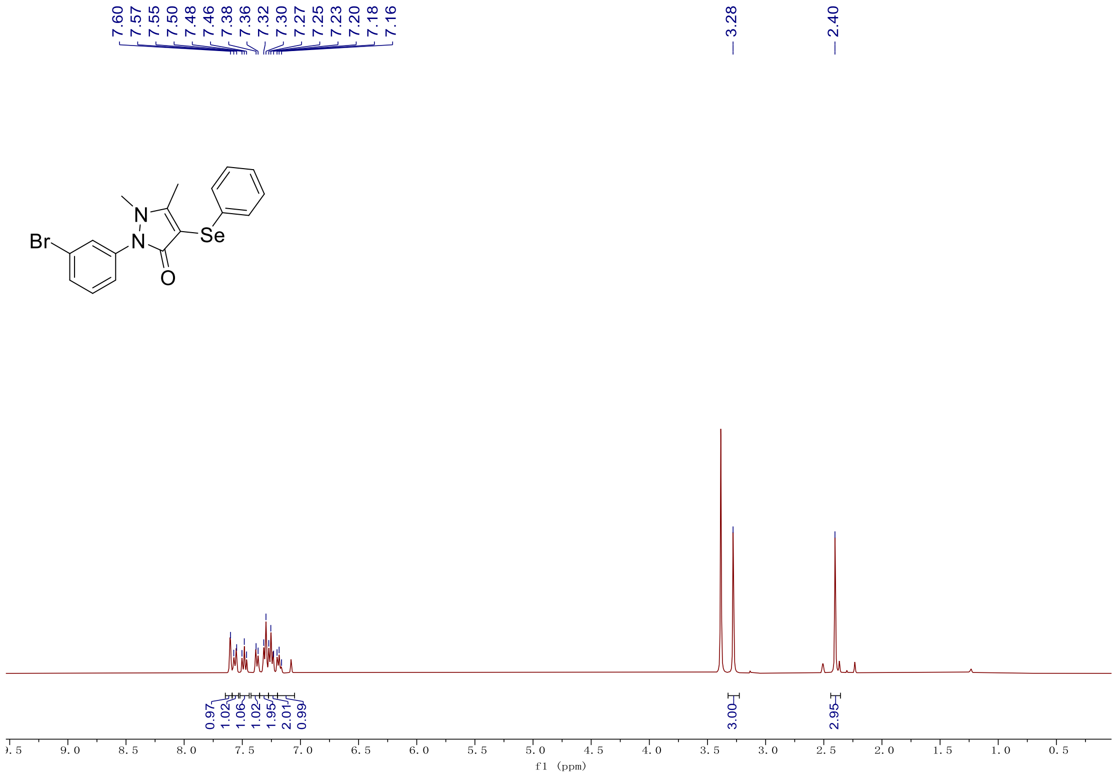

^{13}C NMR (101 MHz, DMSO-d_6) of compound **3k**

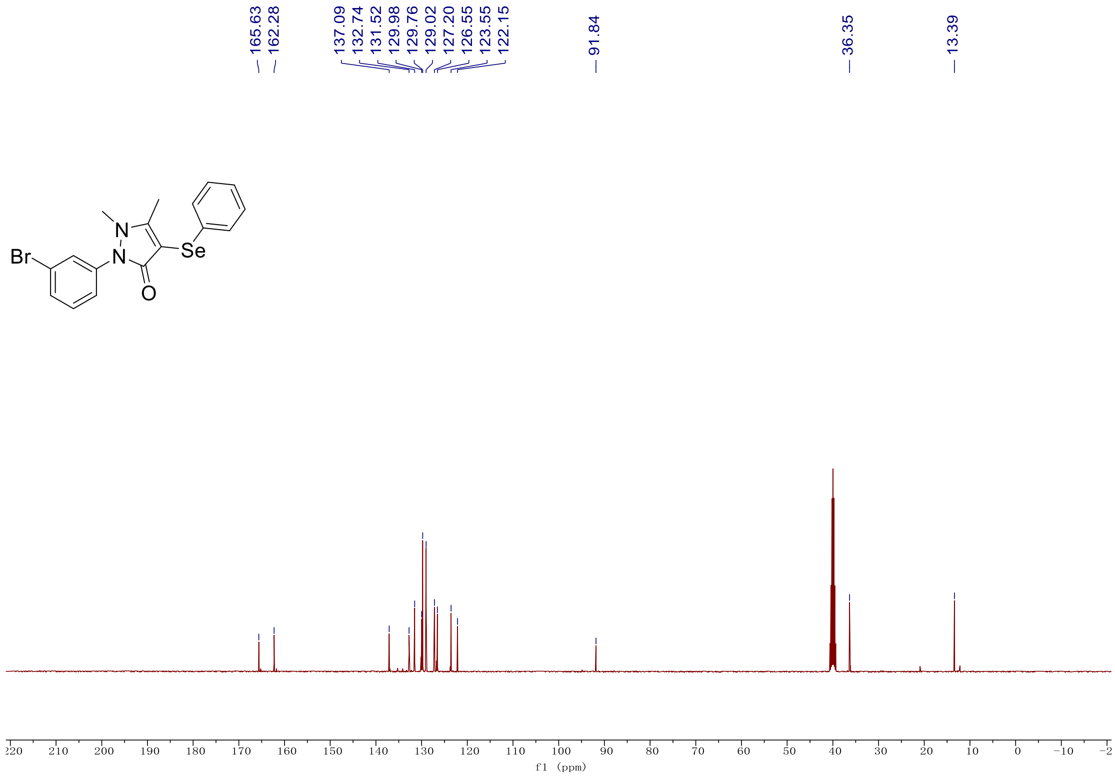

^{1}H NMR (400 MHz, DMSO-d_6) of compound **3l**

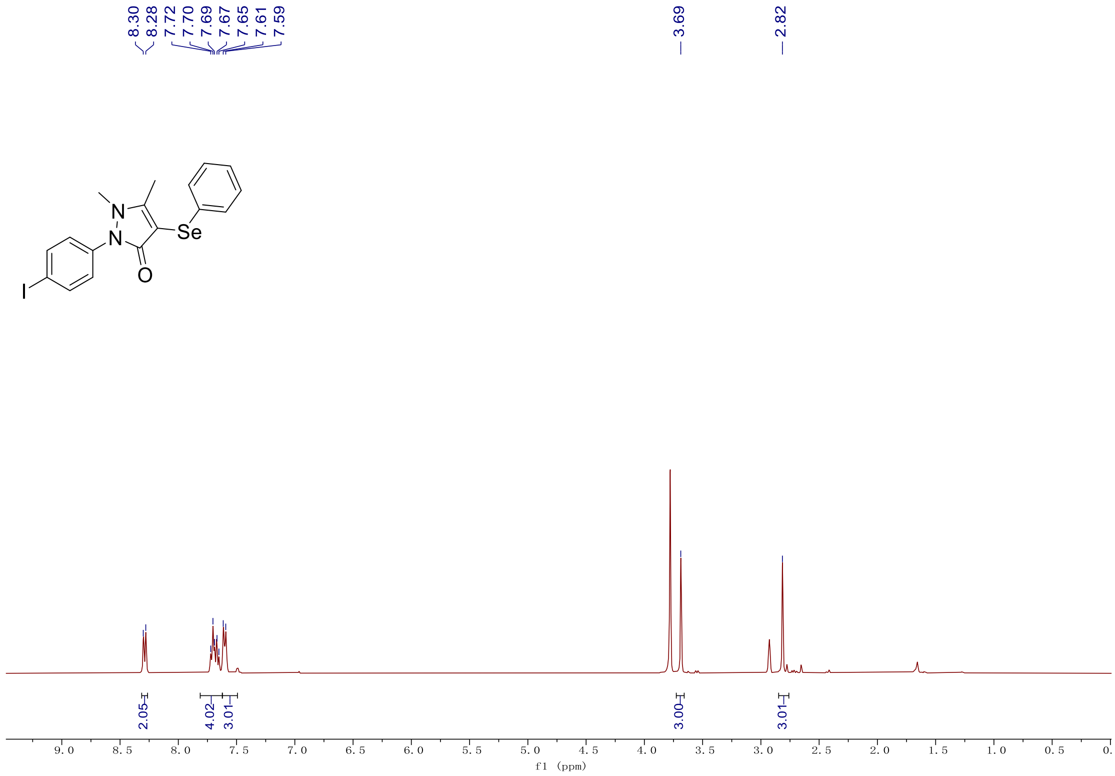

^{13}C NMR (101 MHz, DMSO-d_6) of compound **3l**

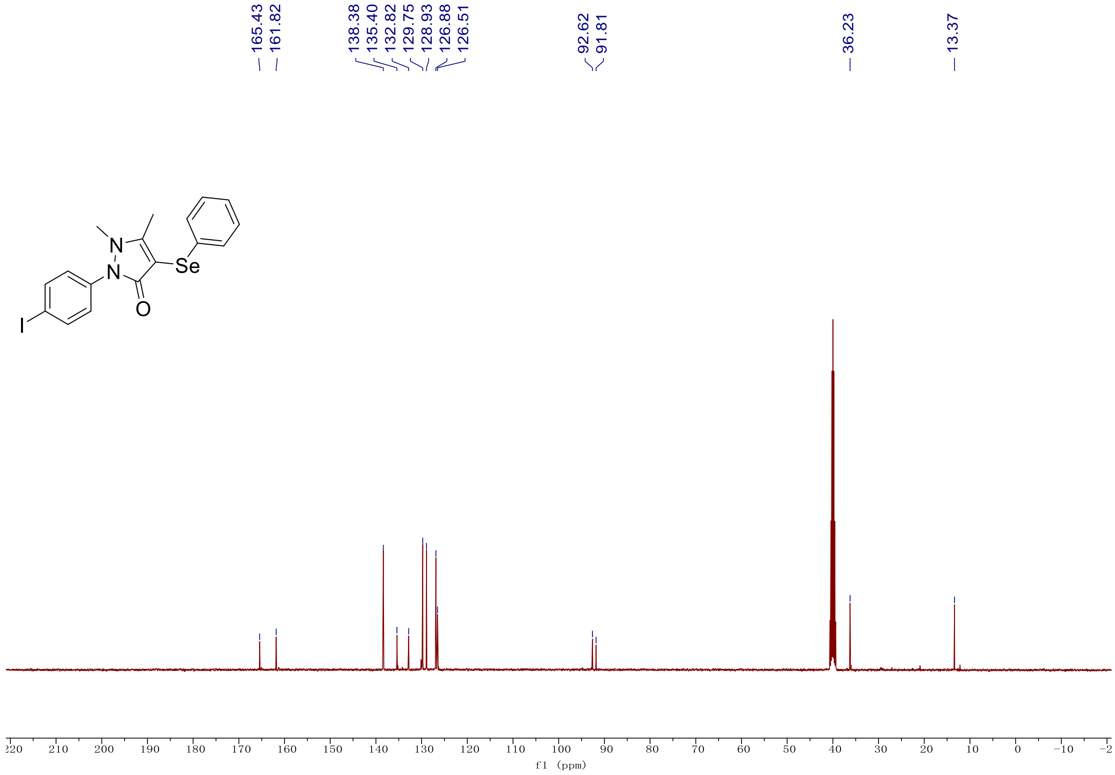

^{1}H NMR (400 MHz, DMSO-d_6) of compound **3m**

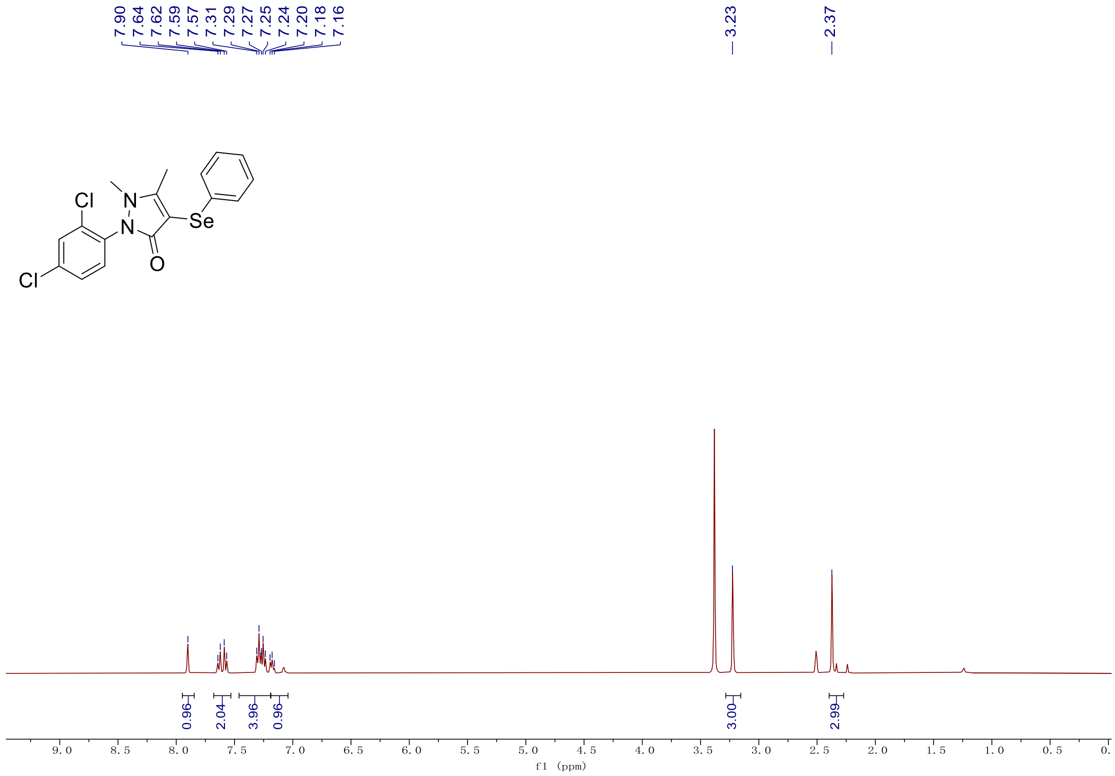

^{13}C NMR (101 MHz, DMSO-d_6) of compound **3m**

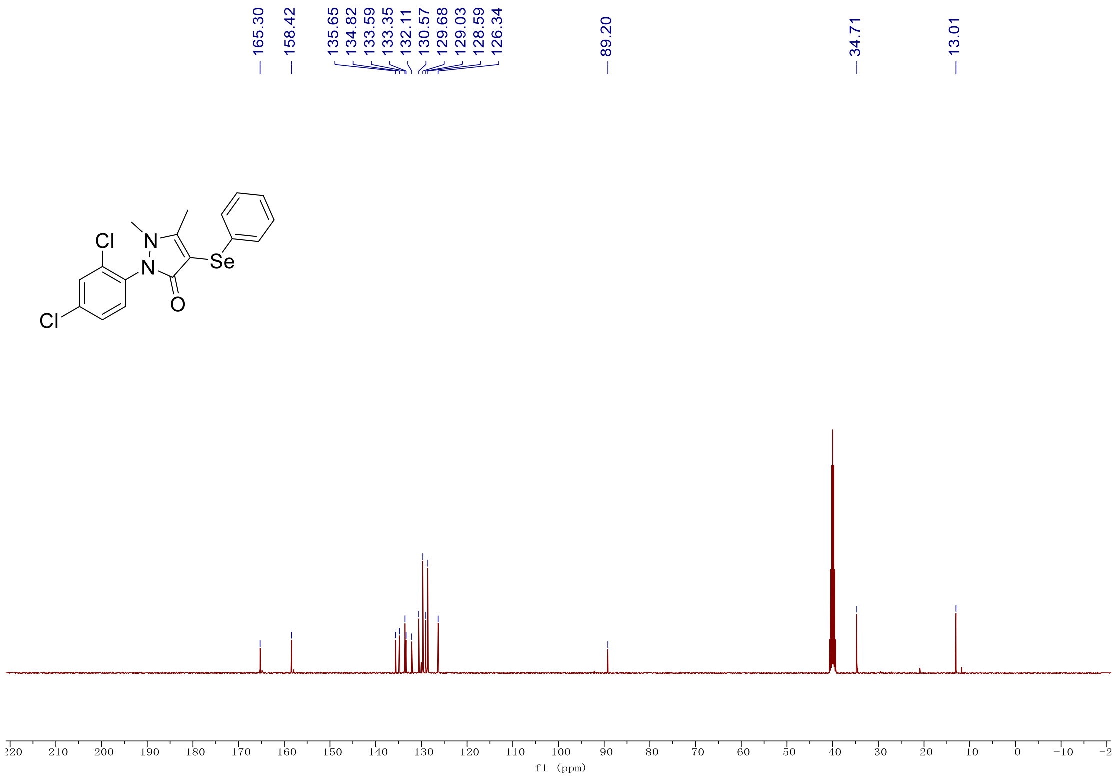

^{1}H NMR (400 MHz, DMSO-d_6) of compound **3n**

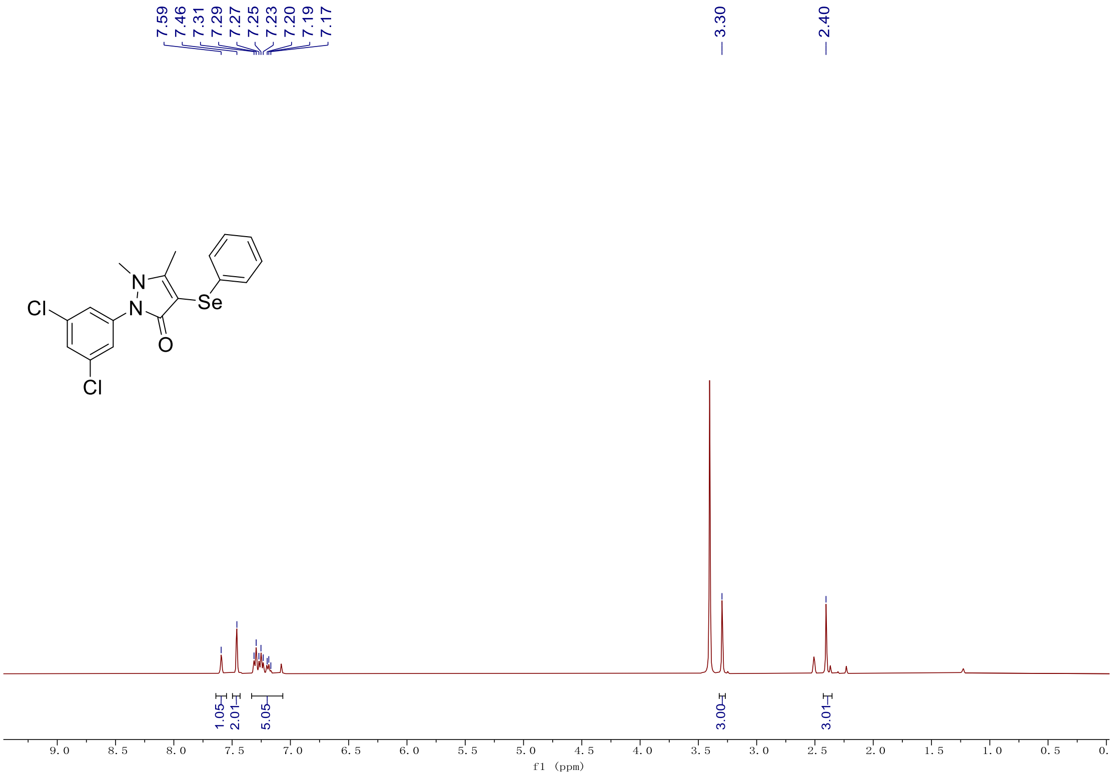

^{13}C NMR (101 MHz, DMSO-d_6) of compound **3n**

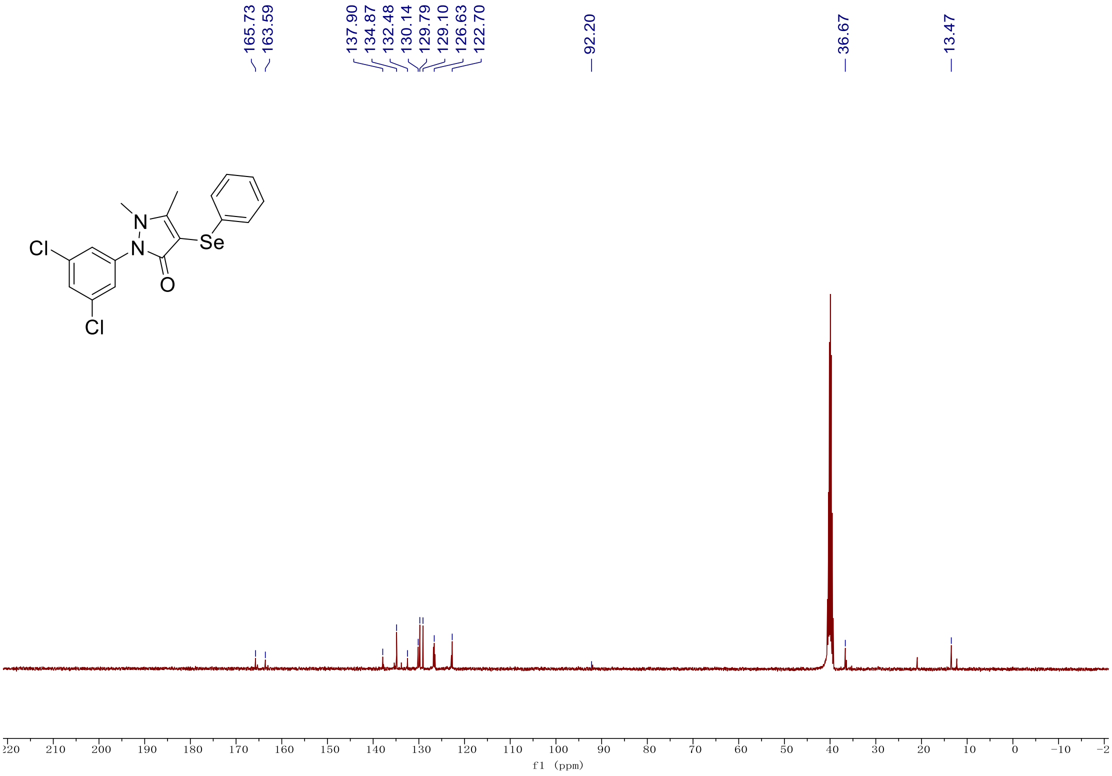

^{1}H NMR (400 MHz, DMSO-d_6) of compound **3o**

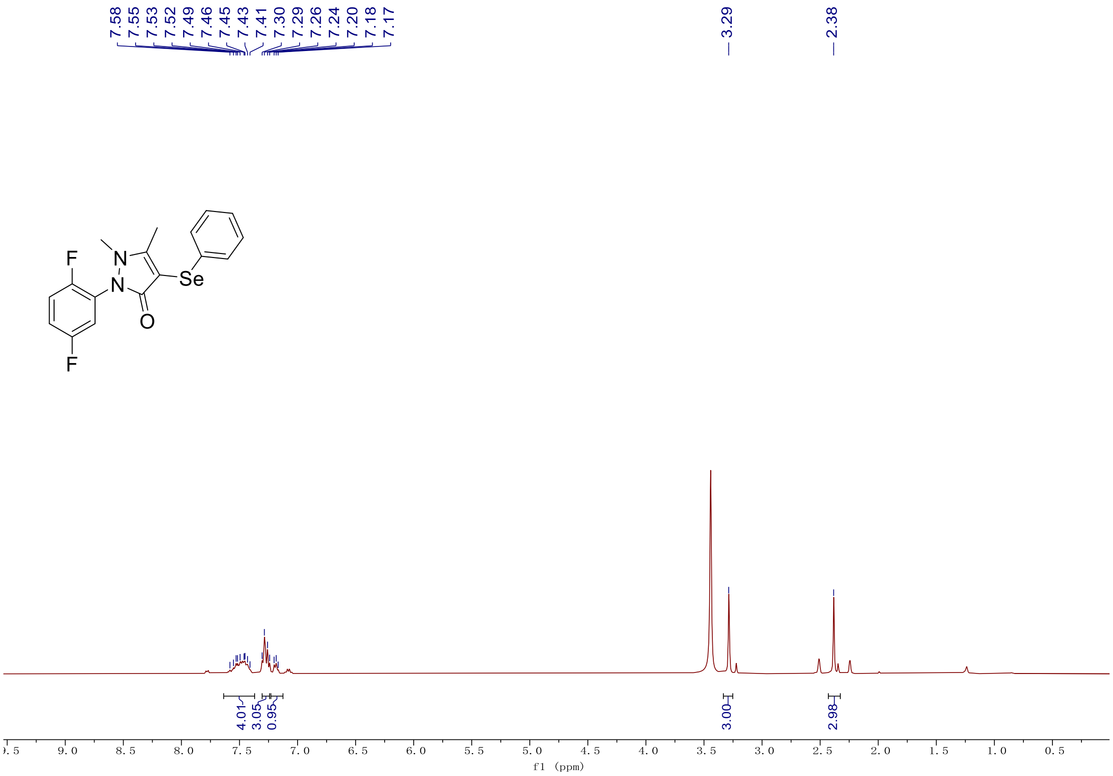

^{13}C NMR (101 MHz, DMSO-d_6) of compound **3o**

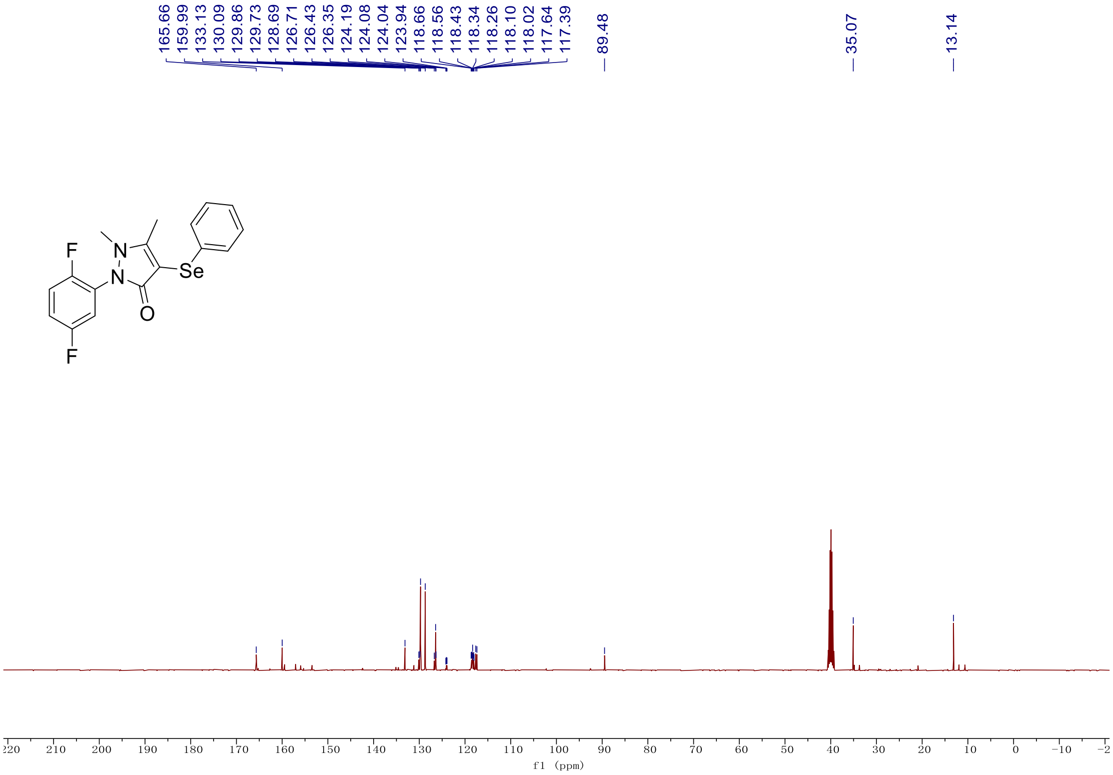

^{19}F NMR (376 MHz, DMSO-d_6) of compound **3o**

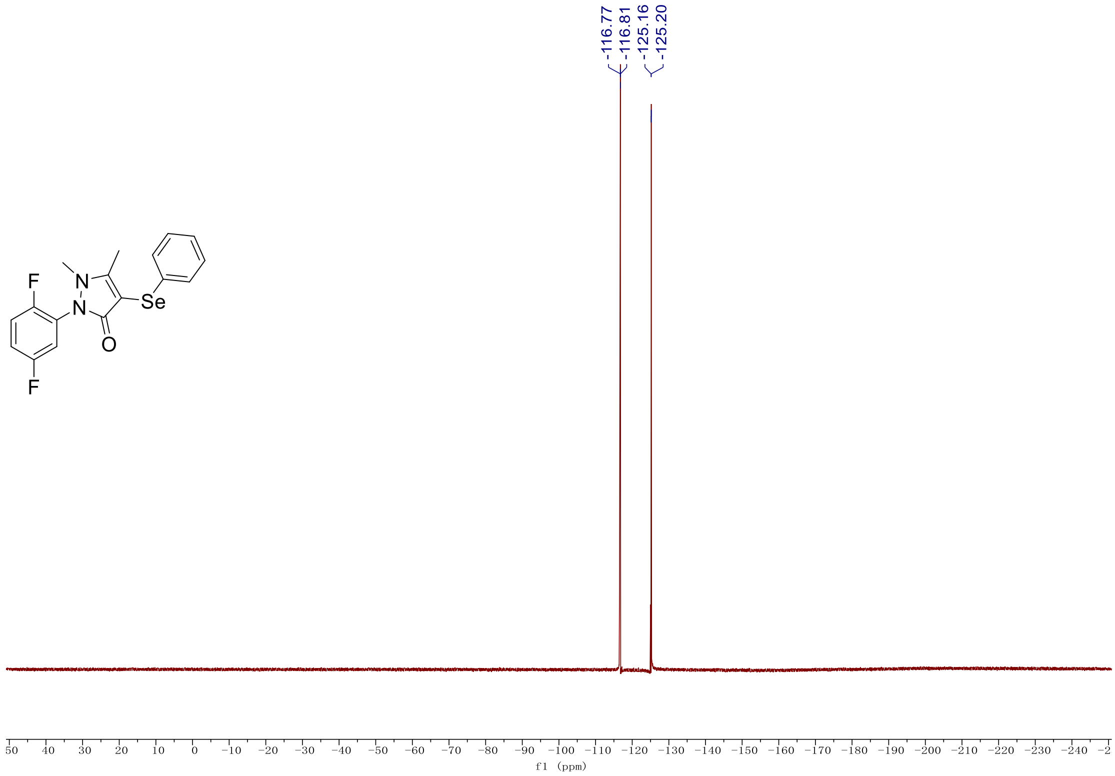

^{1}H NMR (400 MHz, DMSO-d_6) of compound **3p**

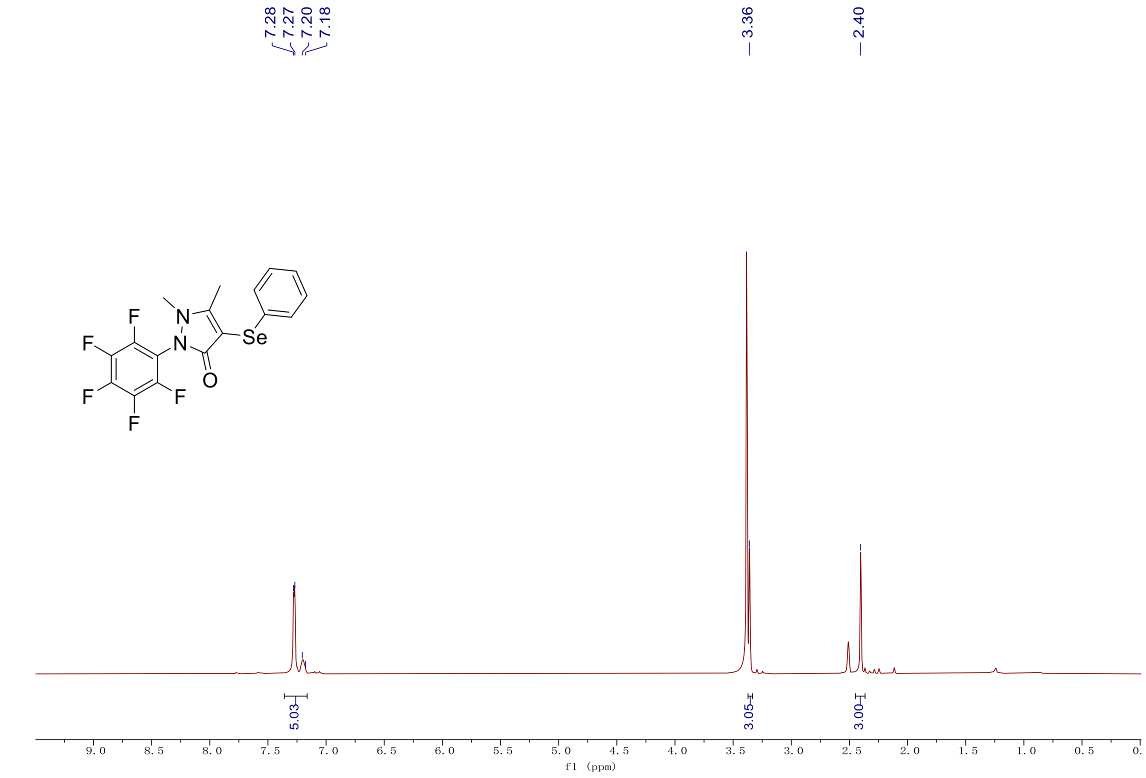

^{13}C NMR (101 MHz, DMSO-d_6) of compound **3p**

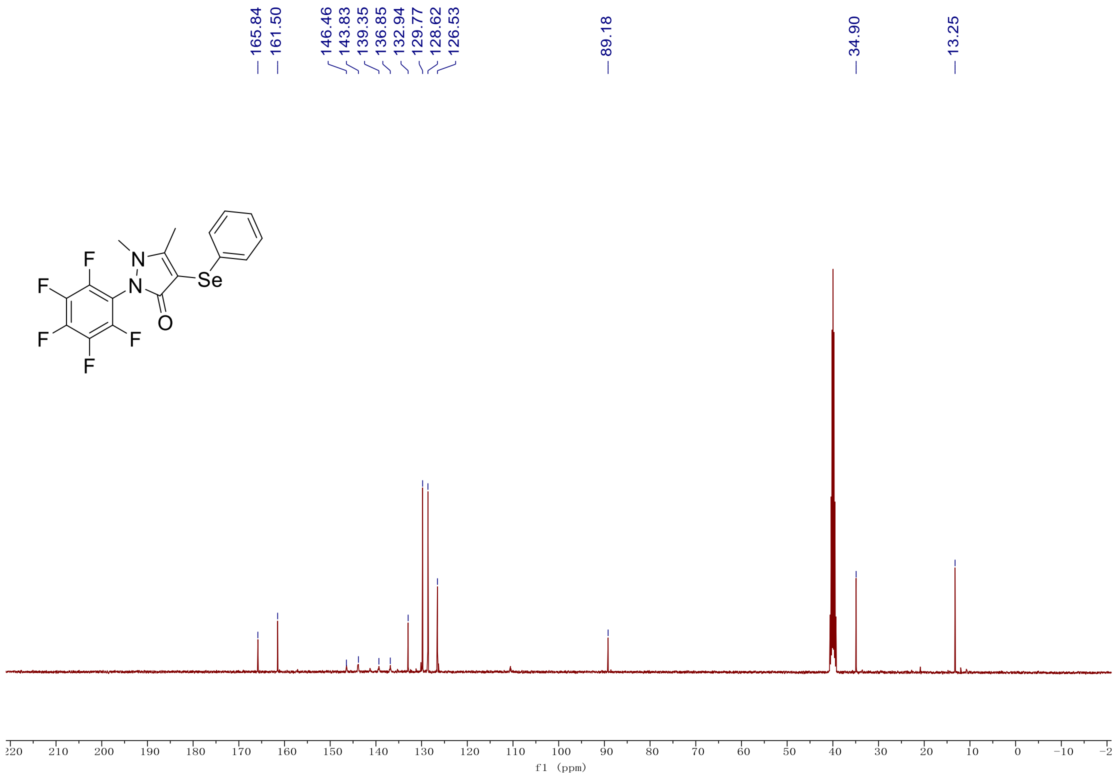

^{19}F NMR (376 MHz, DMSO-d_6) of compound **3p**

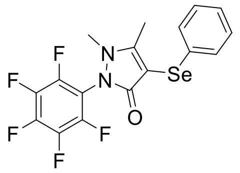

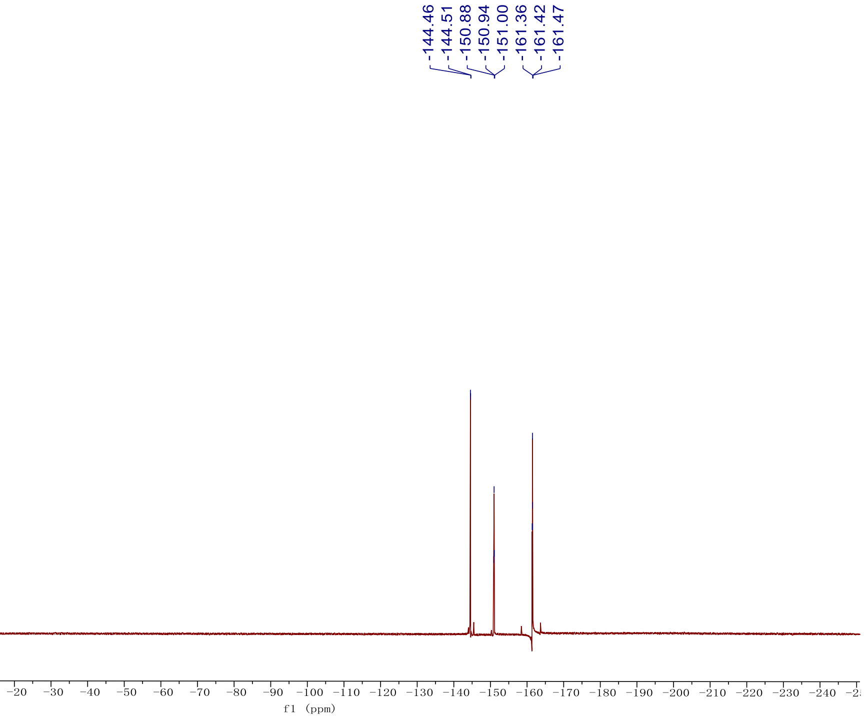

^{1}H NMR (400 MHz, DMSO-d_6) of compound **3q**

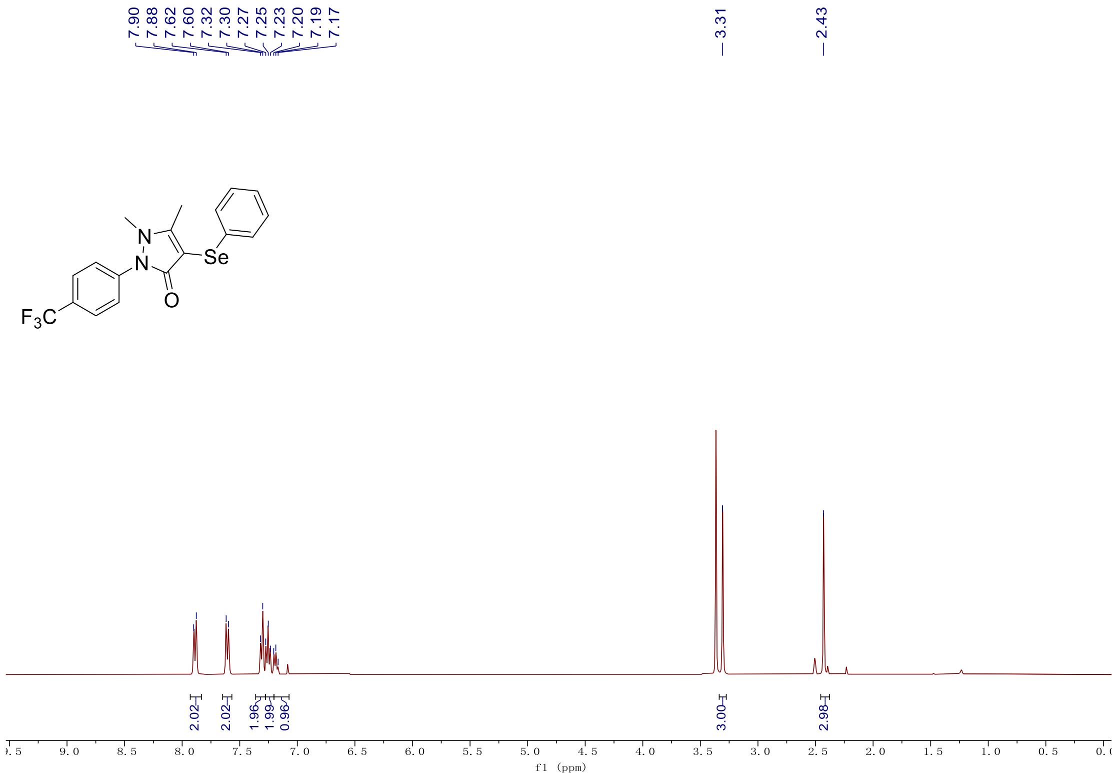

^{13}C NMR (101 MHz, DMSO-d_6) of compound **3q**

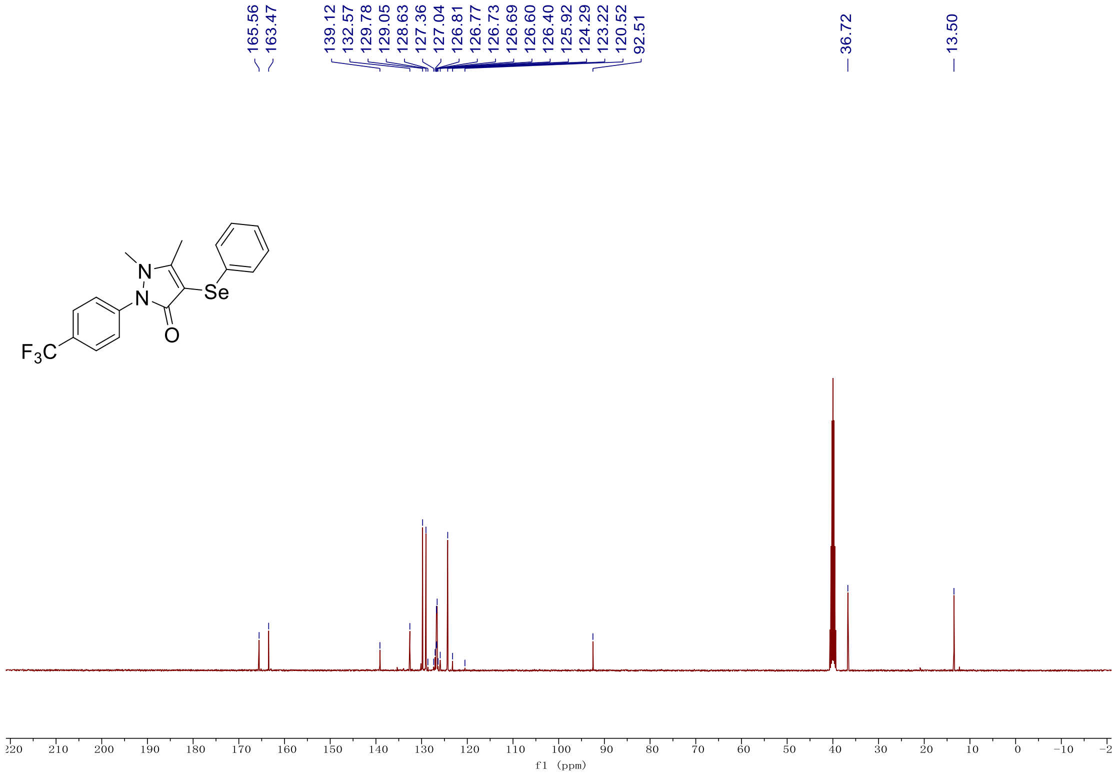

^{19}F NMR (376 MHz, DMSO-d_6) of compound **3q**

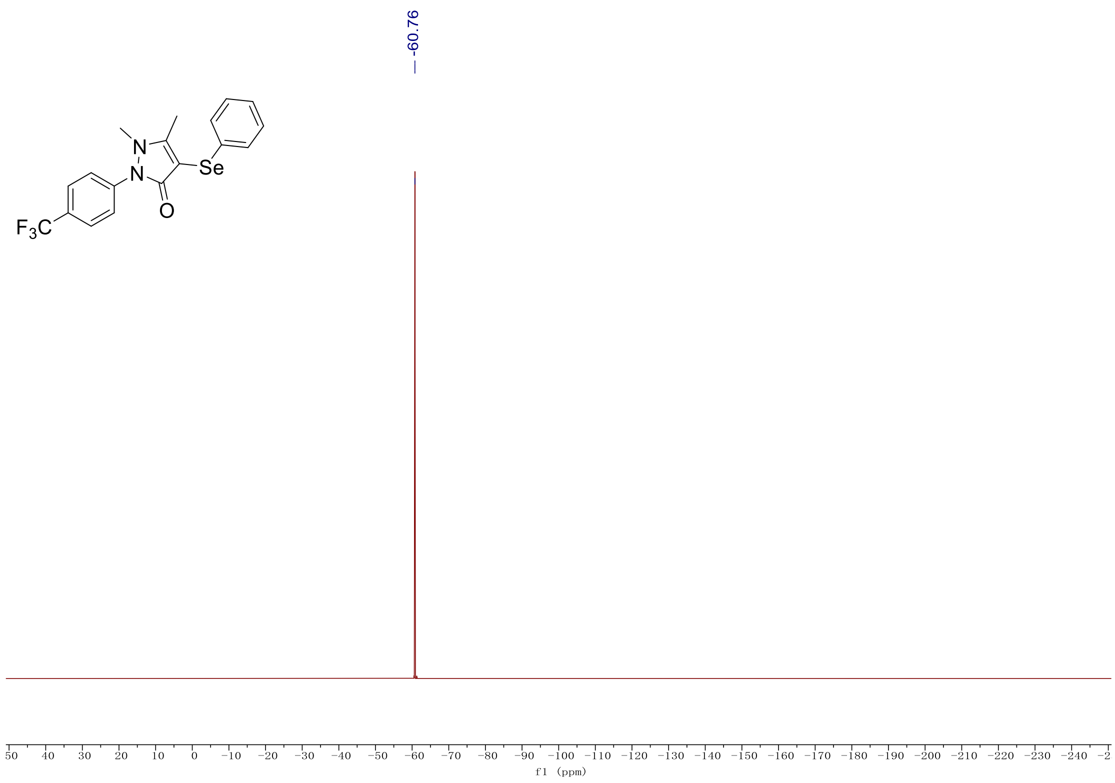

^{1}H NMR (400 MHz, DMSO-d_6) of compound **3r**

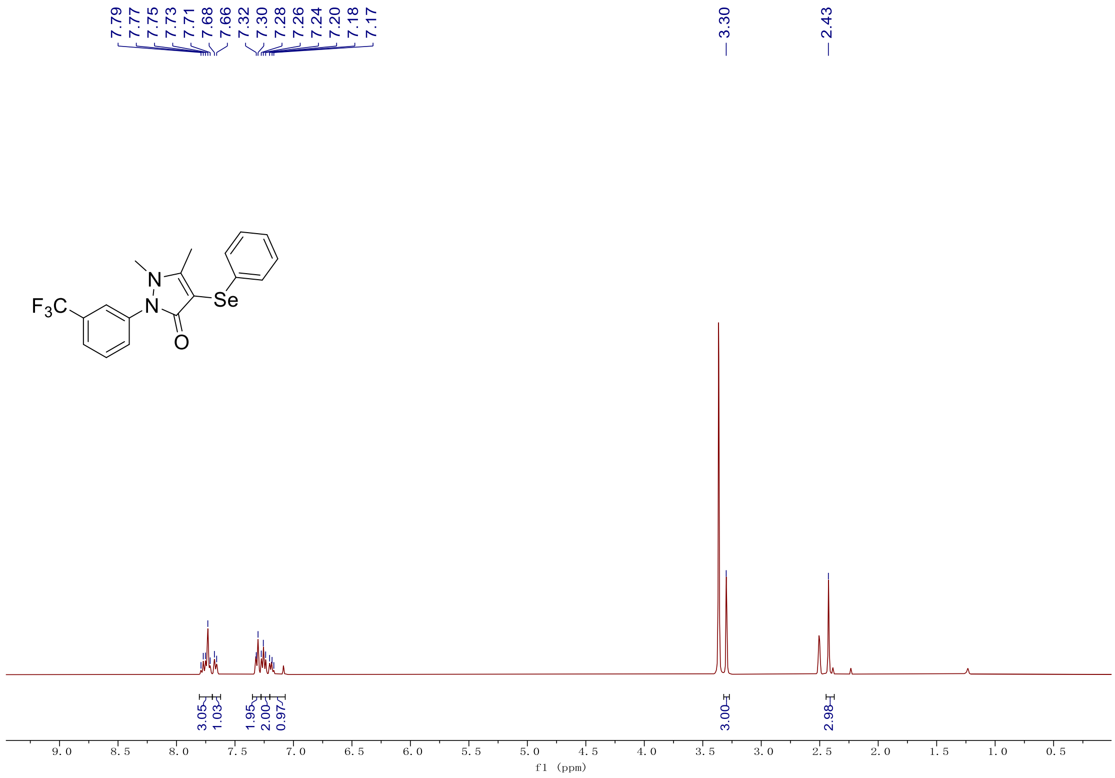

^{13}C NMR (101 MHz, DMSO-d_6) of compound **3r**

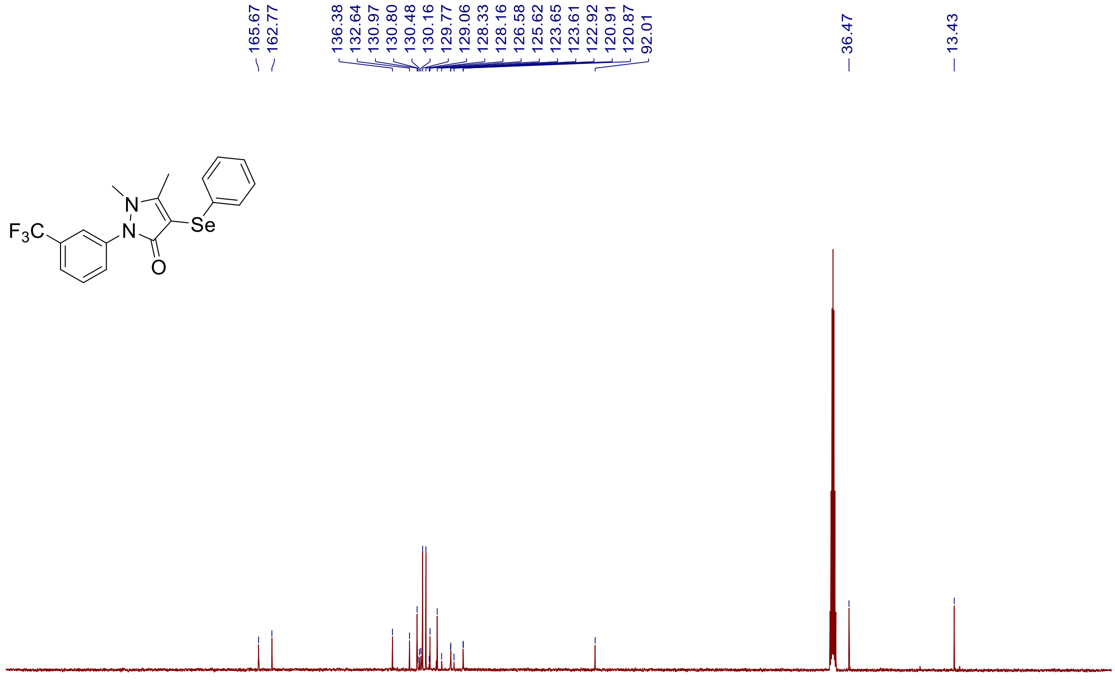

^{19}F NMR (376 MHz, DMSO-d_6) of compound **3r**

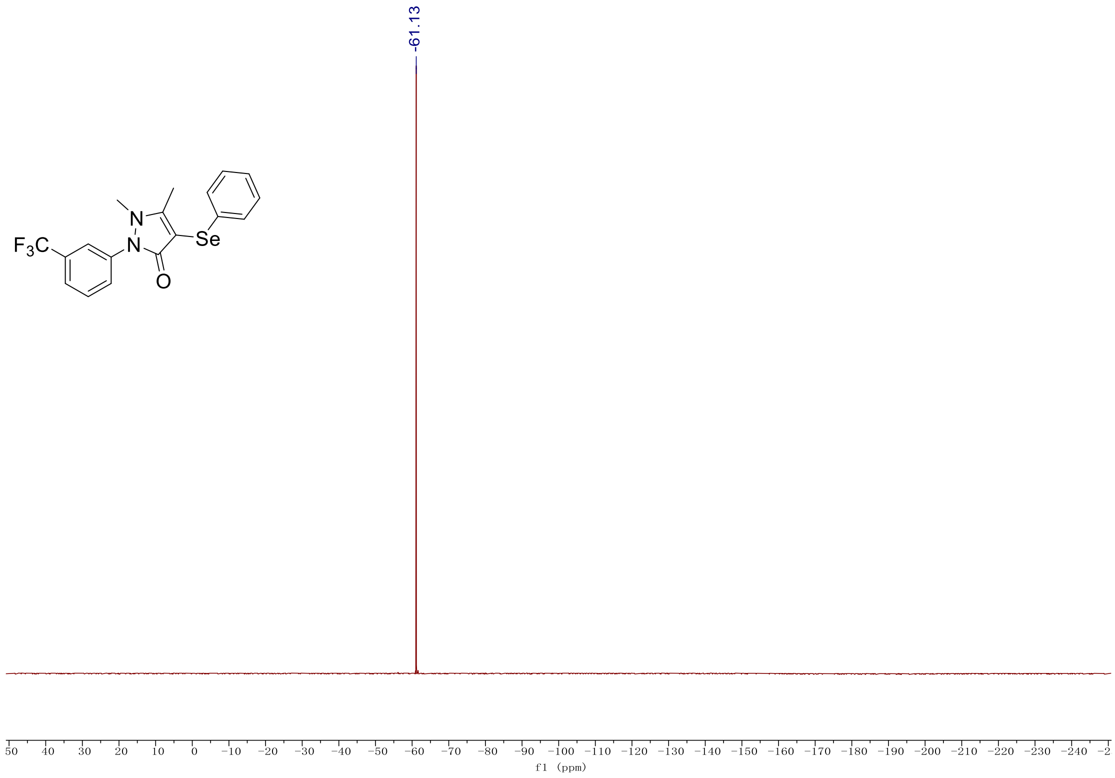

^{1}H NMR (400 MHz, DMSO-d_6) of compound **3s**

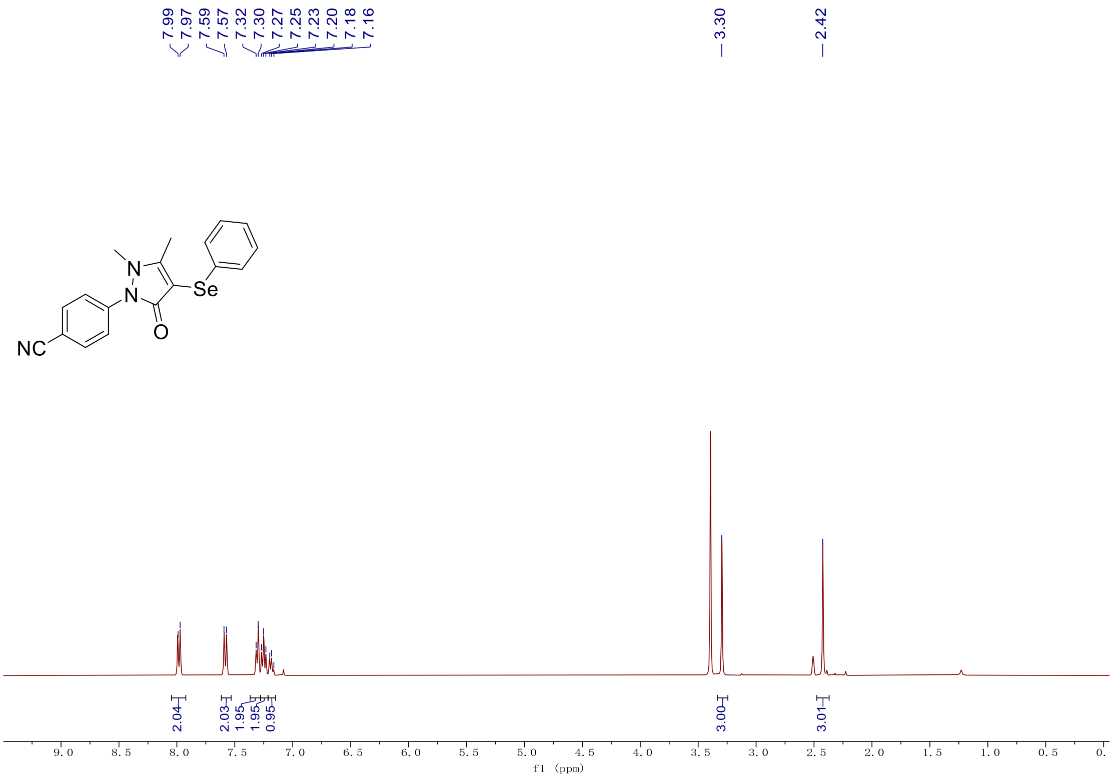

^{13}C NMR (101 MHz, DMSO-d_6) of compound **3s**

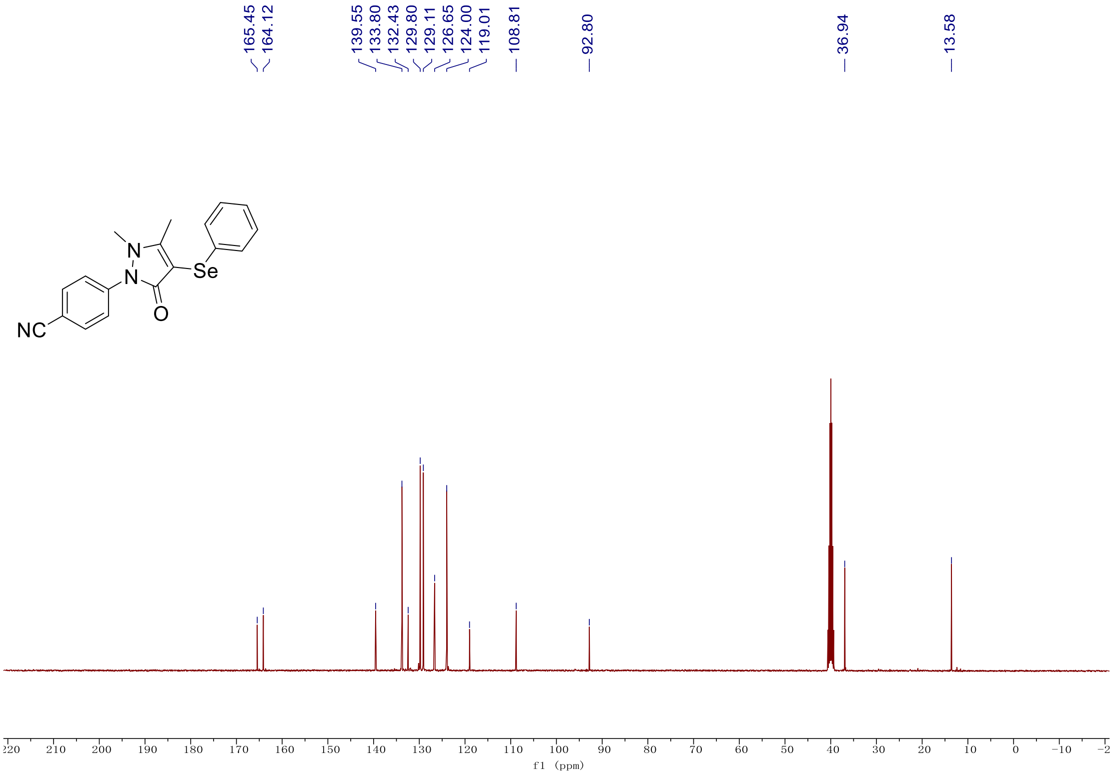

^{1}H NMR (400 MHz, DMSO-d_6) of compound **3t**

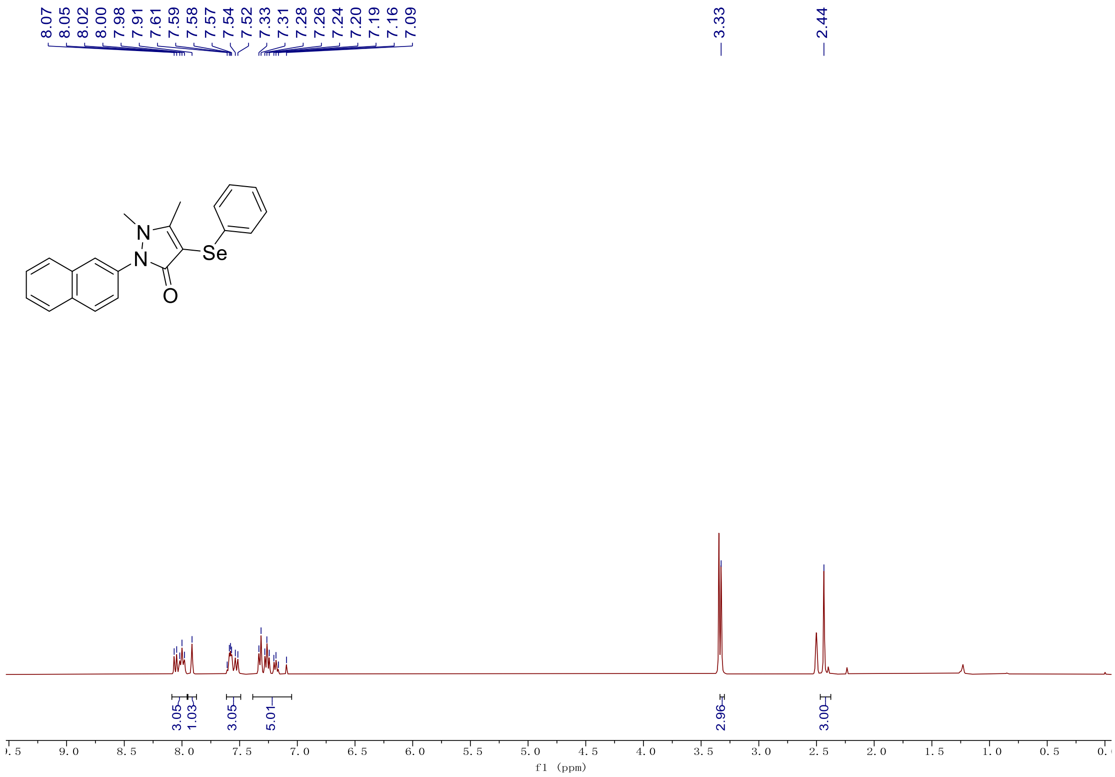

^{13}C NMR (101 MHz, DMSO-d_6) of compound **3t**

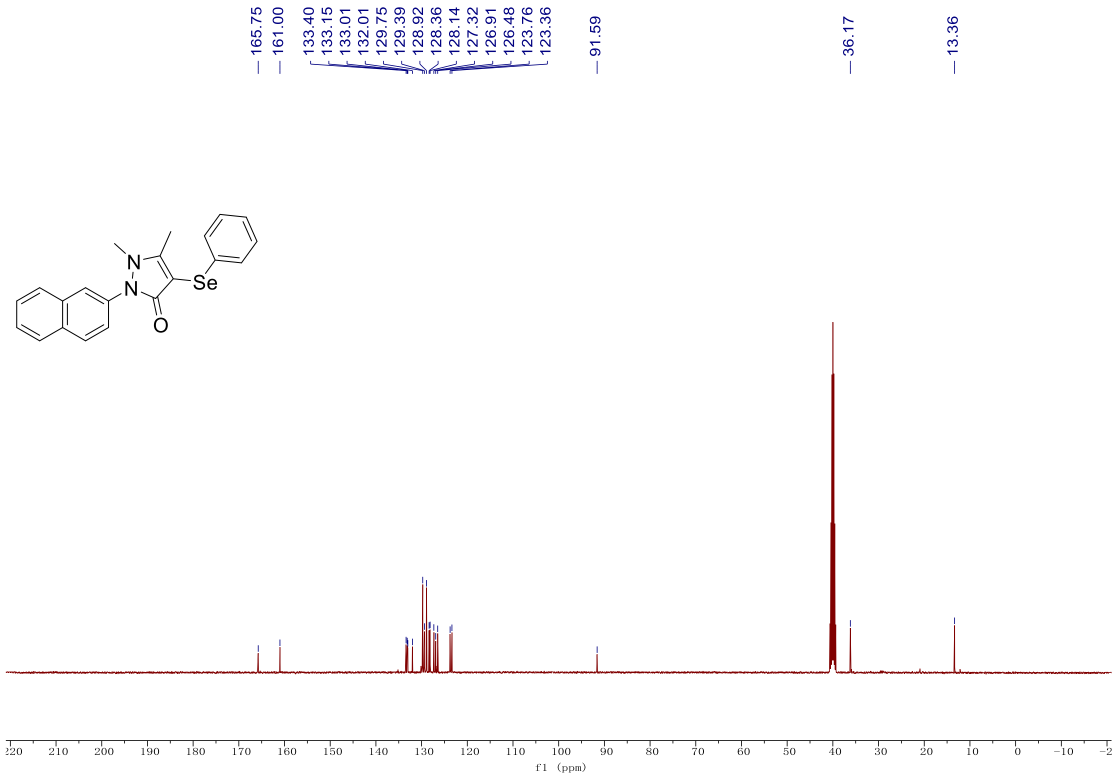

^{1}H NMR (600 MHz, DMSO-d_6) of compound **3u**

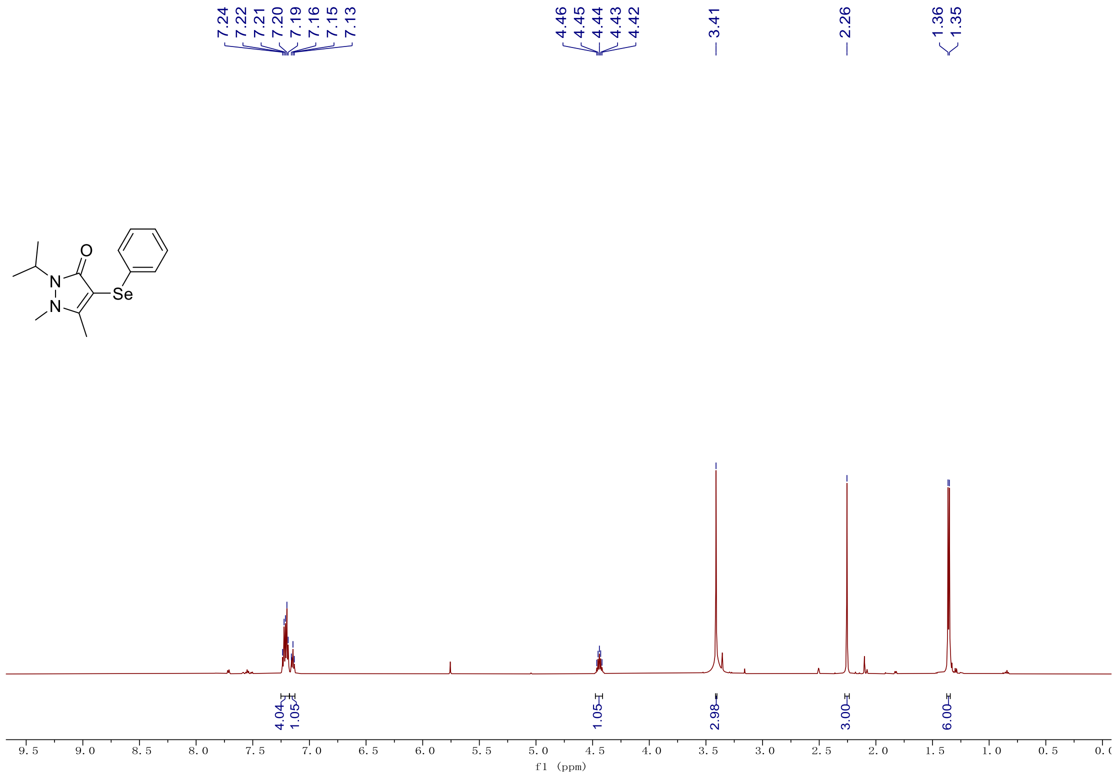

^{13}C NMR (151 MHz, DMSO-d_6) of compound **3u**

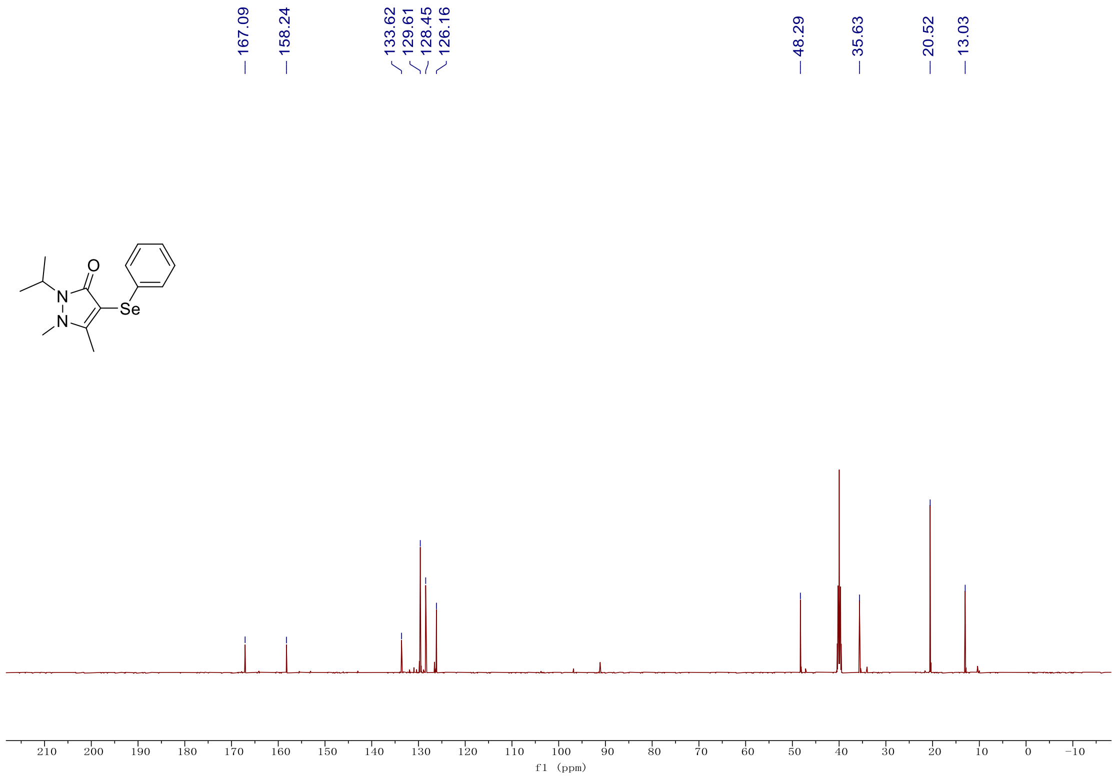

^{1}H NMR (600 MHz, DMSO-d_6) of compound **3v**

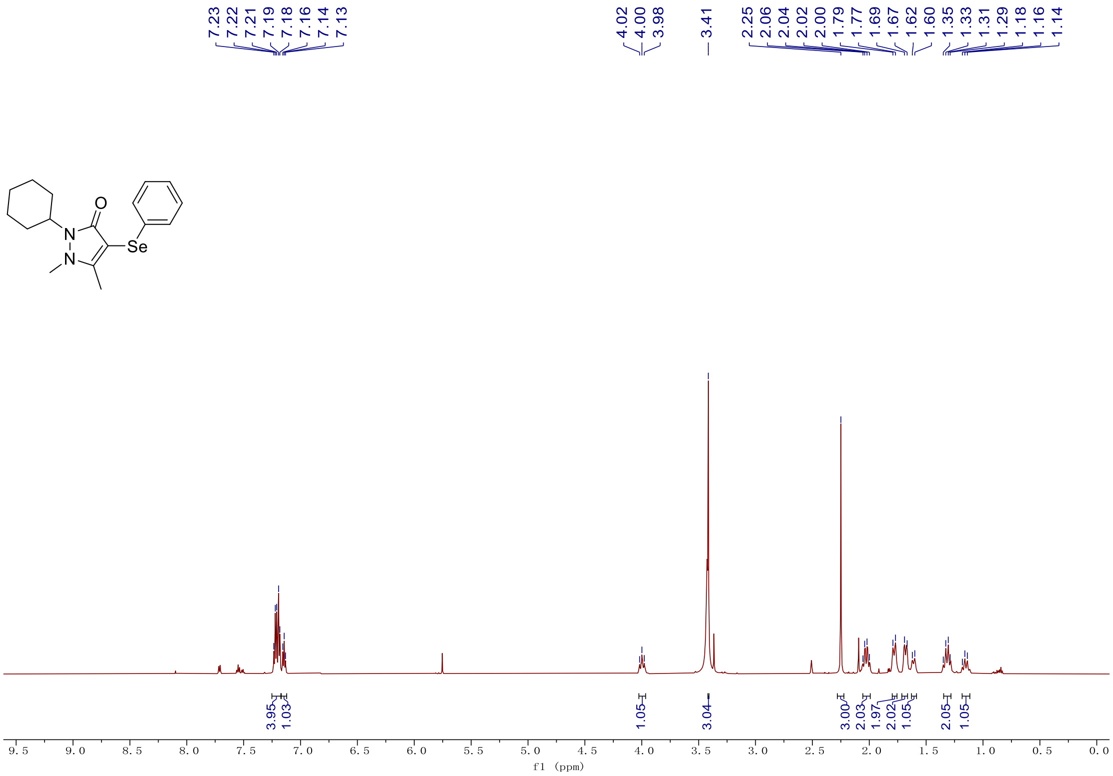

^{13}C NMR (151 MHz, DMSO-d_6) of compound **3v**

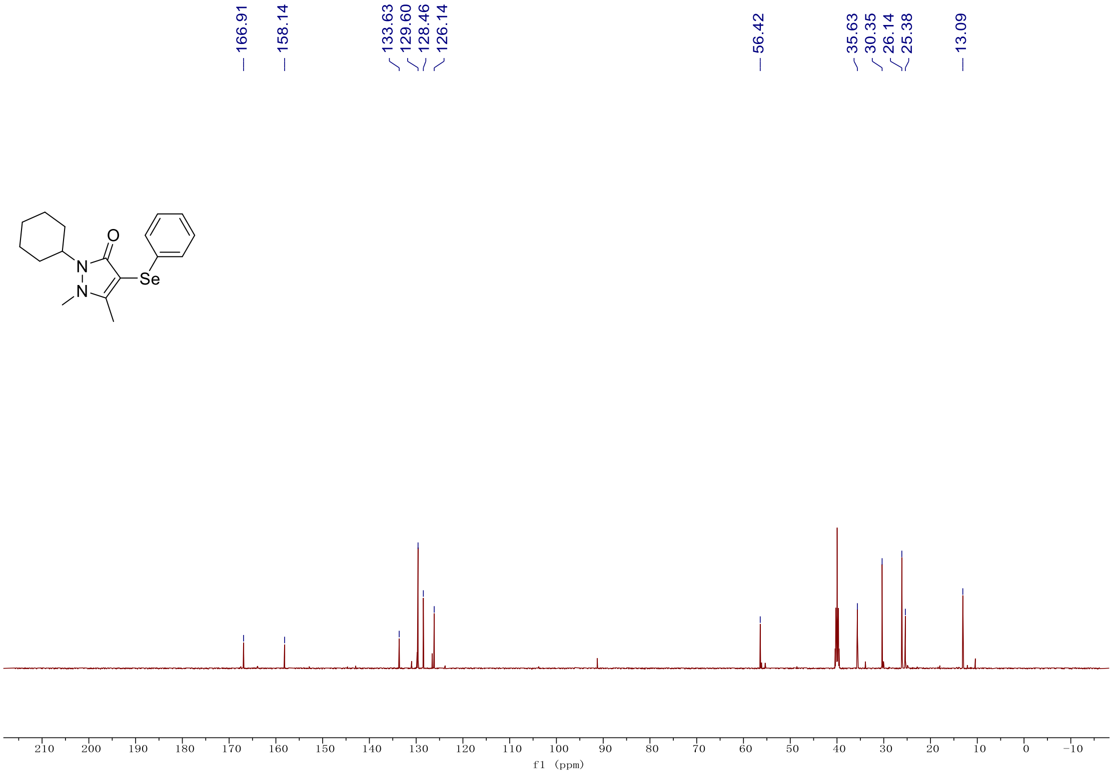

^{1}H NMR (600 MHz, DMSO-d_6) of compound **3w**

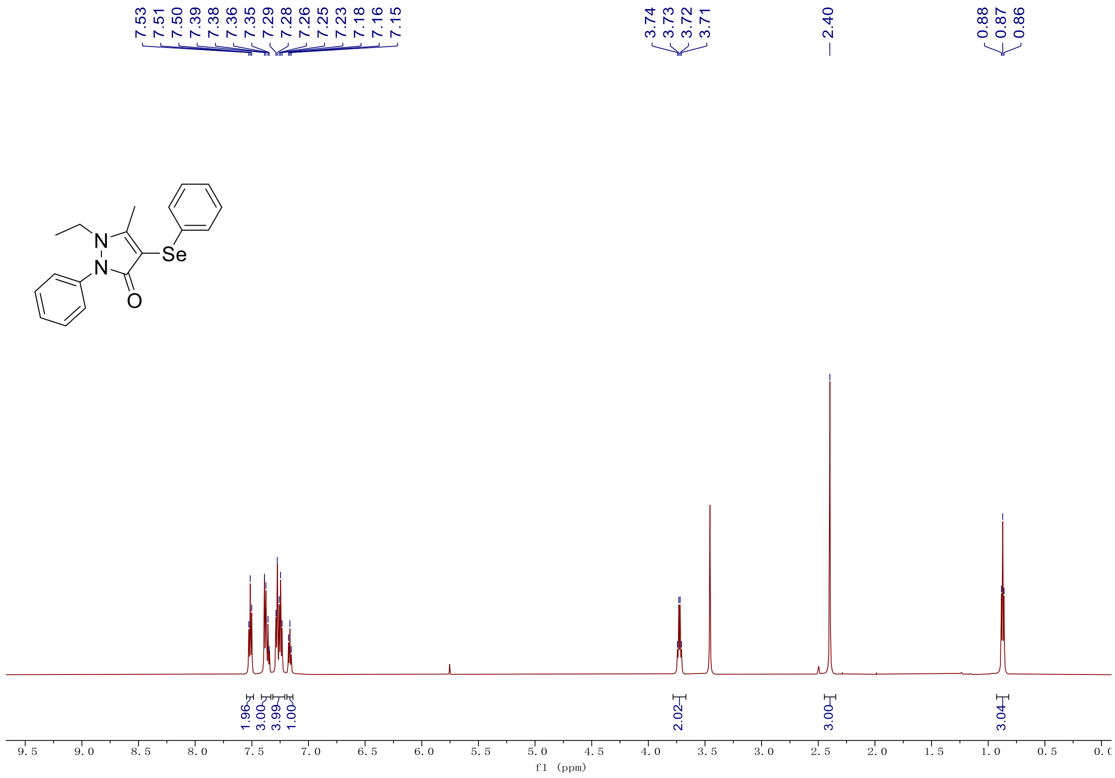

^{13}C NMR (151 MHz, DMSO-d_6) of compound **3w**

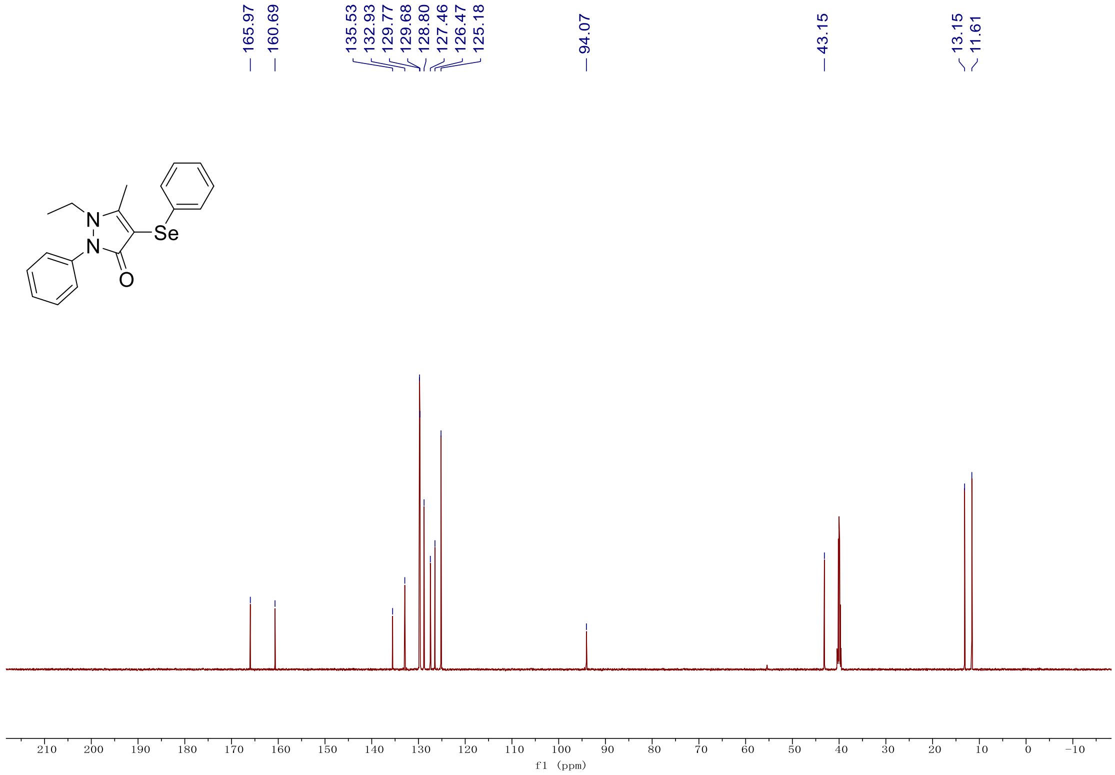

^{1}H NMR (400 MHz, DMSO-d_6) of compound **3x**

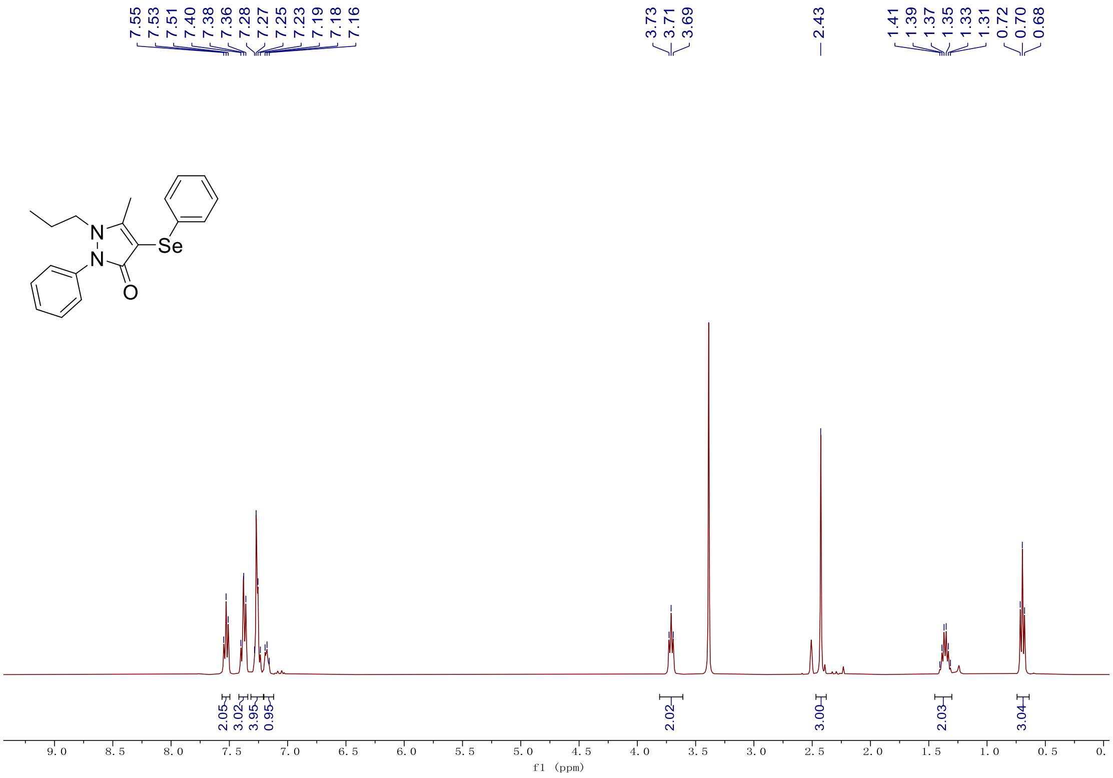

^{13}C NMR (101 MHz, DMSO-d_6) of compound **3x**

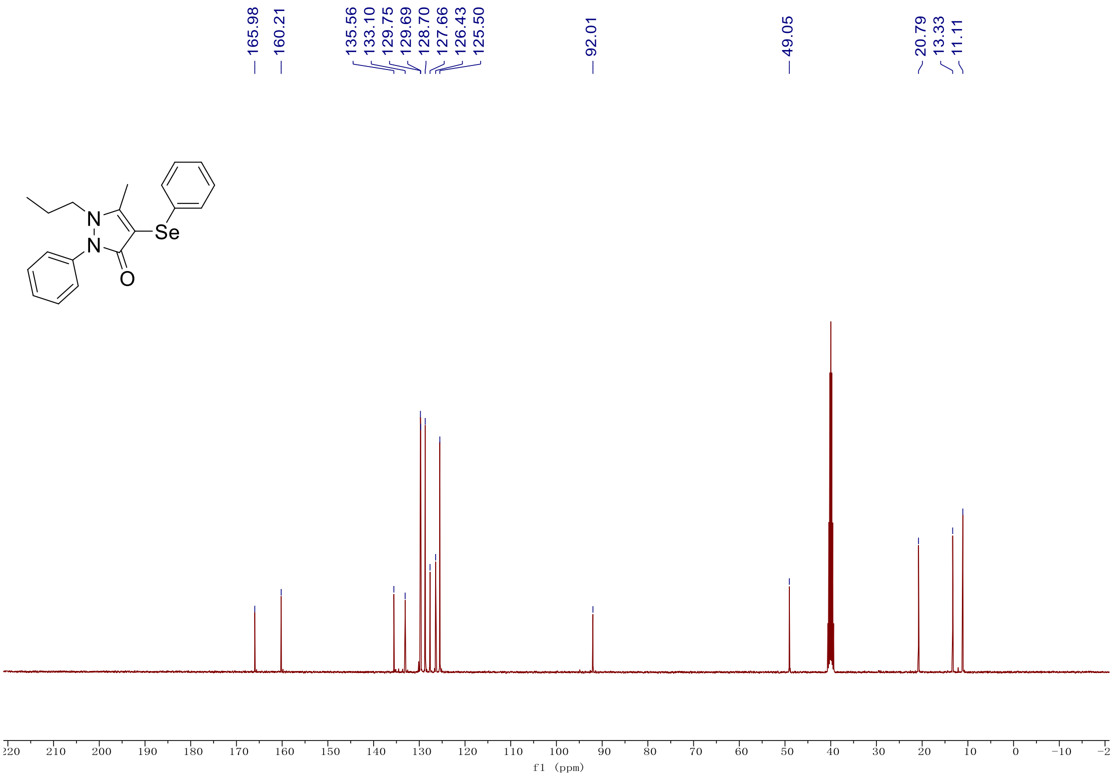

^{1}H NMR (400 MHz, DMSO-d_6) of compound **3y**

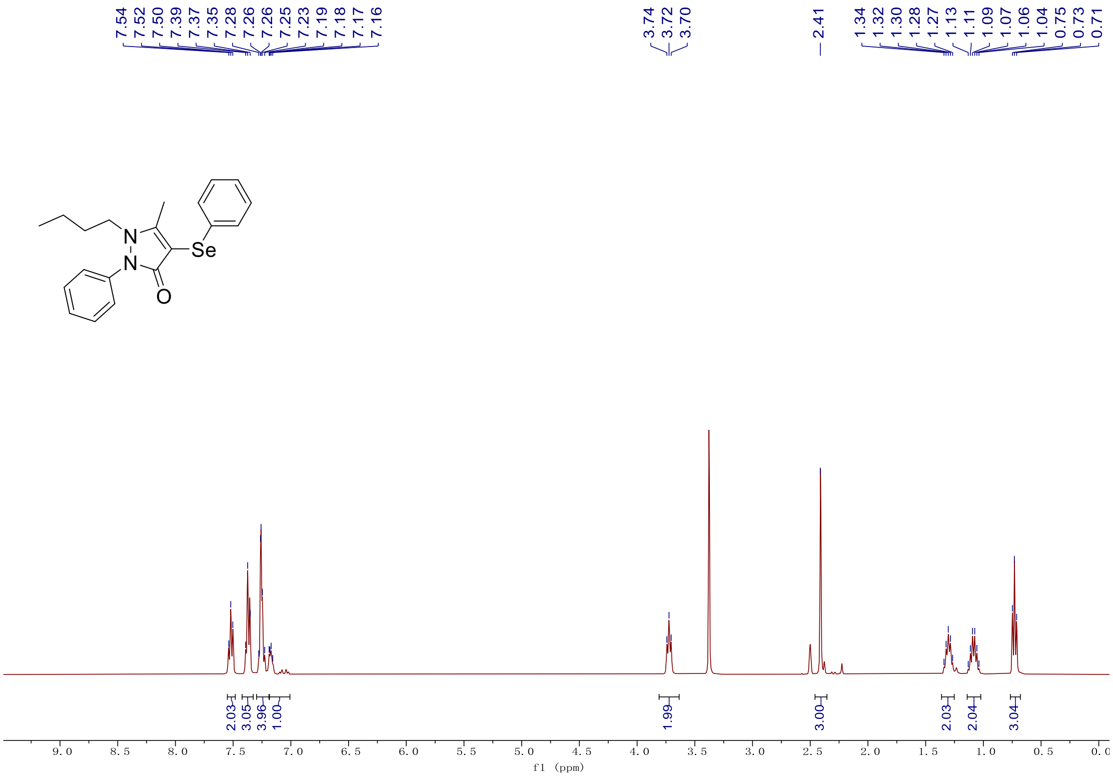

^{13}C NMR (101 MHz, DMSO-d_6) of compound **3y**

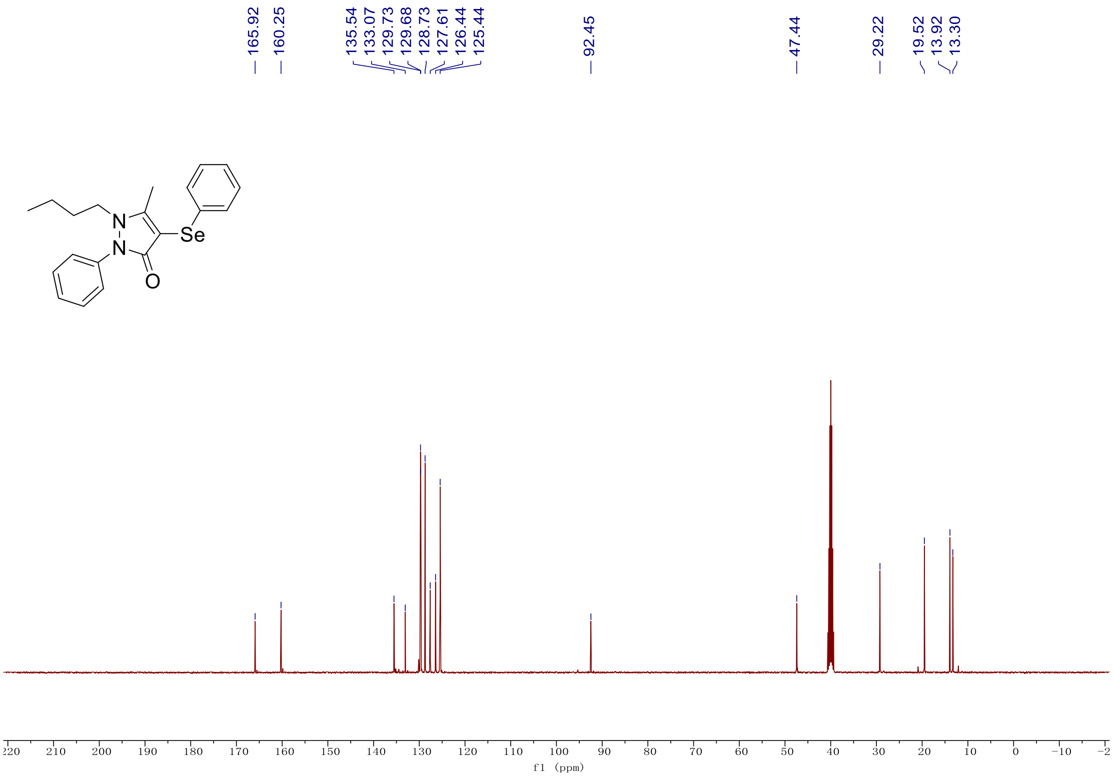

^{1}H NMR (600 MHz, DMSO-d_6) of compound **3z**

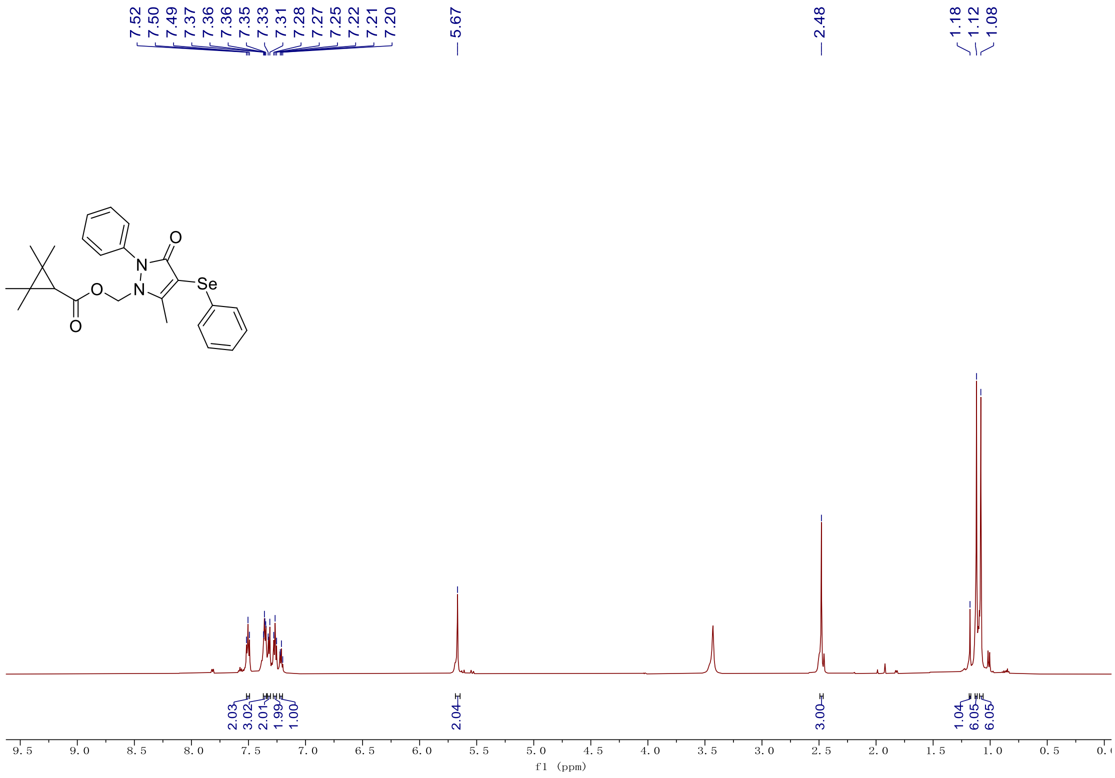

^{13}C NMR (151 MHz, DMSO-d_6) of compound **3z**

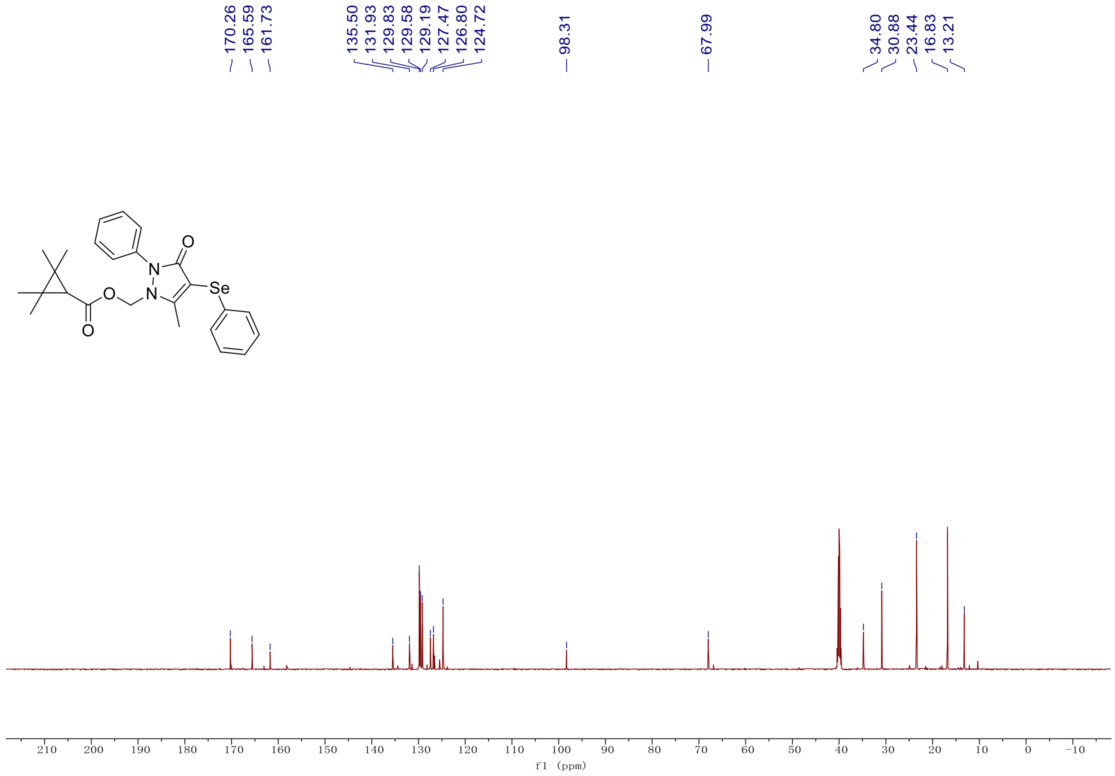

^{1}H NMR (600 MHz, DMSO-d_6) of compound **3za**

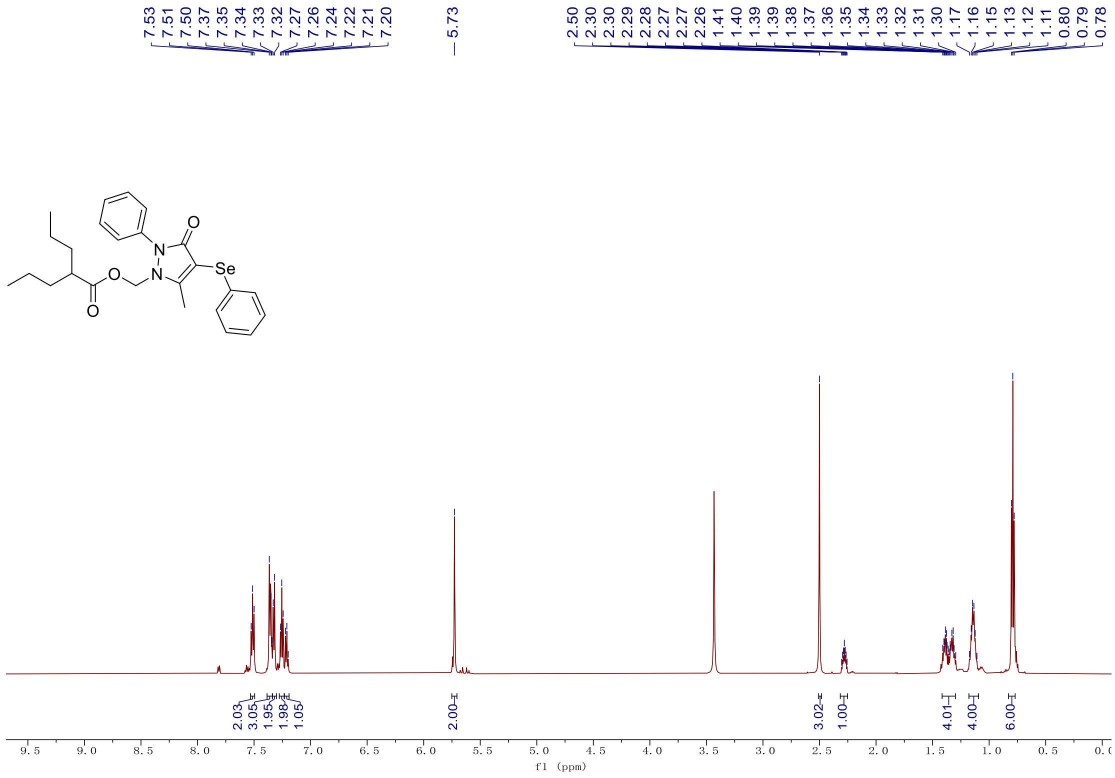

^{13}C NMR (151 MHz, DMSO-d_6) of compound **3za**

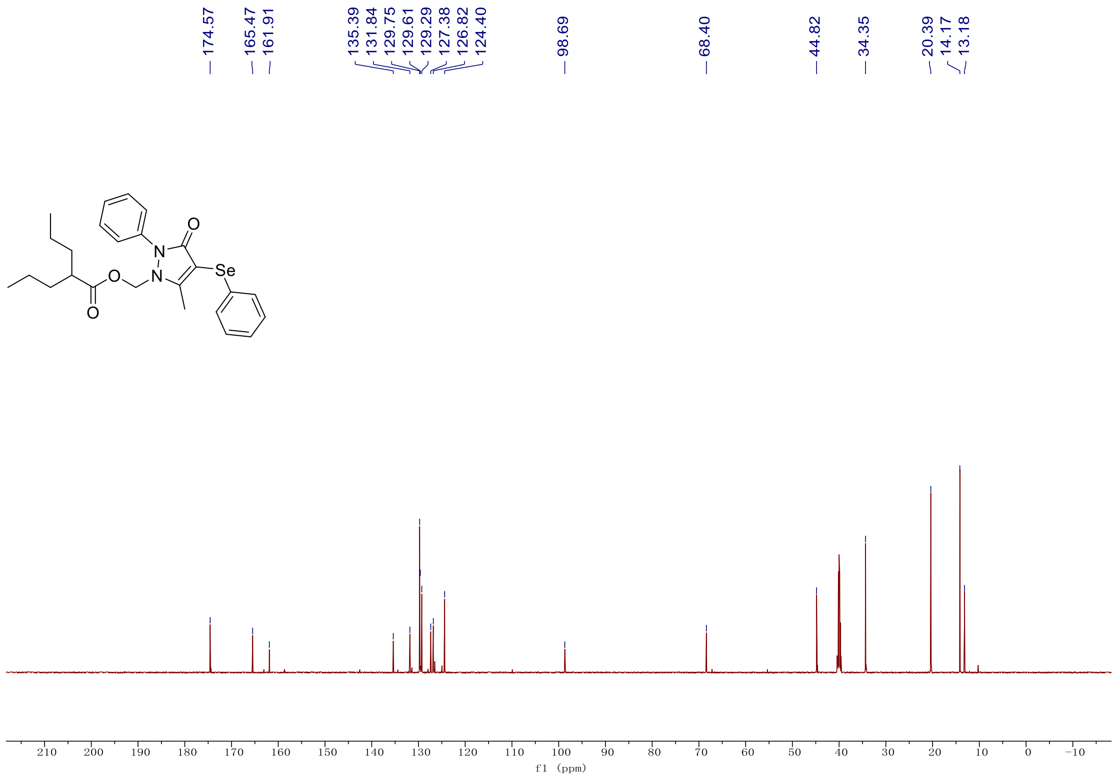

^{1}H NMR (400 MHz, DMSO-d_6) of compound **3zb**

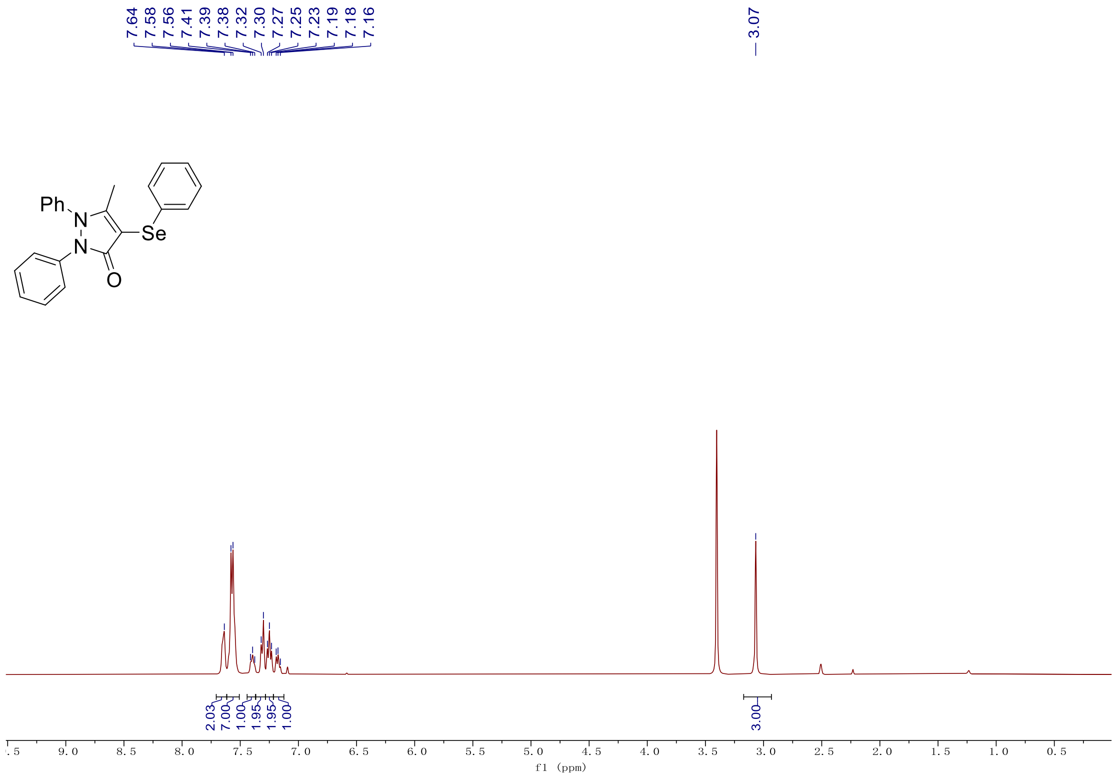

^{13}C NMR (101 MHz, DMSO-d_6) of compound **3zb**

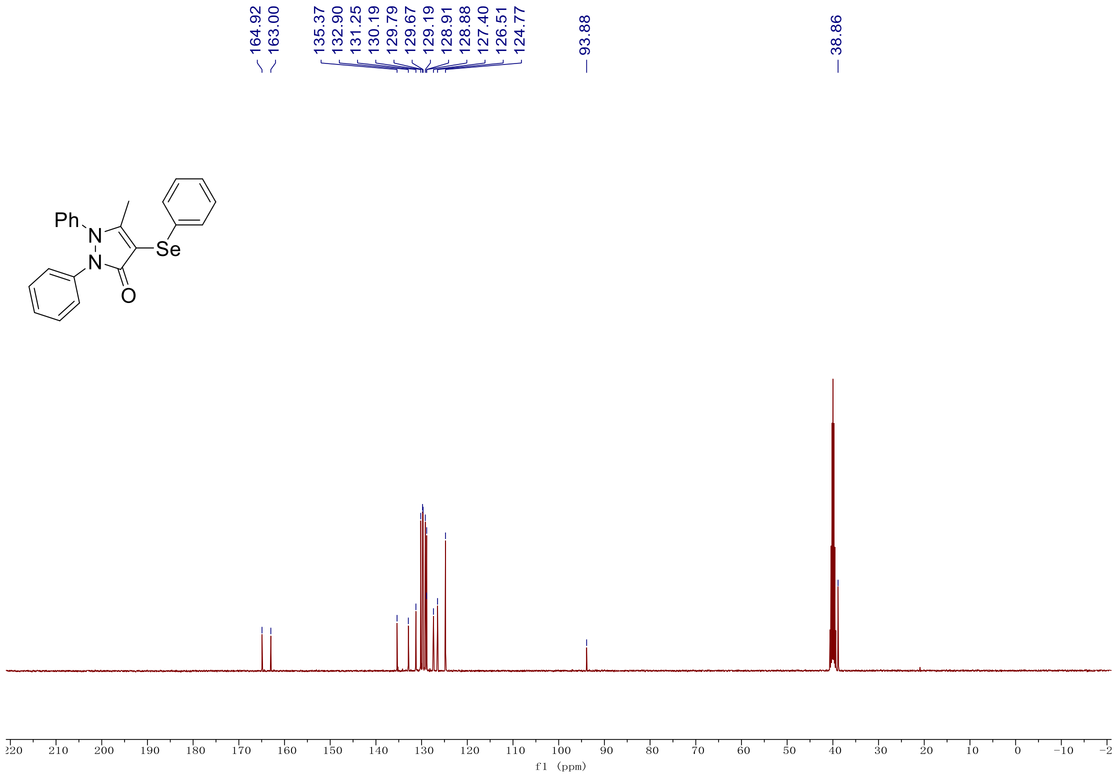

^{1}H NMR (400 MHz, DMSO-d_6) of compound **3zc**

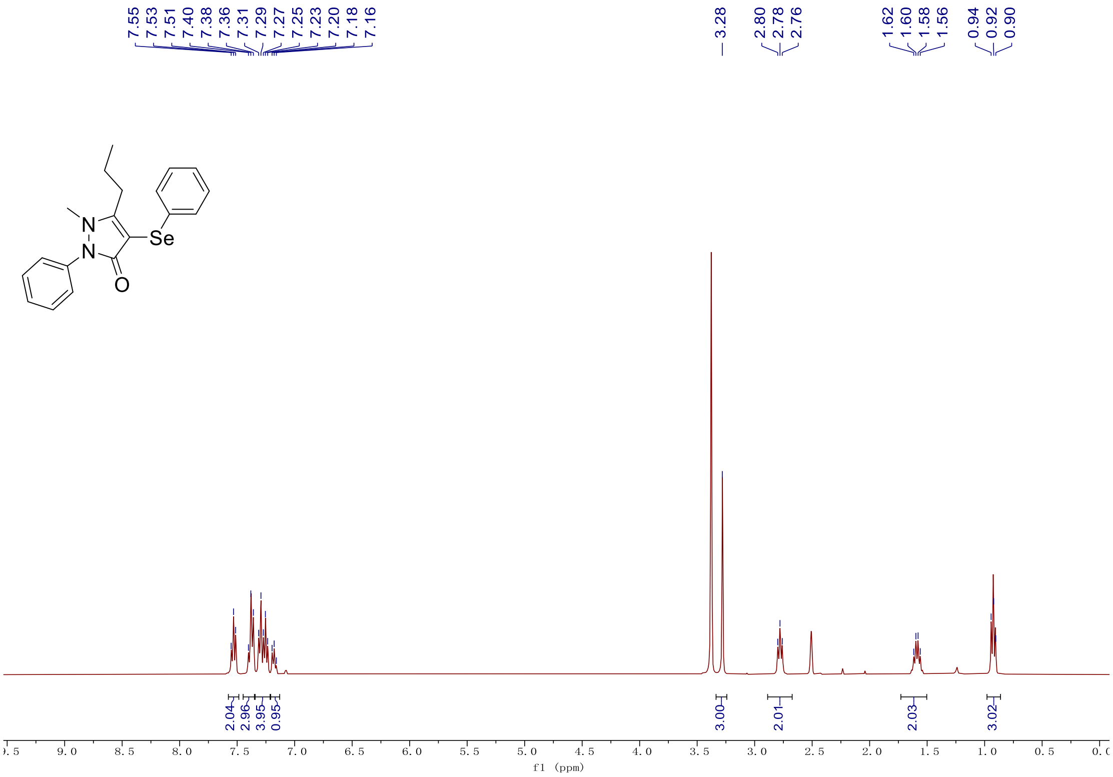

^{13}C NMR (101 MHz, DMSO-d_6) of compound **3zc**

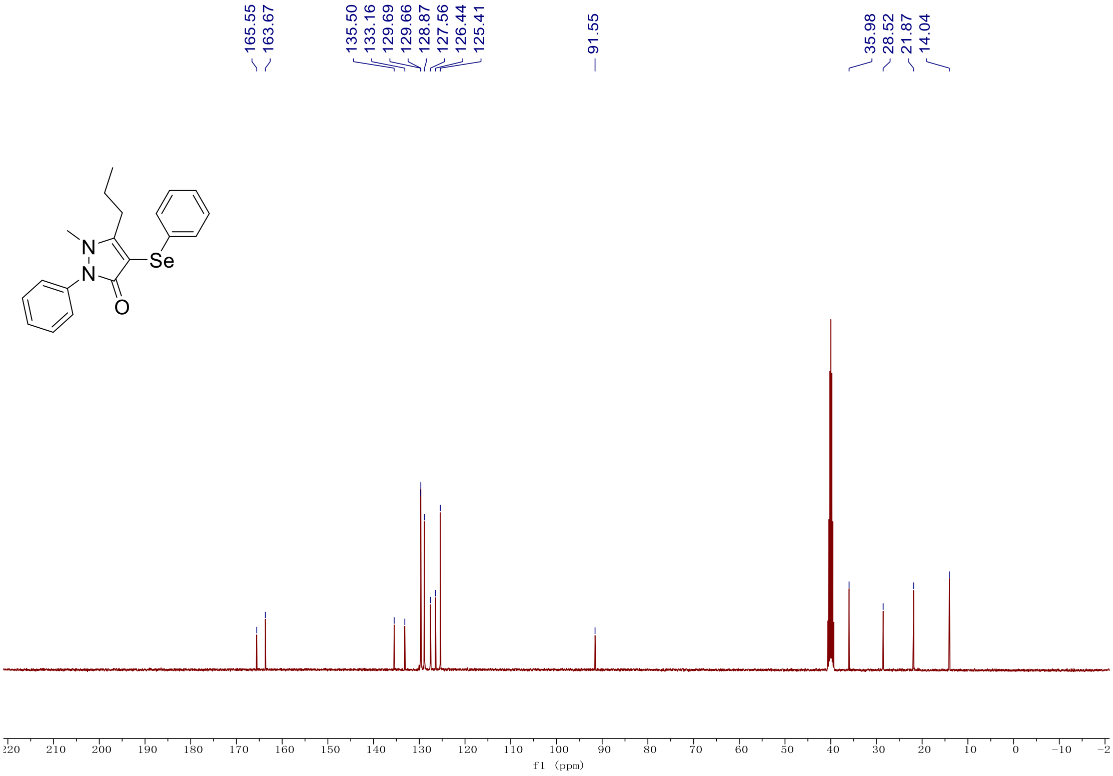

^{1}H NMR (400 MHz, $CDCl_3$) of compound **3zd**

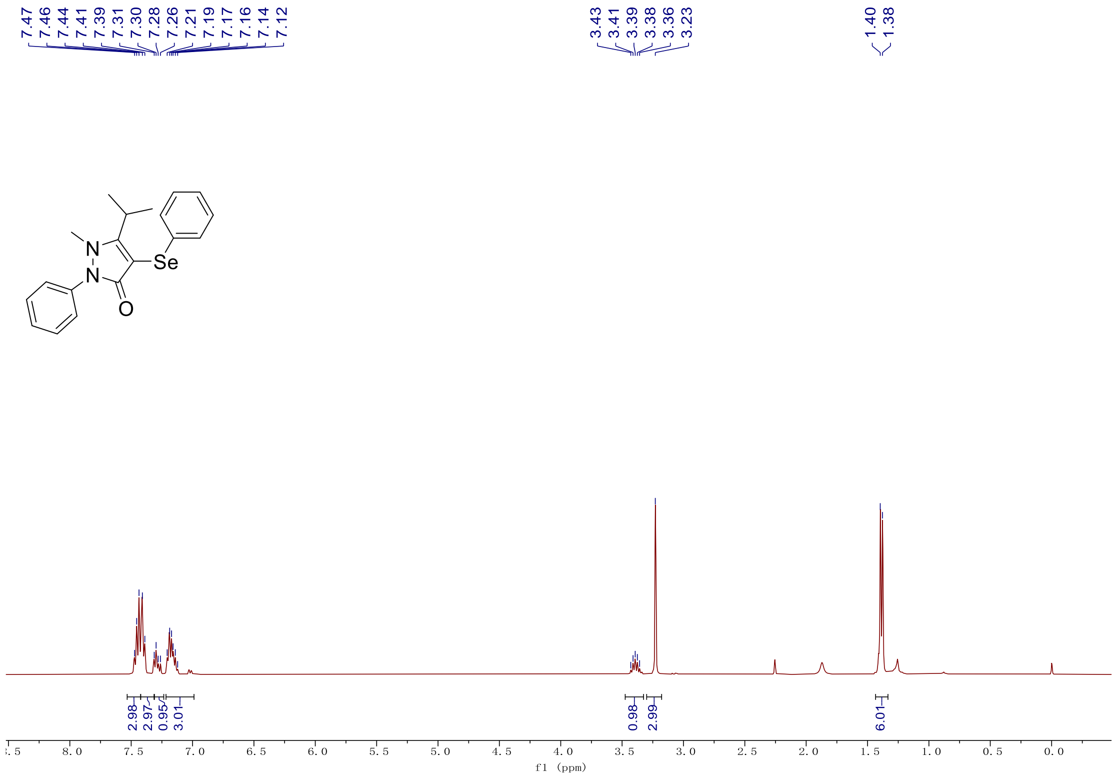

^{13}C NMR (101 MHz, $CDCl_3$) of compound **3zd**

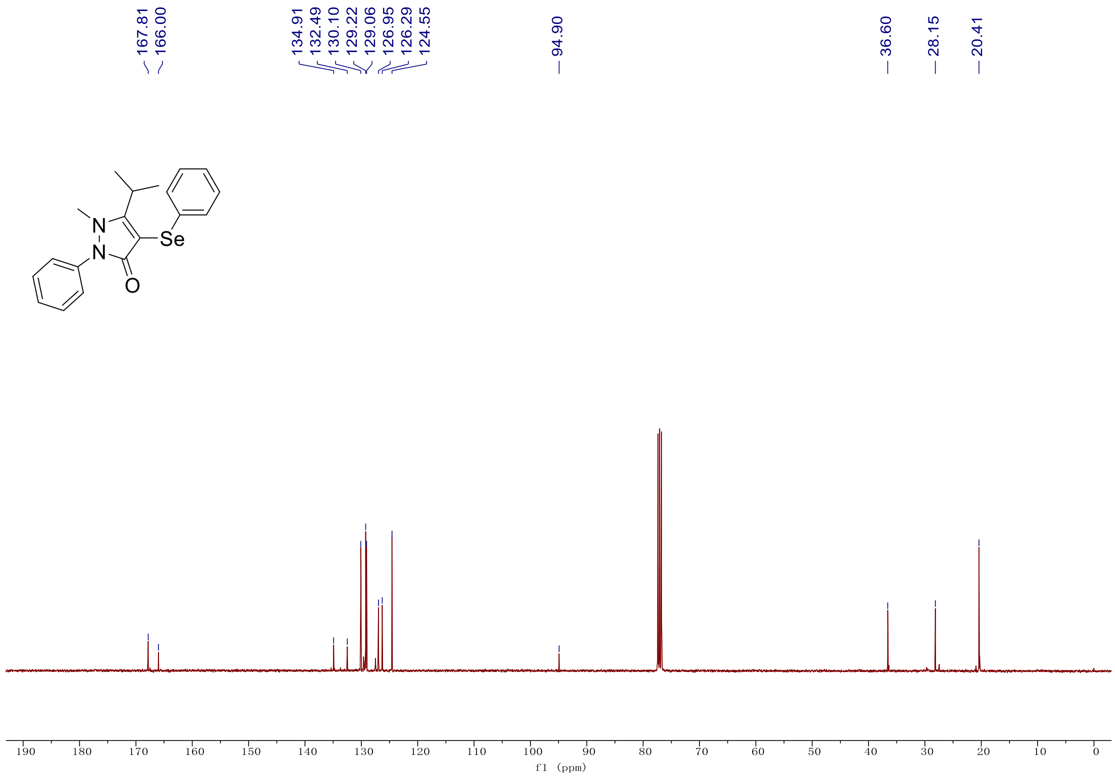

^{1}H NMR (600 MHz, DMSO-d_6) of compound **3ze**

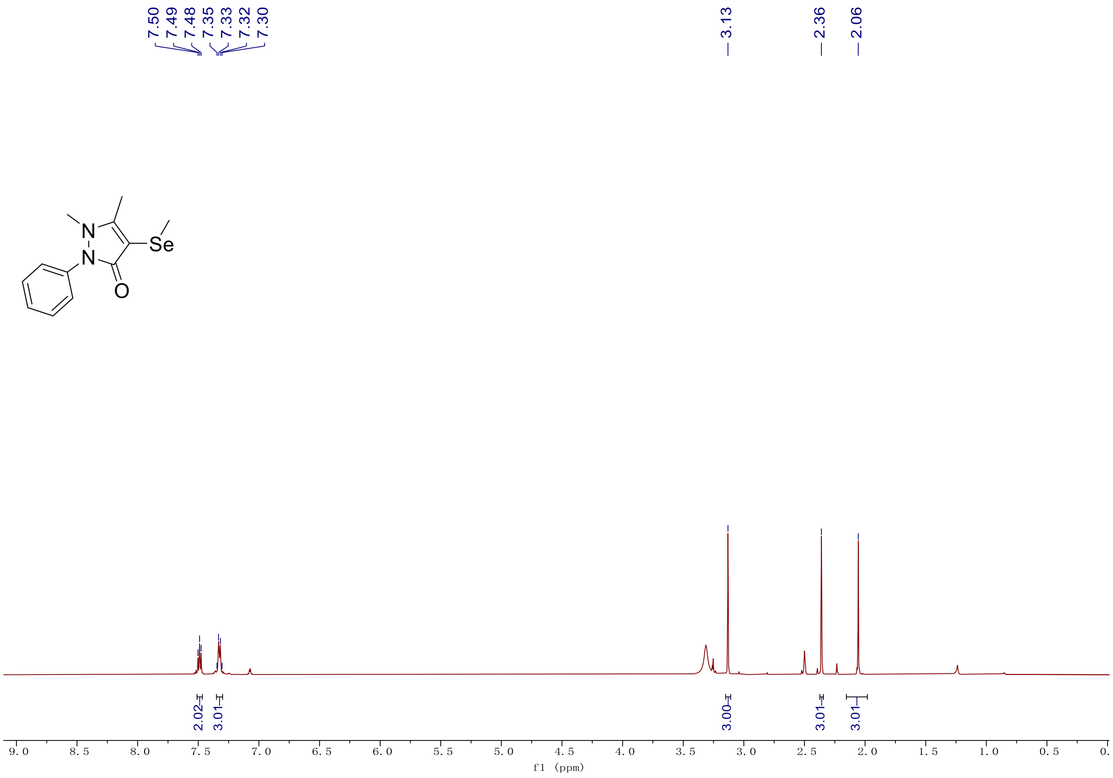

^{13}C NMR (151 MHz, DMSO-d_6) of compound **3ze**

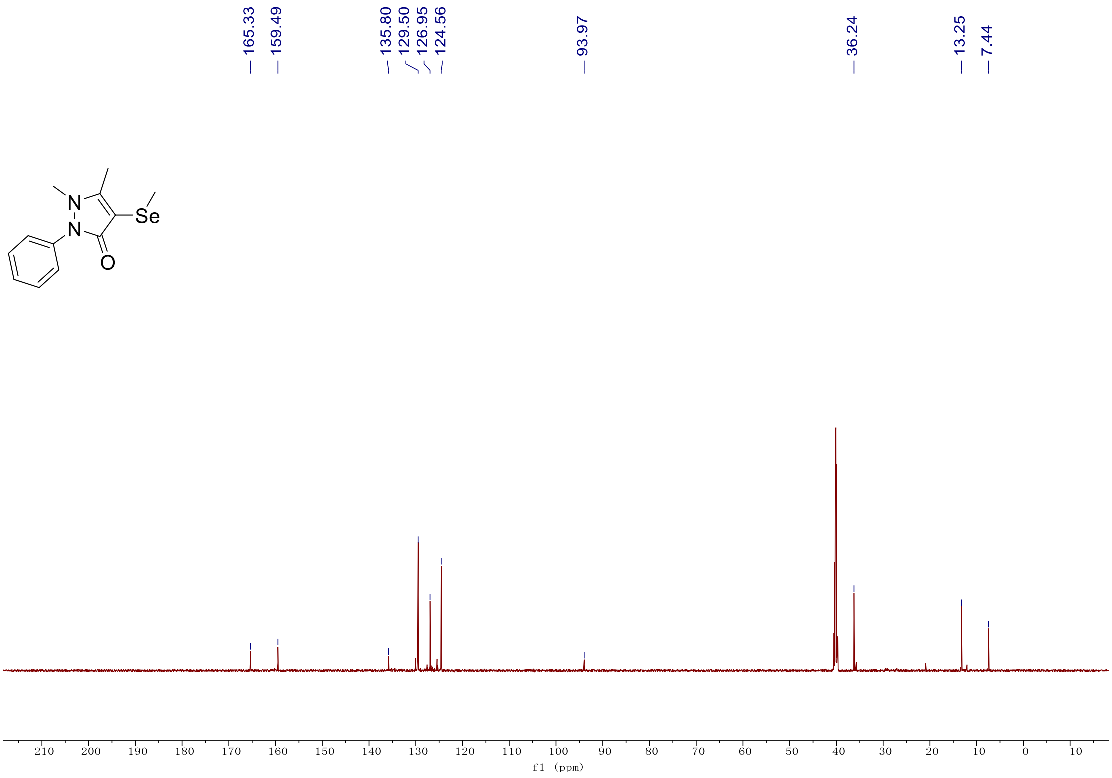

^{1}H NMR (600 MHz, DMSO-d_6) of compound **3zf**

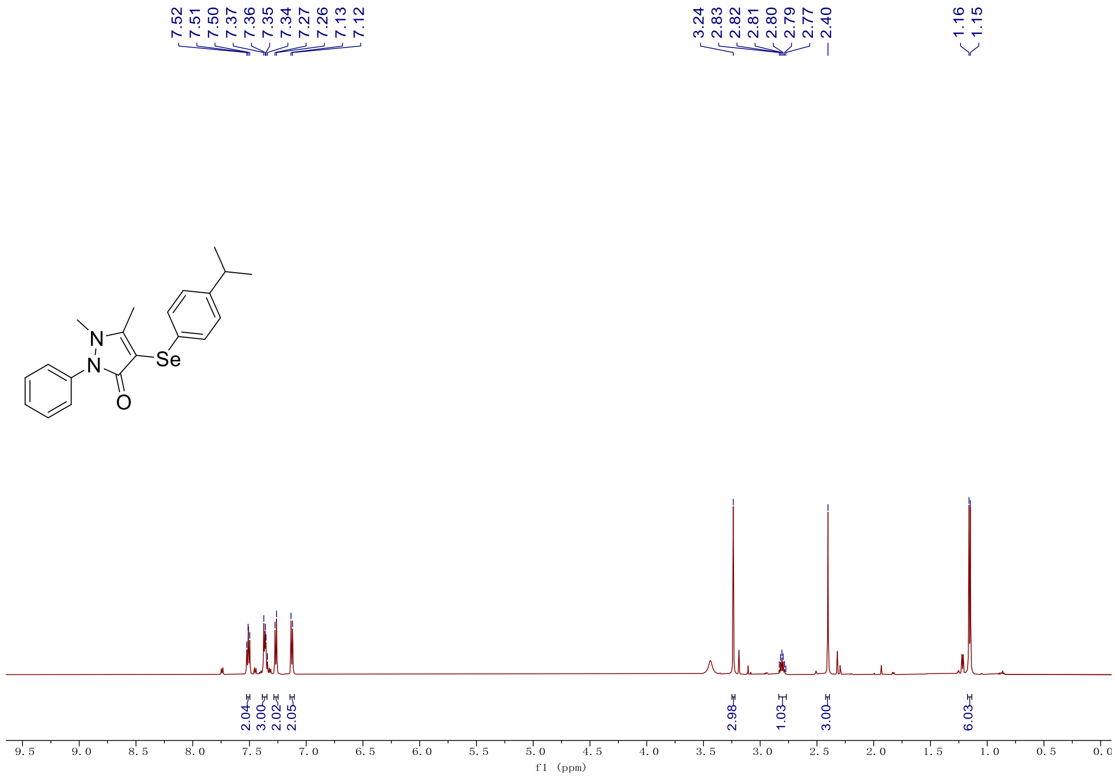

^{13}C NMR (151 MHz, DMSO-d_6) of compound **3zf**

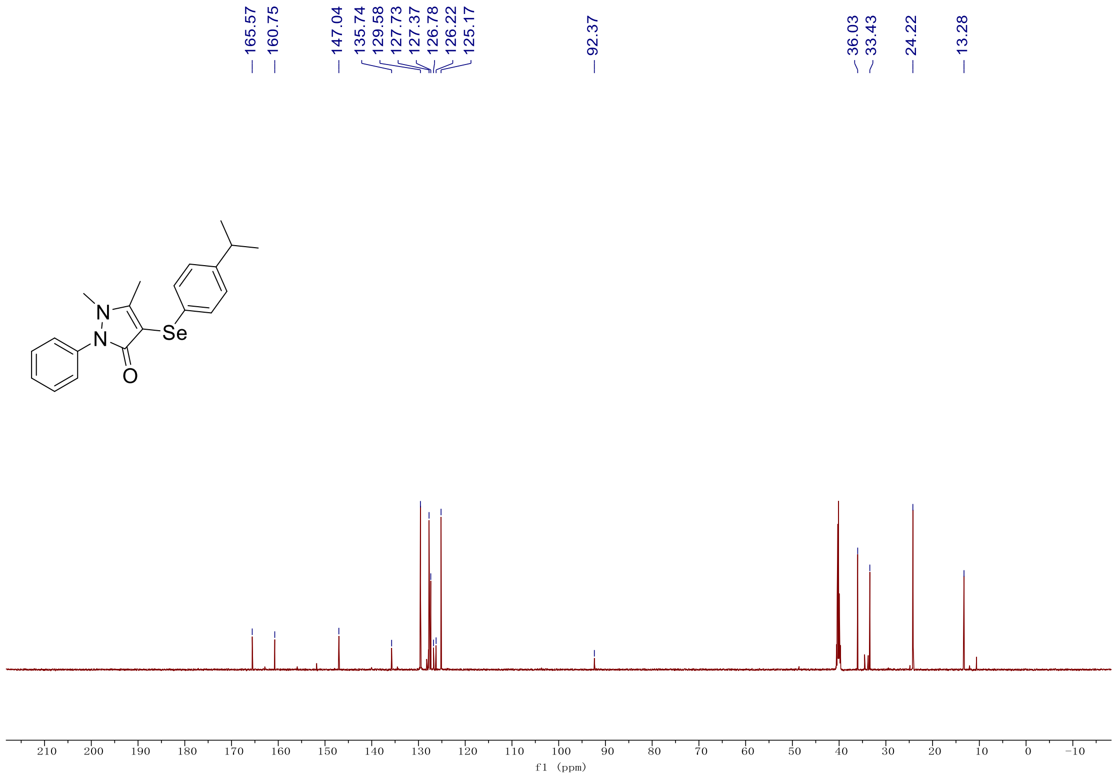

^{1}H NMR (600 MHz, $CDCl_3$) of compound **3zg**

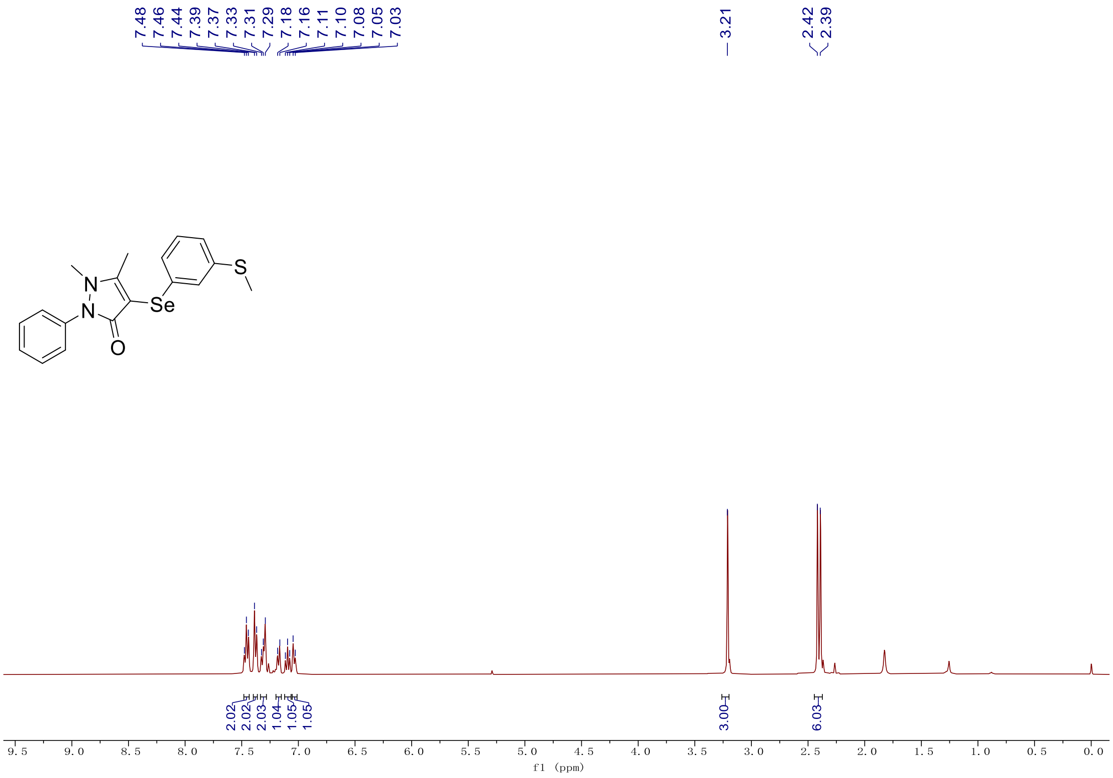

^{13}C NMR (151 MHz, $CDCl_3$) of compound **3zg**

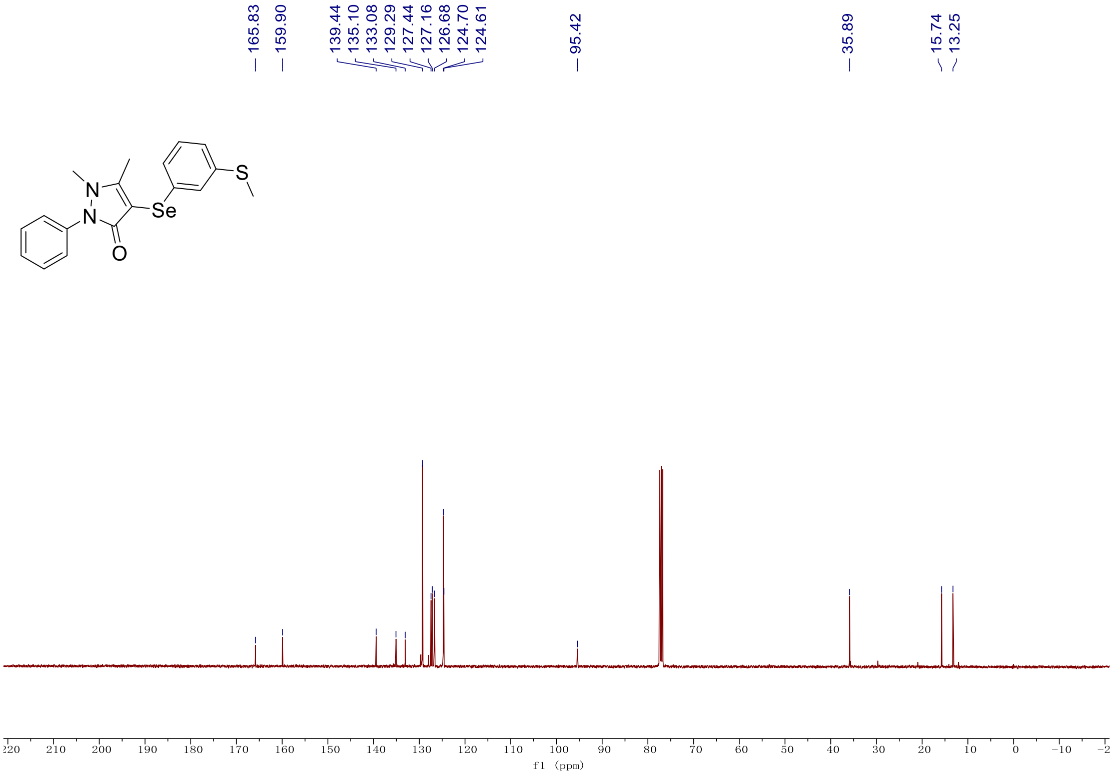

^{1}H NMR (600 MHz, DMSO-d_6) of compound **3zh**

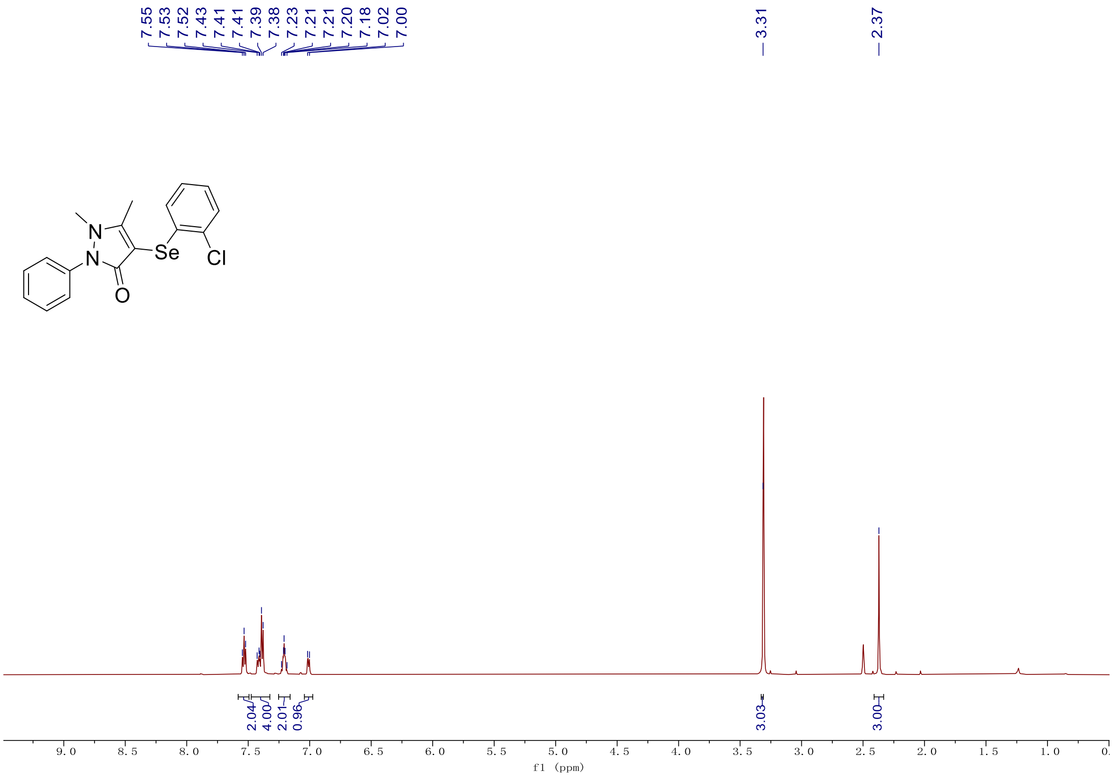

^{13}C NMR (151 MHz, DMSO-d_6) of compound **3zh**

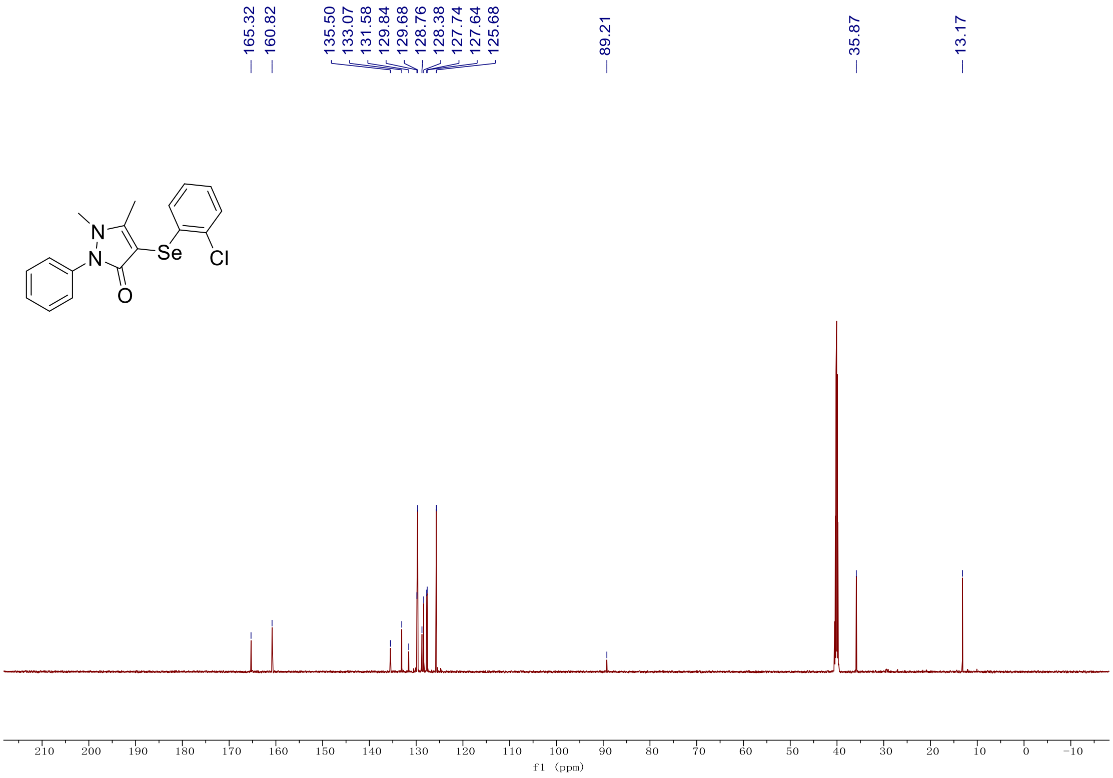

^{1}H NMR (600 MHz, DMSO-d_6) of compound **3zi**

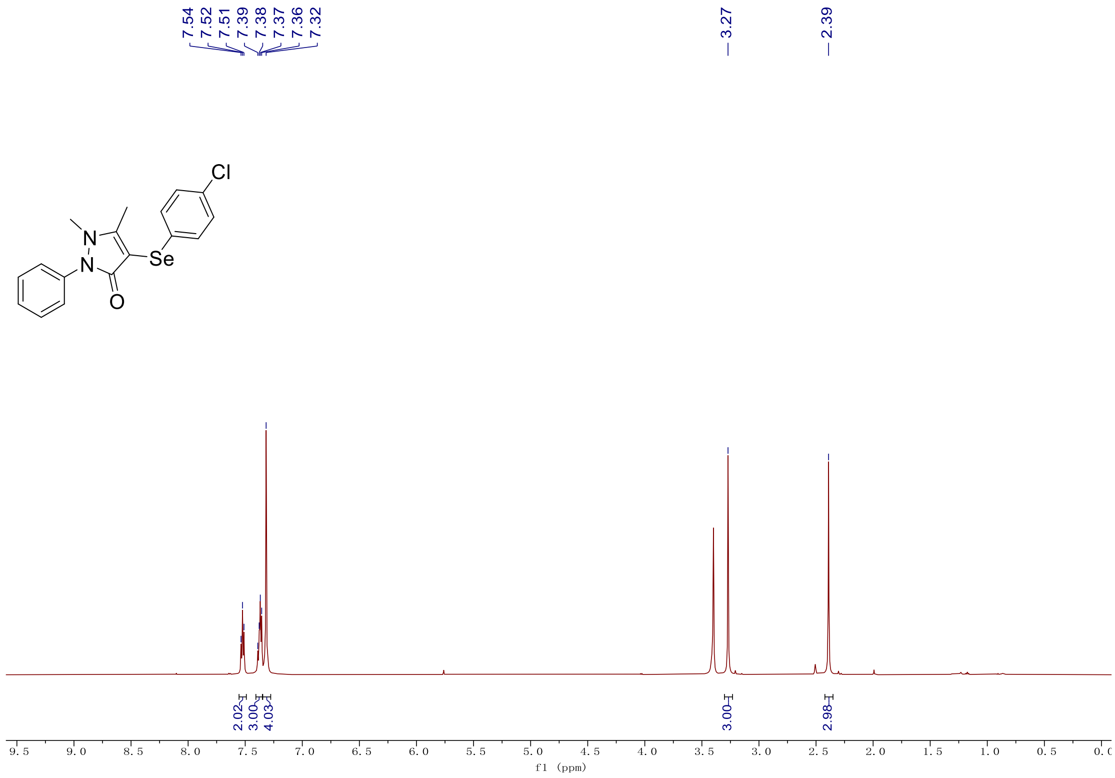

^{13}C NMR (151 MHz, DMSO-d_6) of compound **3zi**

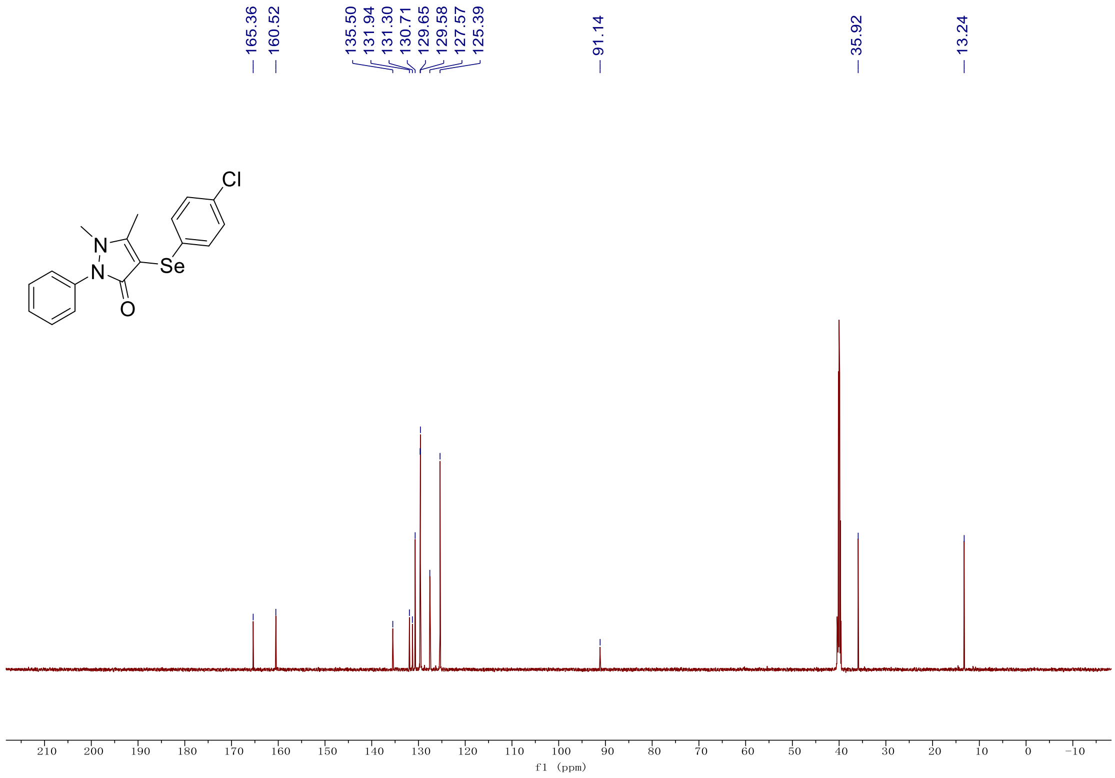

^{1}H NMR (400 MHz, DMSO-d_6) of compound **3zj**

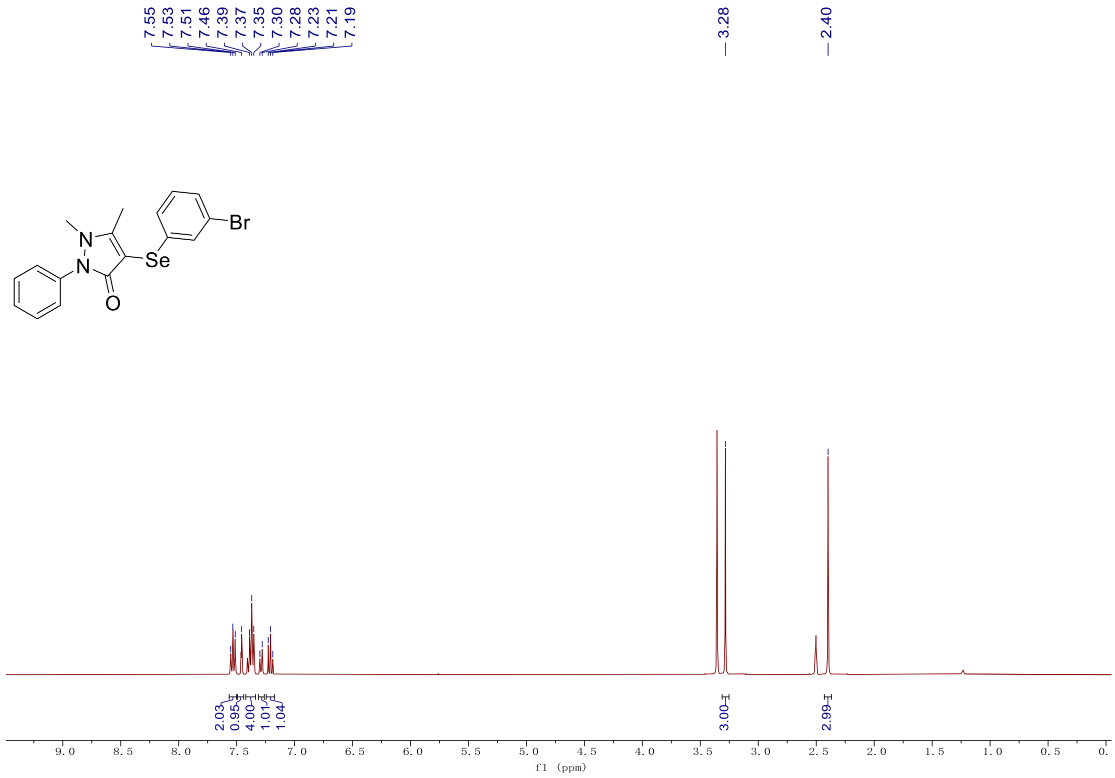

^{13}C NMR (101 MHz, DMSO-d_6) of compound **3zj**

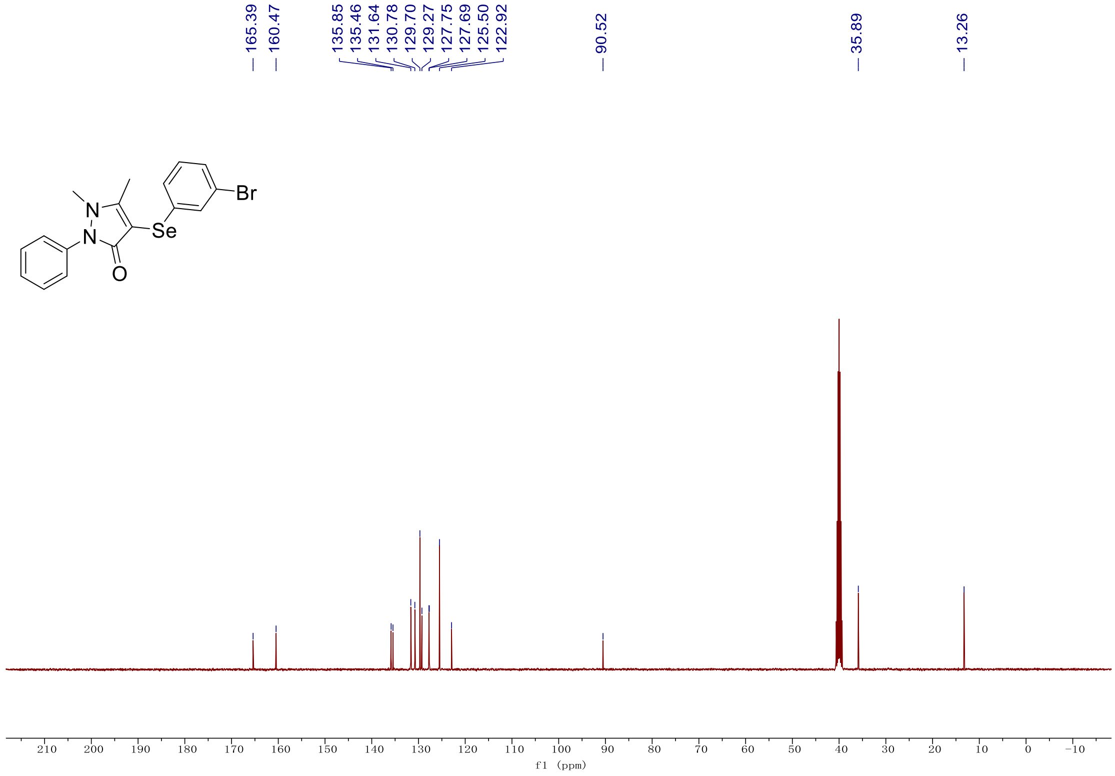

^{1}H NMR (600 MHz, DMSO-d_6) of compound **3zk**

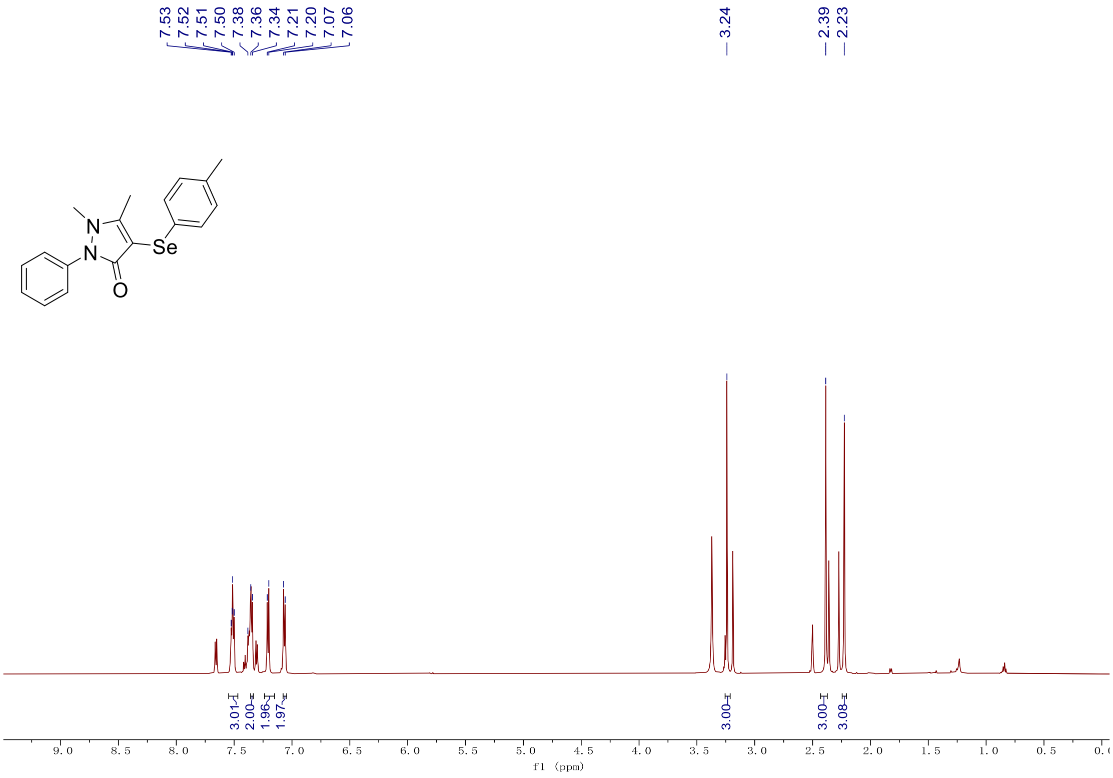

^{13}C NMR (151 MHz, DMSO-d_6) of compound **3zk**

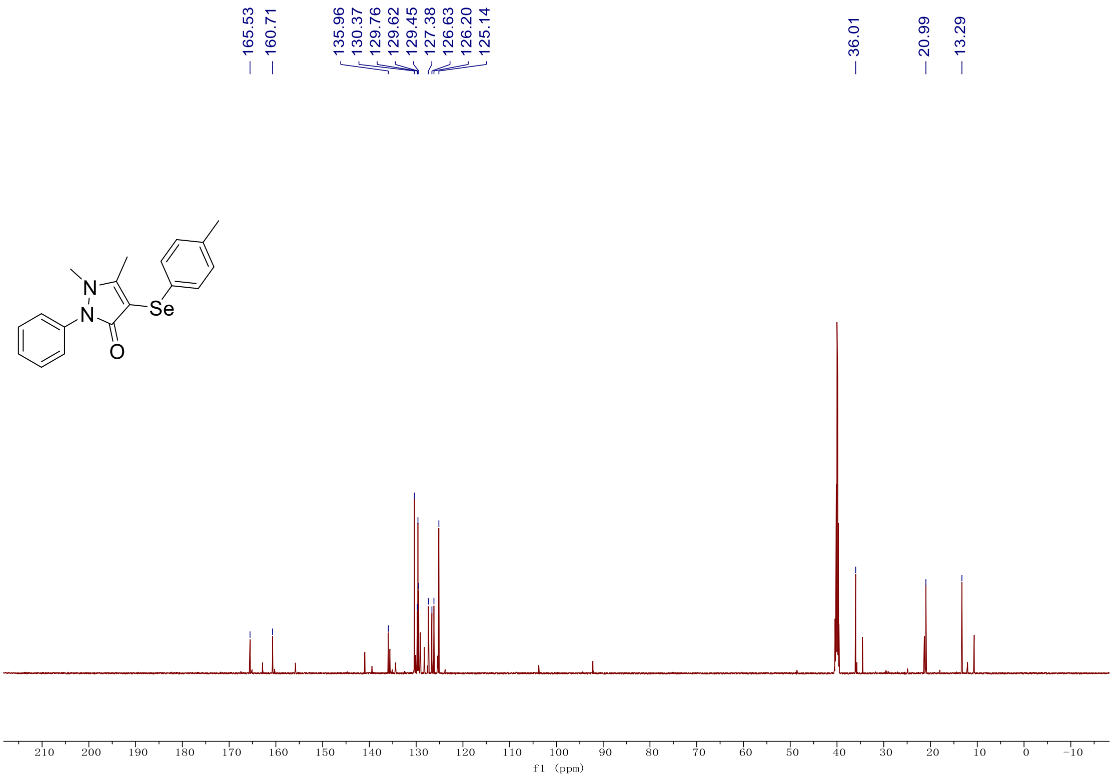

^{1}H NMR (400 MHz, DMSO-d_6) of compound **5a**

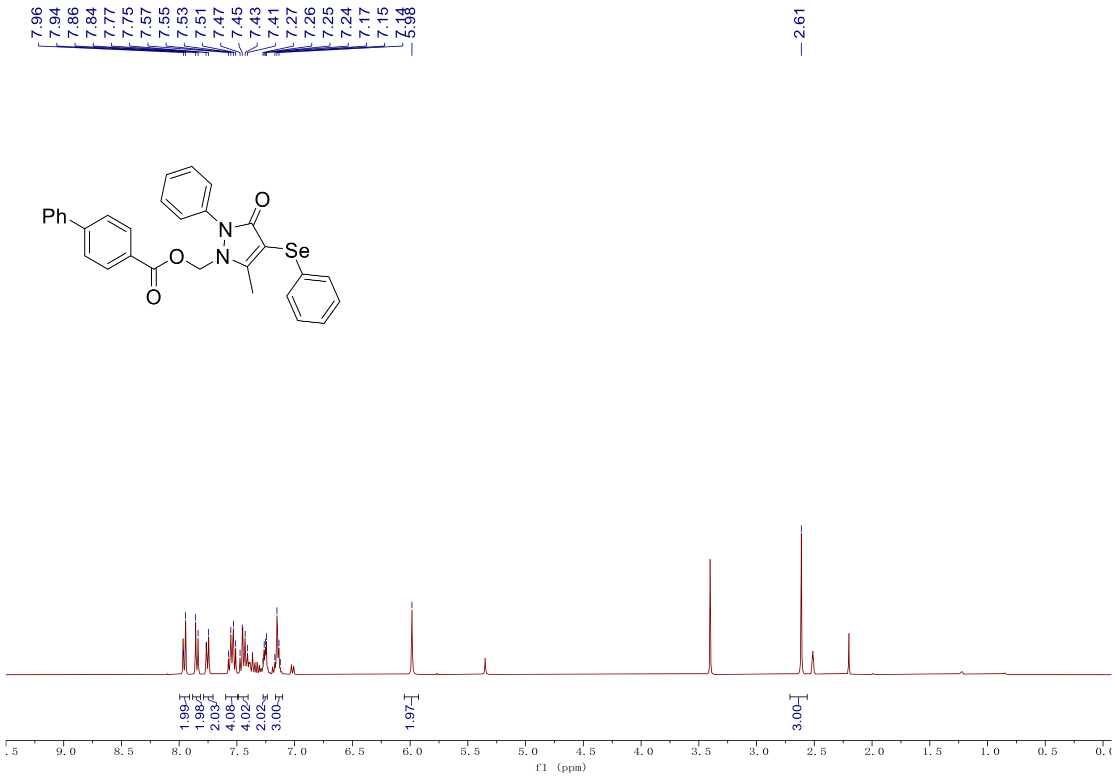

^{13}C NMR (101 MHz, DMSO-d_6) of compound **5a**

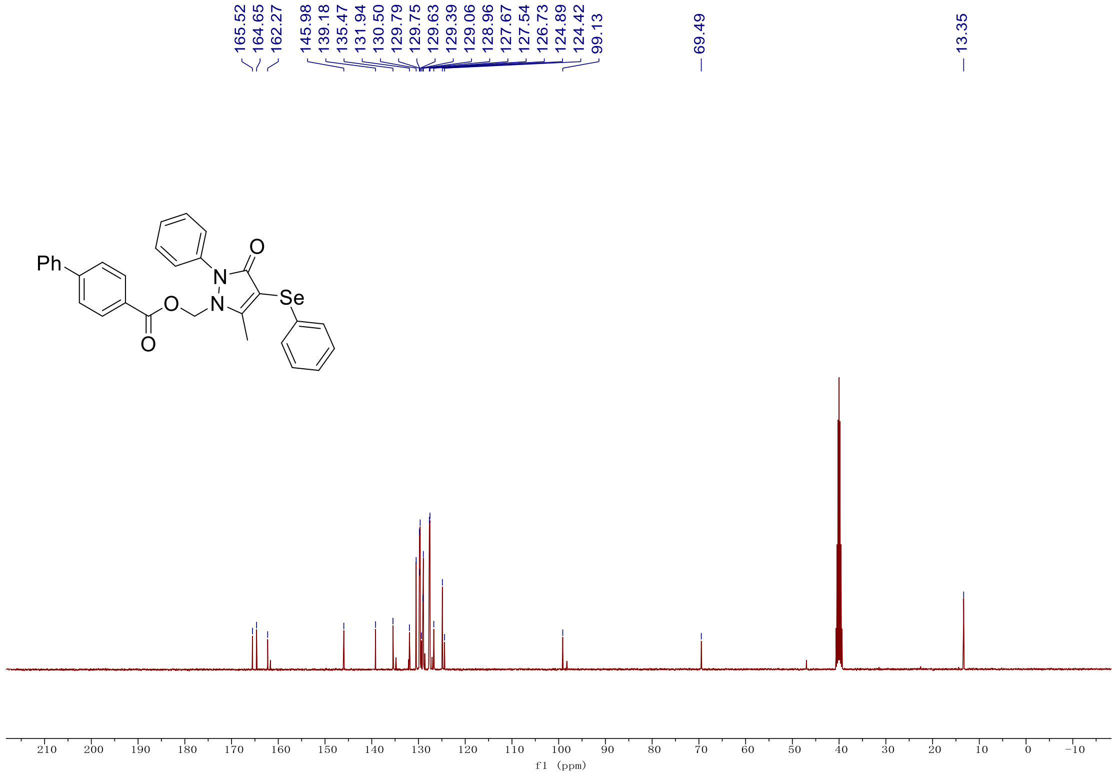

^{1}H NMR (400 MHz, DMSO-d_6) of compound **5b**

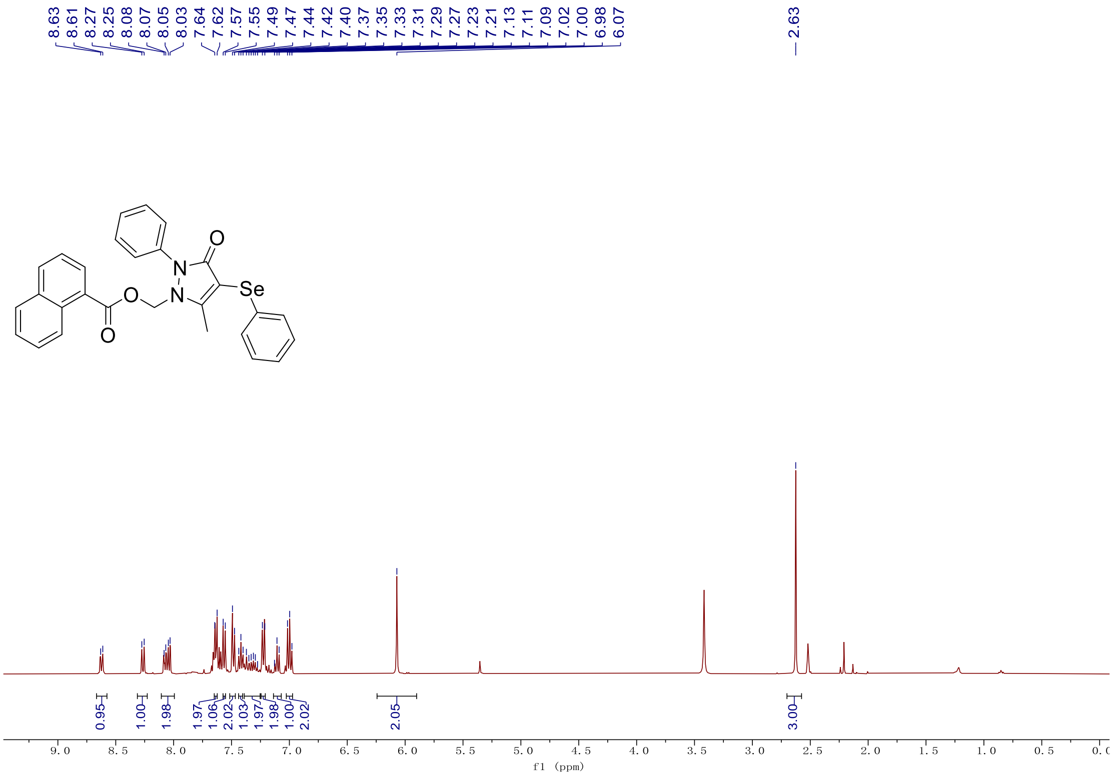

^{13}C NMR (101 MHz, DMSO-d_6) of compound **5b**

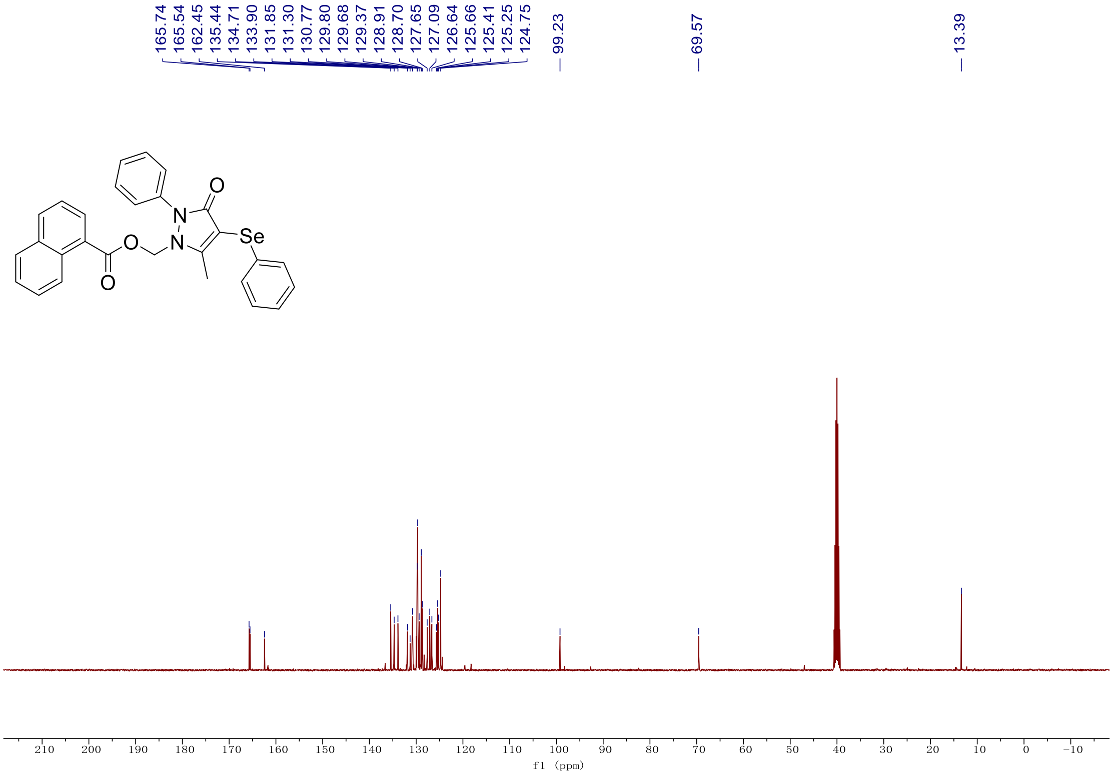

^{1}H NMR (400 MHz, DMSO-d_6) of compound **5c**

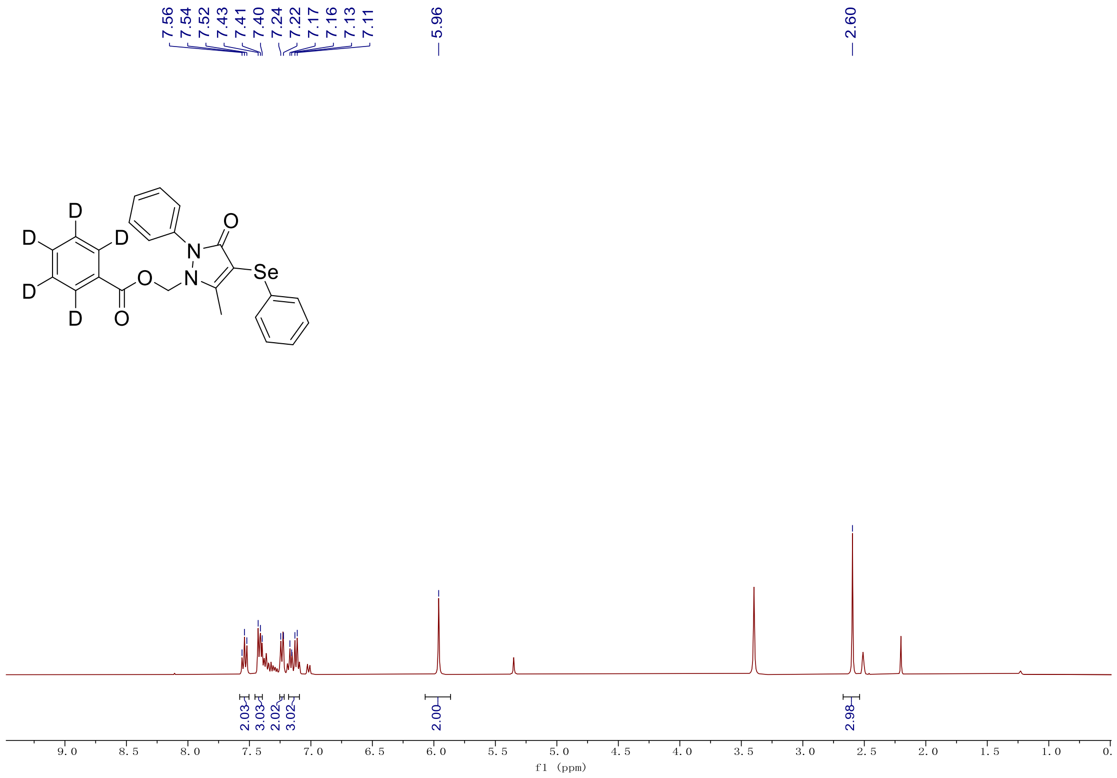

^{13}C NMR (101 MHz, DMSO-d_6) of compound **5c**

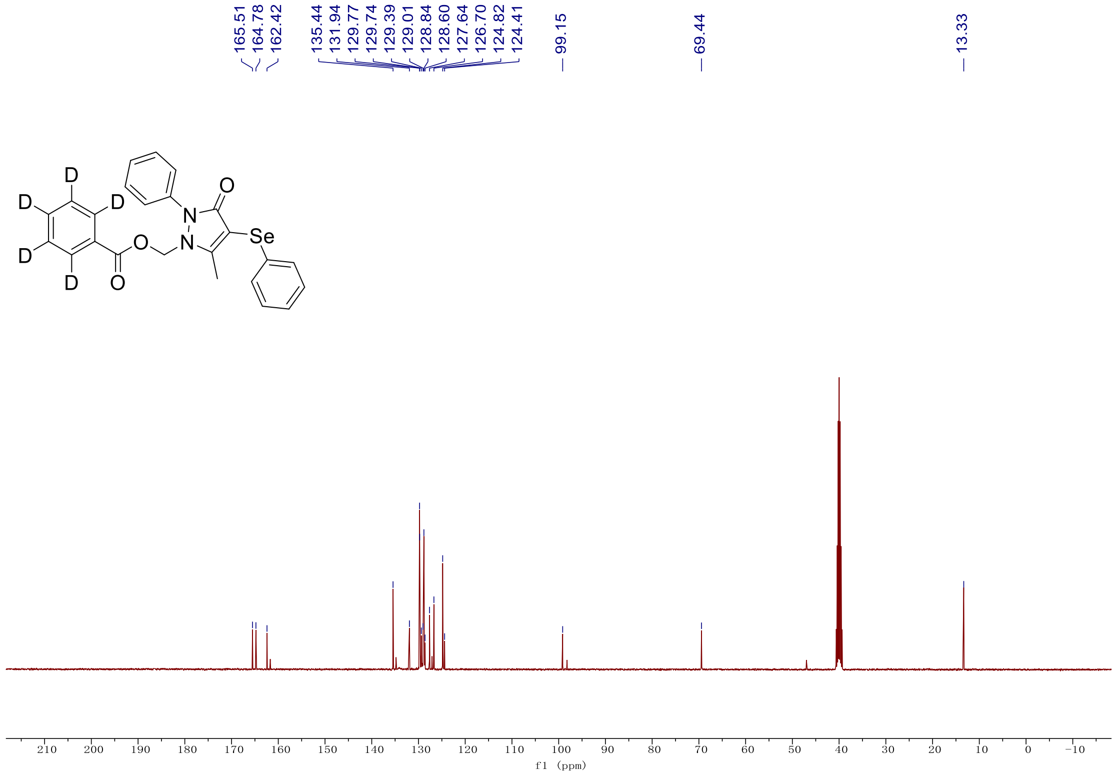

^{1}H NMR (400 MHz, DMSO-d_6) of compound **5d**

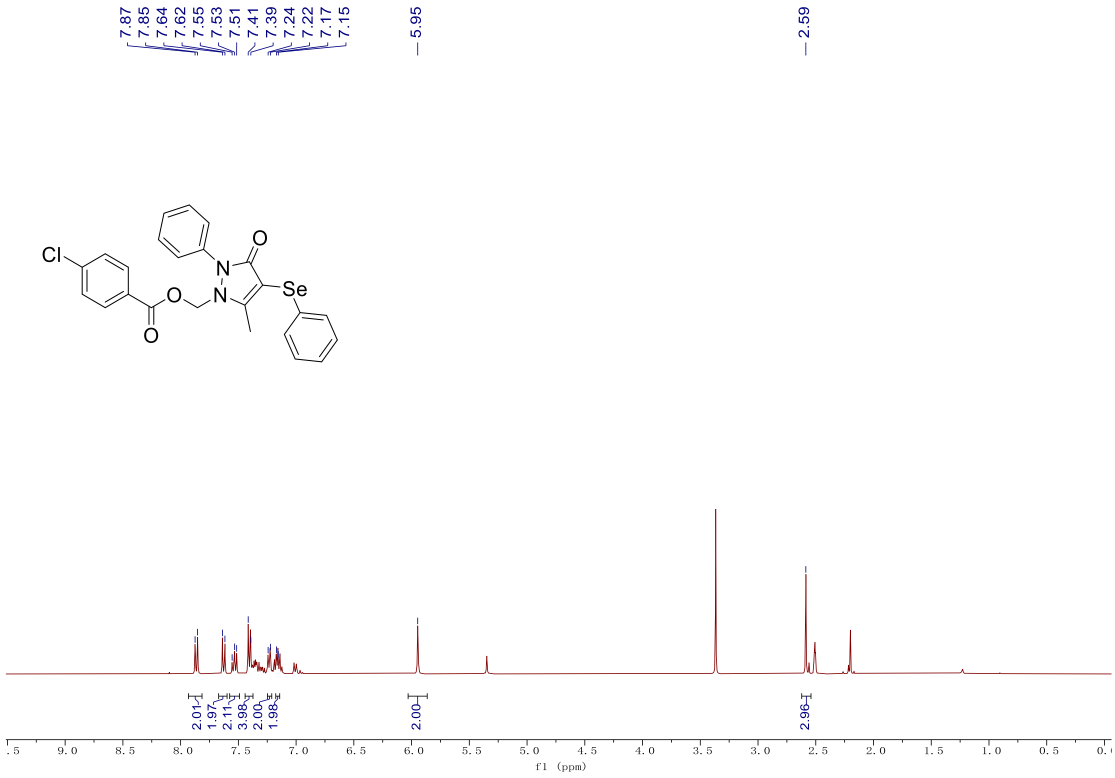

^{13}C NMR (101 MHz, DMSO-d_6) of compound **5d**

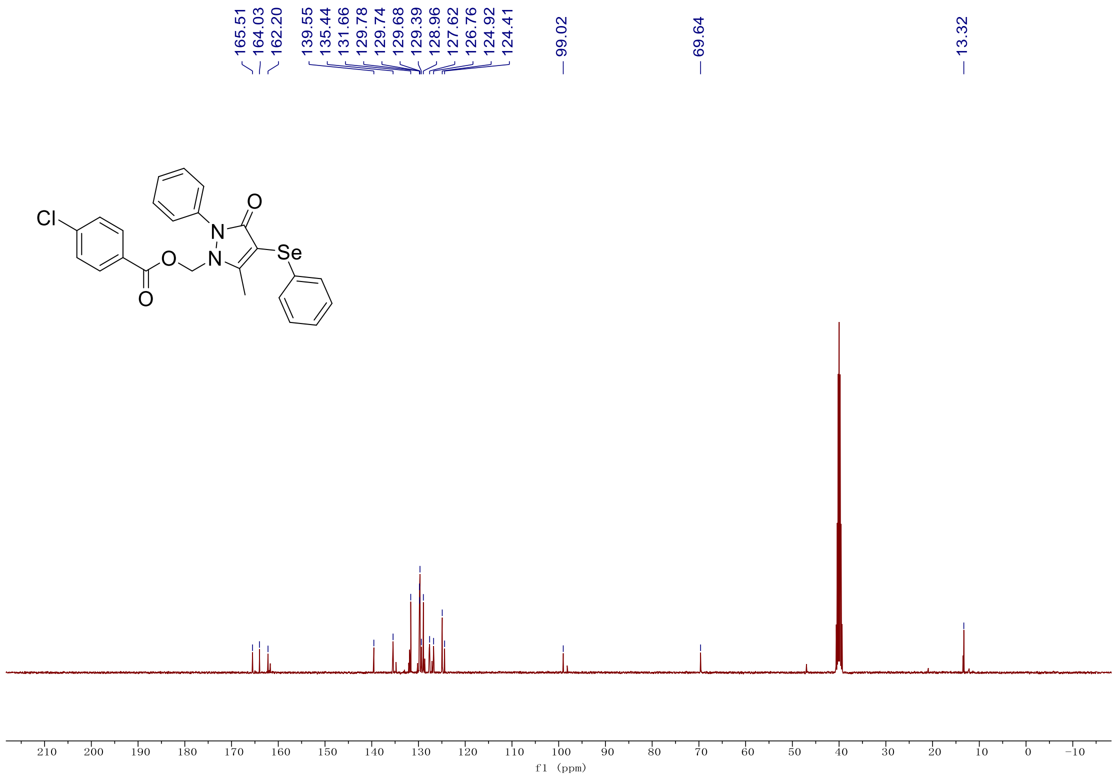

^{1}H NMR (400 MHz, DMSO-d_6) of compound **5e**

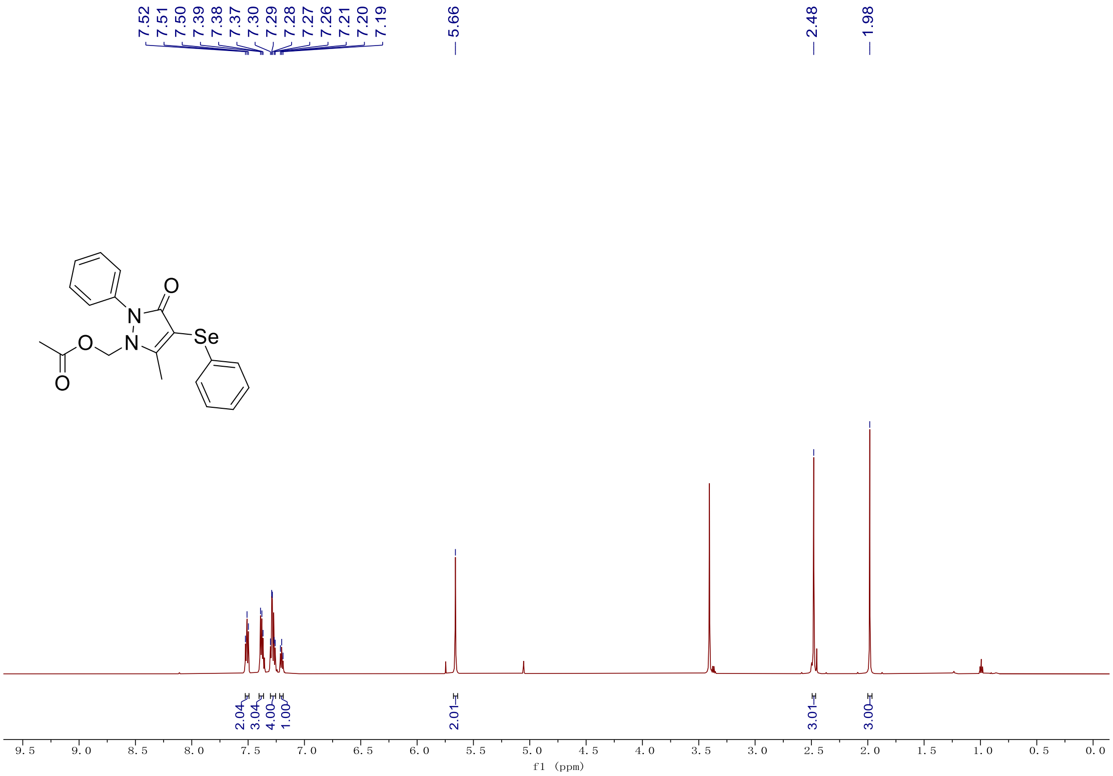

^{13}C NMR (101 MHz, DMSO-d_6) of compound **5e**

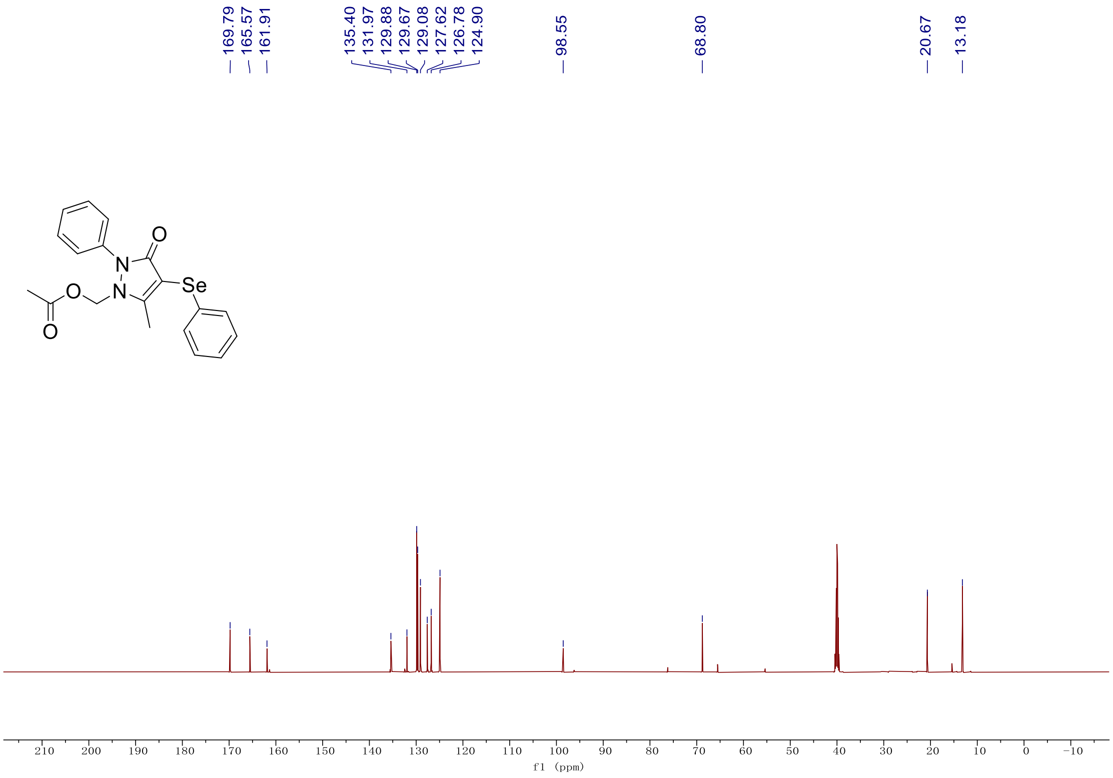

Supplement: RA-OLF-D6RA01336H-s001 [file RA-OLF-D6RA01336H-s001.pdf]
